# Overview of *Salmonella* Genomic Island 1-Related Elements Among Gamma-Proteobacteria Reveals Their Wide Distribution Among Environmental Species

**DOI:** 10.3389/fmicb.2022.857492

**Published:** 2022-04-11

**Authors:** Eliane Siebor, Catherine Neuwirth

**Affiliations:** ^1^Laboratory of Bacteriology, University Hospital of Dijon, Dijon, France; ^2^UMR-CNRS 6249 Chrono-Environnement, University of Burgundy - Franche-Comté, Besançon, France

**Keywords:** *Salmonella* genomic island 1, gamma-proteobacteria, environmental bacteria, *Vibrio*, *Shewanella*, *Halomonas*, *Idiomarina*, *Marinobacter*

## Abstract

The aim of this study was to perform an *in silico* analysis of the available whole-genome sequencing data to detect syntenic genomic islands (GIs) having homology to *Salmonella* genomic island 1 (SGI1), analyze the genetic variations of their backbone, and determine their relatedness. Eighty-nine non-redundant SGI1-related elements (SGI1-REs) were identified among gamma-proteobacteria. With the inclusion of the thirty-seven backbones characterized to date, seven clusters were identified based on integrase homology: SGI1, PGI1, PGI2, AGI1 clusters, and clusters 5, 6, and 7 composed of GIs mainly harbored by waterborne or marine bacteria, such as *Vibrio*, *Shewanella*, *Halomonas*, *Idiomarina*, *Marinobacter*, and *Pseudohongiella*. The integrase genes and the backbones of SGI1-REs from clusters 6 and 7, and from PGI1, PGI2, and AGI1 clusters differed significantly from those of the SGI1 cluster, suggesting a different ancestor. All backbones consisted of two parts: the part from *attL* to the origin of transfer (*oriT*) harbored the DNA recombination, transfer, and mobilization genes, and the part from *oriT* to *attR* differed among the clusters. The diversity of SGI1-REs resulted from the recombination events between GIs of the same or other families. The *oriT* appeared to be a high recombination site. The multi-drug resistant (MDR) region was located upstream of the resolvase gene. However, most SGI1-REs in *Vibrio*, *Shewanella*, and marine bacteria did not harbor any MDR region. These strains could constitute a reservoir of SGI1-REs that could be potential ancestors of SGI1-REs encountered in pathogenic bacteria. Furthermore, four SGI1-REs did not harbor a resolvase gene and therefore could not acquire an integron. The presence of mobilization genes and AcaCD binding sites indicated that their conjugative transfer could occur with helper plasmids. The plasticity of SGI1-REs contributes to bacterial adaptation and evolution. We propose a more relevant classification to categorize SGI1-REs into different clusters based on their integrase gene similarity.

## Introduction

Genomic islands (GIs) are large segments of DNA detected in the bacterial chromosome when comparing closely related strains, indicating that they have been horizontally transferred. The GIs have the following characteristics: their guanine (G) + cytosine (C) content usually differs from that of the host chromosome; they are often inserted into a specific chromosomal site, particularly in the tRNA genes, and contain a recombination module consisting of an integrase and two attachment sites (*attL* and *attR*) located at each end of the GI (Juhas et al., 2009). The tRNA-modification GTPase gene (named *trmE*, *mnmE*, or *thdF*) characterized in Enterobacteriales and other Gram-negative bacteria, such as *Acinetobacter baumannii* or *Vibrio cholerae*, is the target for integrative mobilizable elements (IMEs) related to *Salmonella* genomic island 1 (SGI1) (Doublet et al., 2007). The SGI1 (Boyd et al., 2001) and its relative islands named *Proteus* genomic island 1 (PGI1) (Siebor and Neuwirth, 2014), PGI2 (Lei et al., 2018), and *Acinetobacter* genomic island 1 (AGI1) (Hamidian et al., 2015) belong to the same family of GIs, the SGI1-related elements (SGI1-REs). They consist of a backbone composed of 26 to 28 open reading frames (ORFs) and a multidrug resistance (MDR) region that usually contains a complex class 1 integron and sometimes transposons. The gene synteny is highly conserved among the SGI1/PGI1/PGI2/AGI1 backbones. All of them harbor a DNA recombination module (*int* and *xis* genes) encoding an integrase belonging to the tyrosine-recombinase family and an excisionase, a replication module consisting of a *rep* gene encoding a replicase and an ORF encoding a transcriptional regulator, transcriptional activator genes (*sgaC* and usually *sgaD*) encoding the sgaDC heterodimeric complex belonging to the FlhDC family, some genes involved in conjugation (*traN*, *traG*, and usually *traH*), and mobilization genes (*mpsB*/*mpsA*) (de Curraize et al., 2021). The SGI1-REs are not able to self-transfer; however, they harbor AcaCD binding sites indicating that they are mobilizable by IncC conjugative plasmids (Douard et al., 2010; Carraro et al., 2014, 2017; Siebor et al., 2016; Lei et al., 2018; Siebor and Neuwirth, 2020). The origin of transfer (*oriT*) is located upstream of *mpsB*/*mpsA* (Kiss et al., 2019). The MDR region is usually inserted upstream of a resolvase gene (*res*) and flanked by a 5-bp duplication, suggesting that it was acquired by transposition.

Many SGI1/PGI1/PGI2/AGI1 variants have been reported, resulting from variations in the MDR region due to cassette exchanges in the integron, IS-mediated transposition events, or the insertion of transposons (Hall, 2010; de Curraize et al., 2018, 2020b). The MDR region is generally inserted at the same position in SGI1 variants, PGI1 variants, and PGI2 variants. Nevertheless, five positions were detected in AGI1 variants (Siebor and Neuwirth, 2020). Some differences also occur in their backbone. The most-reported variation in the backbone of SGI1 variants is the deletion from within the *traN* to within S009 replaced by the insertion sequence IS*1359* (IS*Vch4*) characterizing the SGI1-HKL group (de Curraize et al., 2020b), and other deletions on either side of the MDR region insertion site often resulting from IS*26*-mediated events. A deletion also occurs between A022 and A025 in AGI1 variants, replaced by the insertion of a new ORF sharing nucleotide (nt) identity with a short segment from a *Shewanella* element that is a part of a GI that belongs to the same SGI1-RE family (Siebor and Neuwirth, 2020; de Curraize et al., 2021). However, there are some differences among the SGI1/PGI1/PGI2/AGI1 backbones that are located upstream of the mobilization genes and the *oriT*. Genes encoding a toxin/antitoxin system consisting of a subtilisin-like protease and an AAA-ATPase family protein were found downstream of the *res* gene in SGI1 (ORFs S025 and S026 called *sgiT* and *sgiA*, respectively) and in PGI1 [ORFs P022 (*pgiT*) and P023 (*pgiA*)] (Siebor and Neuwirth, 2014); whereas in AGI1, the ORFs, A025, and A026 encode a BsuB1-Pst1 restriction endonuclease and an N-6 DNA methylase, respectively (Hamidian et al., 2015). The *res* gene is absent from PGI2, its MDR region being adjacent to the ORF PGI2-024 (Lei et al., 2018). ORFs SO23 and SO24, which encode a helicase and an exonuclease, are found only in the SGI1 variants, except for SGI1-V (Siebor and Neuwirth, 2011).

*Salmonella* genomic island 1-related elements carrying antimicrobial resistance genes have been found mainly in the pathogenic strains of human or veterinary samples (Siebor and Neuwirth, 2020; de Curraize et al., 2021). Only a few SGI1-REs that do not harbor any MDR region have been reported. The SGI0 found in *Proteus mirabilis* might be a progenitor related to SGI1 (de Curraize et al., 2020a). An SGI1-RE identified in *Klebsiella pneumoniae* consists of a region closely related to PGI2 and in parts closer to PGI1 (Cummins et al., 2020). Most of an SGI1-RE found in *Shewanella* is closer to the AGI1 backbone (Siebor and Neuwirth, 2020), and SGI1-REs found in *Vibrio cholerae* and *Vibrio mimicus* are closer to the PGI1 backbone (de Curraize et al., 2021). Given that SGI1/PGI1/PGI2/AGI1 islands integrate at the 3′-end of the *trmE* gene and that many bacterial species carry the *trmE* gene, we suspect that SGI1-REs might be more widely distributed and that SGI1-REs with a new backbone might perhaps emerge. To support this hypothesis, we explored GenBank^®^. Currently, thanks to the microbial whole-genome sequencing (WGS) database contains lot of data. They consisted of complete genomic sequences or partial genomes made up of multiple unassembled contigs. Identification of resistance genes and other specific sequences can be achieved using the basic local alignment search tool (BLAST). Here we report the results of an *in silico* analysis of SGI1-REs exhibiting homology with the backbones of SGI1/PGI1/PGI2/AGI1 islands. Only SGI1-REs, whose backbones could be completely assembled, were studied. We examined these SGI1-REs by searching for variations in these uncharacterized backbones. A single representative backbone was selected when multiple strains of the same species contained an identical backbone. Phylogenetic analyses were performed by including 37 distinct backbones described to date, for which the complete sequence was deposited, to determine the phylogenetic relationships between them. This study will expand the knowledge of SGI1-RE distribution among Gram-negative bacteria and their relatedness.

## Materials and Methods

### Bioinformatics Analysis

The SGI1/PGI1/PGI2/AGI1 islands were identified with the nucleotide BLAST in the National Center for Biotechnology Information (NCBI) databases (nucleotide collection and whole-genome shotgun contigs databases) available until March 31, 2021^1^ first using the integrase gene and second using the complete backbone of the GIs as a query sequence. We searched for SGI1/PGI1/PGI2/AGI1 islands in alpha-, beta- and gamma-proteobacteria and retained only GIs with syntenic genes. Redundant backbone nt sequences found in the strains of the same species were excluded. SGI1-REs with the same *int*_GI_ homology but with no or identical backbone variations (insertions, deletions, nt sequence exchanges, or ≥0.2% of nt substitutions) were retained when they were detected in different bacterial species to determine the distribution of the SGI1-REs. All identified uncharacterized GIs were analyzed using BLAST and the G+C content of the backbones was determined. The 18-bp attachment sites (*attL* and *attR*) were analyzed and the position of the MDR region, when present, was determined. Putative AcaCD-binding sites were predicted using FIMO (MEME suite) based on the AcaCD logo, as presented in the study^2^ of Carraro et al. (2014).

### Phylogenetic Analysis

Multiple alignments of nucleotide sequences, including the 37 fully characterized backbones to date and the uncharacterized GIs retained for this study, was performed using the online MAFFT software version 7 available at the Japanese Computational Biology Research Center (CBRC)^3^. The 37 known SGI1-REs included 18 SGI1 variants: SGI1 (Boyd et al., 2001), SGI1-K1 (Levings et al., 2007), SGI2 (Levings et al., 2008), SGI1-V (Siebor and Neuwirth, 2011) recently renamed SGI-V (de Curraize et al., 2020a), SGI1-*Pm*MAT (Siebor and Neuwirth, 2013), SGI1-B2 and SGI1-Z (Lei et al., 2015), SGI1-L (Schultz et al., 2017), SGI1-*Pm*2CHAMA (de Curraize et al., 2018), SGI1-*Pm*JN40 (Bie et al., 2018), SGI1B-*Ec*1 (Cummins et al., 2019), SGI1-*Pm*SC1111 (Wang et al., 2019), SGI1-*Pm*CA11 (Xiao et al., 2019), SGI0 (de Curraize et al., 2020a), SGI1-LK1 (de Curraize et al., 2020b), SGI1-*Vc*2CHAMA and VGI (Cummins et al., 2020), and SGI1-XJ9S (Lei et al., 2020a); 3 PGI1 variants: PGI1-*Pm*ESC (Siebor et al., 2016), the PGI1 variant of *Salmonella enterica* Heidelberg SL476 named here PGI1-SL476 (Siebor and Neuwirth, 2014), and PGI1-*Pm*PEL (Girlich et al., 2015); 3 PGI2 variants: PGI2 (Lei et al., 2018), PGI2-*Ec*-2, and KGI (Cummins et al., 2020); and 12 AGI1 variants belonging to 5 lineages: AGI1 (Hamidian et al., 2015), AGI1-A (renamed AGI2), AGI1-B and AGI1-C (renamed AGI3) (Siebor et al., 2019; Siebor and Neuwirth, 2020), AGI1 variants of *Escherichia coli* MOD1-EC6520, *K. pneumoniae* k1781 (AGI4), of 3 *Salmonella* [*S*. Agona 24.H.04 (AGI4), *S*. Cubana 76814 (AGI5) and *S*. Stanley DMS 1112 (AGI1 variant)], *V. cholerae* 133-73 (AGI2) and 4874 (AGI3), and *Vibrio* sp. 2017V-1124 (Siebor and Neuwirth, 2020). The IMESsW3-18-1 of *Shewanella* sp. W3-18-1 originally described as *Shewanella putrefaciens* was also included as a reference and was renamed SGI1-RE5*Ss*W3-18-1 (Qiu et al., 2013; Siebor and Neuwirth, 2020; de Curraize et al., 2021) (Supplementary Table 1). The relationships among these GIs were determined based on the phylogeny of their *int*_GI_ gene (Boyd et al., 2009). A phylogenetic analysis of the complete GI backbones was also performed. Neighbor-joining phylogenetic trees with 100 bootstrap replicates were generated from the multiple alignments and were viewed on Phylo.io accessed at CBRC webserver. The percent identity matrix of all *int*_GI_ genes and of the complete backbones of the SGI1-REs taken as a reference in each cluster was created by Clustal Omega^4^.

## Results

### Detection of *Salmonella* Genomic Island 1-Related Elements and Identification of Genomic Islands Clusters Based on the Phylogeny of *int*_*GI*_ Genes

In addition to the 37 fully characterized SGI1-REs, we detected 89 new and non-repetitive SGI1-REs that were identified among 7 bacterial orders belonging to gamma-proteobacteria (Supplementary Table 1). These new SGI1-REs were all inserted at the 3′-end of the *trmE* gene, as were all SGI1-REs described to date, except for PGI1-SL476 and two other SGI1-REs of *Salmonella* Heidelberg that were located at an alternative site in the intergenic region between the superoxide dismutase (*sodB*) gene and the transcriptional repressor (*purR*) gene. They were found in Enterobacteriales (*n* = 42): *Salmonella enterica* (33), *E. coli* (2), *K. pneumoniae* (2), *P. mirabilis* (2), *Cronobacter sakazakii* (1), *Morganella morganii* (1), and *Providencia stuartii* (1); Vibrionales (27): *V. cholerae* (19), *V. mimicus* (3), *Vibrio parahaemolyticus* (3), *Vibrio fluvialis* (1), and *Vibrio navarrensis* (1); Alteromonadales (15): *Shewanella* sp. (3), *Shewanella algae* (3), *Shewanella vesiculosa* (2), *Shewanella fodinae* (1), *Shewanella frigidimarina* (1), *Idiomarina tyrosinivorans* (1), *Idiomarina* sp. (1), *Marinobacter adhaerens* (1), *Marinobacter lutaoensis* (1), *Marinobacter* sp. (1); unclassified gamma-proteobacteria (2): *Pseudohongiella nitratireducens* (2); Pseudomonadales (1): *Pseudomonas aeruginosa* (1); Chromatiales (1): *Rheinheimera nanhaiensis* (1); and Oceanospirillales (1): *Halomonas meridiana* (1). The length and G+C content of all backbones ranged from 16,505 to 30,708 bp and from 42.90 to 50.51%, respectively (Supplementary Table 2).

**FIGURE 1:**
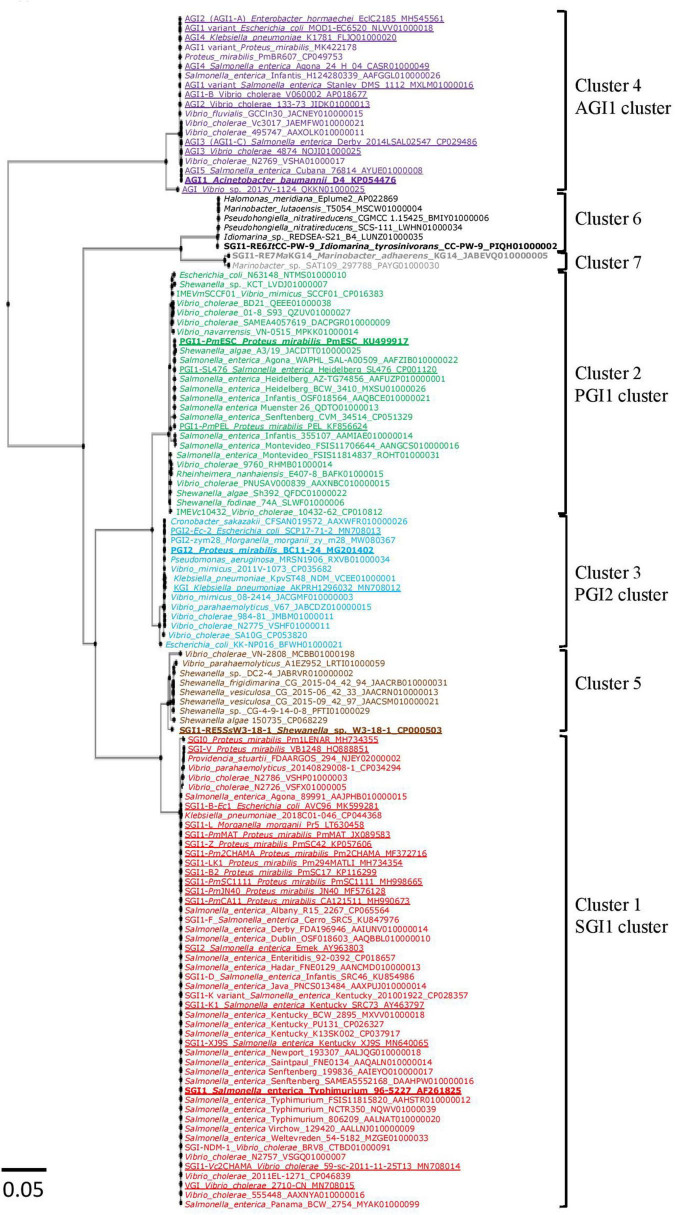
Phylogenetic tree of *int* genes of SGI1-REs included in this study. The colors of the strains correspond to the clusters of *Salmonella* genomic island 1-related elements (SGI1-REs): red (cluster1: SGI1 cluster), green (cluster 2: PGI1 cluster), blue (cluster 3: PGI2 cluster), purple (cluster 4: AGI1 cluster), brown (cluster 5), black (cluster 6), and gray (cluster 7). The characterized SGI1-REs are underlined, and the SGI1-REs used as a reference are in bold.

Phylogenetic analysis based on the nt sequences of the 126 integrase genes revealed the presence of 7 clusters. Between the members of each cluster, the *int*_GI_ gene homology was greater than 97% (Supplementary Tables 3B–G). Of the 89 new GIs retrieved, 32 SGI1-REs belonged to the SGI1 cluster (cluster 1), 23 to the PGI1 cluster (cluster 2), 11 to the PGI2 cluster (cluster 3), and 7 to the AGI1 cluster (cluster 4). The results revealed 3 additional GI clusters (Figure 1). The first cluster (cluster 5) composed of 8 GIs included SGI1-RE5*Ss*W3-18-1 from *Shewanella* sp. W3-18-1. The second cluster (cluster 6) consisting of 6 GIs included a GI found in *I. tyrosinivorans* CC-PW-9, a species isolated from marine environments, named here as SGI1-RE6*It*CC-PW-9 and used as a reference for this group since it was the only one harboring an MDR region. The last group (cluster 7) included 2 GIs from *Marinobacter* and the GI of *M. adhaerens* KG14 was named SGI1-RE7*Ma*KG14. The nt identity of the *int*_GI_ gene of the 7 SGI1-REs used as a reference is presented in Supplementary Table 3A. *Int*_PGI1_ and *int*_PGI2_ were the closest (94.98% nt identity), as were *int*_SGI1_ and *int*_SGI1–RE5_*_*Ss*_*_W3–18–1_ (94.99%). *Int*_SGI1–RE6_*_*It*_*_*CC–PW*–9_ and *int*_SGI1–RE7_*_*Ma*_*_KG14_ shared only 71–76% with *int*_*SGI*1/*PGI*1/*PGI*2_. *Int*_AGI1_ was the most distant from the other 6 *int*_GI_ (≤64.82% nt identity). The Int_GI_ integrase catalytic residue was conserved for each detected SGI1-RE (Supplementary Figure 1).

New SGI1-REs from the SGI1 cluster were detected only in Enterobacteria and in the members of the genus *Vibrio*. SGI1-REs from 23 additional *Salmonella* of various serovars [including SGI1-D and SGI1-F (Levings et al., 2005) and an SGI1-K variant (Hawkey et al., 2019)] were included in this cluster due to variations in their backbone (*int*_GI_ ≥ 99.92% nt identity). SGI1-REs belonging to the SGI1 cluster were also found in *K. pneumoniae* (1) and *P. stuartii* (1) (99.92 and 99.59% of nt identity with *int*_SGI1_, respectively). It should be noticed that among the published SGI1 variants, *int*_SGI–V_ and *int*_SGI0_ harbored by *P. mirabilis* have only 99.59 and 99.10% of nt identity with *int*_SGI1_, respectively. SGI1-REs were also recovered from 6 strains of *V. cholerae* and one strain of *V. parahaemolyticus* (*int*_GI_ ≥ 99.67% of nt identity with *int*_SGI1_) (Supplementary Table 3B).

*Salmonella* genomic island 1-related elements of the PGI1 cluster were found in more diverse bacterial species, including SGI1-REs in Enterobacteriales: *E. coli* (1) and *Salmonella* (9); in Vibrionaceae: *V. cholerae* (6), *V. mimicus* (1) and *V. navarrensis* (1); in *Shewanella*: *S. algae* (2), *S. fodinae* (1) and *Shewanella* sp. (1); and also in *R. nanhaiensis* (1), a Gram-negative rod-shaped bacterium isolated from deep-sea sediment in the South China Sea at a water depth of 1,800 m (Zhang et al., 2012). The *int*_GI_ showed ≥98.68% of nt identity with *int*_PGI1–_*_*Pm*_*_ESC_ (Supplementary Table 3C).

Eleven SGI1-REs belonged to the PGI2 cluster: SGI1-REs in *C. sakazakii* (1), *E. coli* (1), *K. pneumoniae* (1), *M. morganii* (1), in *V. cholerae* (3), *V. mimicus* (2), *V. parahaemolyticus* (1), and for the first time, a GI of the SGI1-RE family was detected in *P. aeruginosa* (1). Their *int*_GI_ showed ≥98.94% of nt identity with *int*_PGI2_ (Supplementary Table 3D).

Only 7 new SGI1-REs were found in the AGI1 cluster, a group gathering recently discovered AGI1 variants in the GenBank^®^ (Siebor and Neuwirth, 2020). They were detected in *V. cholerae* (3) and *V. fluvialis* (1). For the first time, SGI1-REs belonging to the AGI1 cluster were found in 2 strains of *P. mirabilis* (*int*_GI_ = 99.65% of nt identity with *int*_AGI1_) (Supplementary Table 3E). The SGI1-RE of *Vibrio* sp. 2017V-1124 whose *int*_GI_ shared only 96.37% of nt identity with *int*_AGI1_ was classified in this cluster (98.70–99.22% AA identity with the *Int*_*GI*_ of this cluster).

*Salmonella* genomic island 1-related elements not belonging to the SGI1/PGI1/PGI2/AGI1 clusters were identified in 16 strains isolated mainly from water or marine samples. These SGI1-REs belonged to 3 new clusters. The first cluster (cluster 5) found only in *Shewanella* and *Vibrio* species included SGI1-RE5*Ss*W3-18-1 from *Shewanella* sp., W3-18-1 and other *Shewanella* sp. (2), *S. vesiculosa* (2), *S. algae* (1), *S. frigidimarina* (1), *V. cholerae* (1), and *V. parahaemolyticus* (1). Compared to the *int*_GI_ used as a reference in each cluster, *int*_SGI1–RE5_*_*Ss*_*_W3–18–1_ and *int*_SGI1_ were the closest (94.99% nt identity), and *int*_SGI1–RE5_*_*Ss*_*_W3–18–1_ and *int*_AGI1_ were the farthest (64.34% of nt identity) (Supplementary Table 3F). The *int*_GI_ of *Shewanella* and *Vibrio* shared ≥98.28% and ≥97.54% nt identity with *int*_SGI1–RE5_*_*Ss*_*_W3–18–1_, respectively.

The second cluster (cluster 6) included 6 SGI1-REs found in *I. tyrosinivorans* (1), a Gram-negative curved rod-shaped marine bacterium isolated from the estuarine water off Pingtung in Taiwan (Hameed et al., 2016) and *Idiomarina* sp. (1); in *M. lutaoensis* (1), a marine bacterium isolated from a hot spring on the coast of Lutao in Taiwan (Shieh et al., 2003); in *H. meridiana* (1), isolated from the hydrothermal plume seawater in the Northeast Pacific Ocean at a depth of 2,000 m (Kurihara et al., 2020); and *P. nitratireducens* (2) isolated from seawater in the northern South China Sea at a 450 m water depth (Xu et al., 2016). Their *int*_GI_ showed ≥99.84% of nt identity with *int*_SGI1–RE6_*_*Id*_*_CC–PW–9_ (Supplementary Table 3G).

The last cluster (cluster 7) contained 2 SGI1-REs detected in *M. adhaerens* (1) and *Marinobacter* sp. (1) isolated from the traditional Korean soy sauce and Mediterranean Sea water, respectively; their *int*_GI_ showing only 89.52 and 89.60% of nt identity with *int*_SGI1–RE6_*_*Id*_*_CC–PW–9_, respectively. The clusters 6 and 7 were more distant from the other clusters sharing only 62–75% nt identity with *int*_AGI1_ and *int*_PGI1_, respectively (Supplementary Table 3G). We have chosen to group clusters 6 and 7 together for further analysis.

### Features of *Salmonella* Genomic Island 1-Related Elements Backbones and Phylogenetic Relationships Between Genomic Islands

**FIGURE 2:**
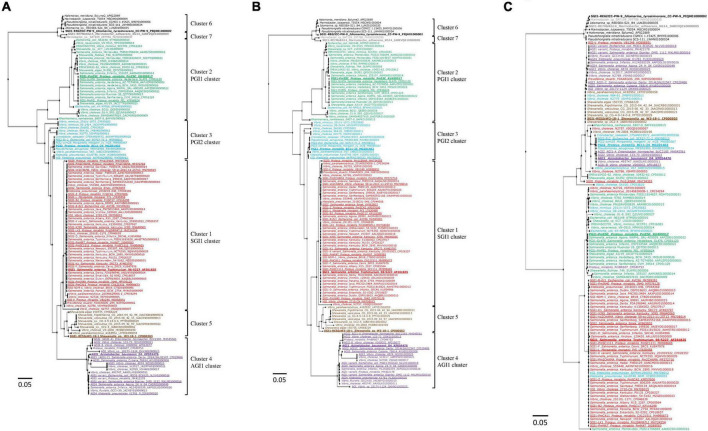
Phylogenetic tree of the backbones of SGI1-REs. **(A)** Complete backbones. **(B)** First part of the backbones (*attL-oriT* segment). **(C)** Second part of the backbones (*oriT-attR* segment). The colors of the strains correspond to the clusters of SGI1-REs: red (cluster1: SGI1 cluster), green (cluster 2: PGI1 cluster), blue (cluster 3: PGI2 cluster), purple (cluster 4: AGI1 cluster), brown (cluster 5), black (cluster 6), and gray (cluster 7). The characterized SGI1-REs are underlined, and the SGI1-REs used as a reference are in bold.

Phylogenetic analysis of the nt sequences of the complete backbones of the 126 SGI1-REs showed that the SGI1 cluster was the most homogeneous. Greater diversity was observed particularly in the PGI1 and PGI2 clusters (Figure 2A). Moreover, the SGI1-RE of *E. coli* KK-NP016 harboring *int*_PGI2_ was found in the PGI1 cluster, with a major part of its backbone sharing nt identity with PGI1 regions. The nt identity of the backbones used as a reference showed that PGI1-*Pm*ESC and SGI1-RE6*It*CC-PW-9 were the farthest from SGI1, PGI2, and AGI1 (≤58.25 and ≤53.55% nt identity) (Supplementary Table 4). These results were consistent with the branch length values shown in Figure 2A.

The backbone of the SGI1-REs could be divided into two distinct parts. The first part, from the *attL* attachment site to the *oriT* transfer origin, was highly conserved and contained *int*_GI,_ *xis* (S002), *rep* (S003), S004, *traN* (S005), *sgaC* (S006), +/−*sgaD* (S007), *traG* (S011), +/−*traH* (S012), *mpsB* (S019), and *mpsA* (S020) genes (de Curraize et al., 2021), and *oriT* known as a high potential recombination site. The second part of the backbone, from *oriT* to the 3′-end of the backbone (*attR* attachment site), varied between SGI1-REs. The phylogenetic trees obtained for the complete backbones and parts from *attL* to *oriT* were similar (Figures 2A,B). Compared to the phylogenetic analysis of the *int*_GI_ genes, the same clusters were recovered with the first parts of the backbones of SGI1-REs, except for the SGI1-RE of *E. coli* KK-NP016, which was phylogenetically closer to PGI1, despite the presence of an *int*_PGI2_ gene. The part of the backbone from *oriT* to *attR* was generally characterized by the presence of a *res* gene. When an MDR region containing a class 1 integron was present, it was inserted upstream of *res*. Phylogenetic analysis of this part of the backbone showed greater dispersion among the clusters (Figure 2C).

**FIGURE 3:**
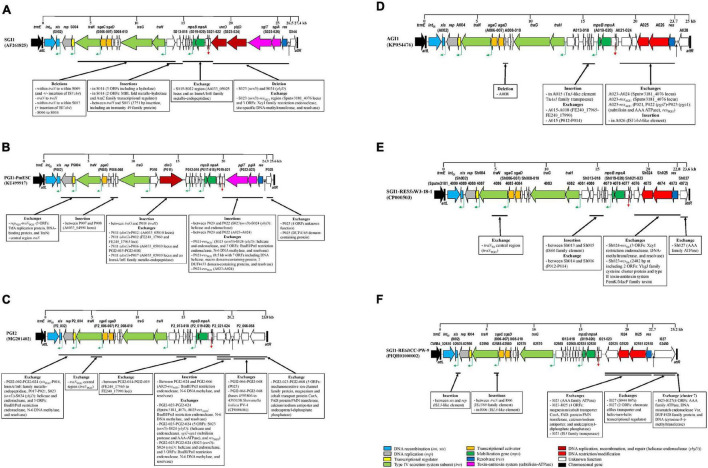
Backbone variations of SGI1-REs of each cluster compared to the SGI1-RE used as a reference. **(A)** cluster1: SGI1 cluster, **(B)** cluster 2: PGI1 cluster, **(C)** cluster 3: PGI2 cluster, **(D)** cluster 4: AGI1 cluster, **(E)** cluster 5, and **(F)** clusters 6 and 7. The original reading frames (ORFs) of the backbone used as a reference are represented. The following colors are used for the ORFs of known function: light blue, DNA recombination (*int*, *xis*); gray, DNA replication (*rep*); yellow, transcriptional regulator; light green: type IV secretion system subunit (*tra*); orange, transcriptional activator; dark green, mobilization gene (*mps*); dark blue, resolvase (*res*); pink, toxin-antitoxin system (subtilisin-ATPase); dark red, DNA replication, recombination, and repair [helicase-endonuclease (*ybjD*)]; red, DNA restriction/modification; white, unknown function; and black, chromosomal gene. The green angled arrows indicate AcaCD binding sites, the red arrow indicates *oriT*, and the black arrow indicates the MDR region. Black vertical lines represent *attL* and *attR* attachment sites.

The synthetic description of the characteristics of each cluster of SGI1-RE is reported in Figures 3A–F.

#### Features of Cluster 1 (*Salmonella* Genomic Island 1 Cluster)

Phylogenetic analysis of the first part of the backbone showed that the SGI1 cluster was the most homogeneous. BLAST alignment with SGI1 showed variations previously described in Enterobacteriales, due to deletion events in the *traN*-S009 region, and insertion of IS*Vch4* (Supplementary Figure 2A). A larger deletion extending from *traN* to *traH*, detected in *Salmonella* Hadar FNE0129 and *Salmonella* Typhimurium 806209, was not replaced by IS*Vch4*, whereas the deletion from within *traN* to within S013 was replaced by IS*Vch4* in SGI1-LK1 (de Curraize et al., 2020b). Furthermore, a large deletion (S006-S014) occurred in *V. cholerae* N2757, and the S015-S022 region with low nt identity with SGI1 contained a segment exhibiting 96.95% of nt homology with a sequence of *Aeromonas veronii* CB51 (CP015448) that included an ORF encoding an ImmA/IrrE family metallo-endopeptidase (Supplementary Figure 3A).

An insertion event occurred in the SGI1-RE of *P. stuartii* FDAARGOS_294 whose backbone shared 100% of nt identity with that of SGI-V (3 ORFs including an ORF encoding a hydrolase inserted in S014). Other insertions were detected in SGI1-REs of *V. cholerae* N2726 (2 ORFs encoding an MBL fold metallo-hydrolase and an AraC family transcriptional regulator inserted in S014) and *V. parahaemolyticus* 20140829008 (2,751 bp insertion, including an ORF encoding an immunity 49 family protein, between *traH* and S013).

The second part of the SGI1 backbone was characterized by 6 ORFs [S023 (*uvrD*), S024 (*ybjD*), S025 (*sgiT*), S026 (*sgiA*), *res*, and S044] encoding an ATP-dependent helicase, an ATP-dependent endonuclease, a type II toxin-antitoxin system (subtilisin and ATPase implicated in the maintenance of SGI1 in the bacterial cell) and a resolvase, located before the MDR region, and ORF S044 encoding a protein of unknown function located after the MDR region. These ORFs were present in most SGI1-REs of this cluster. This second part of the backbone in SGI0 only 97.19% of nt identity with SGI1. Small or large deletions occurred at the integration site of the MDR region, often due to an IS element (IS*6100* or IS*26*) present in this MDR region, leading to a truncated *res* gene, or even the loss of several ORFs downstream of *res*, and/or a truncated ORF S044 (Supplementary Figure 2A).

BLAST analysis showed that ORFs S023 and S024 were deleted in the SGI1-RE harbored by *P. stuartii* FDAARGOS_294, as for SGI-V. This part of the backbone showed only 91.40% of nt identity with SGI1. This same deletion also occurred in SGI1-REs of *V. cholerae* N2726 and *V. parahaemolyticus* 20140829008.

In the SGI1-REs of *V. cholerae* N2786 and N2757, the S023-*res* region shared the nt identity with the ORF Sputw3181_4076 of SGI1-RE5*Ss*W3-18-1, which was followed by 3 ORFs encoding an XcyI family restriction endonuclease and a site-specific DNA-methyltransferase never found in an SGI1-RE, and a resolvase that showed poor nt identity (71%) with *res*_SGI1_ (Supplementary Figure 4A). This XcyI restriction-modification system showed only 66% of nt identity with that of *Xanthomonas campestris* (M98768) and 68% of nt identity with the Cfr9I restriction-modification system of *Citrobacter freundii* (X17022) (Withers et al., 1992; Lubys et al., 1994). These last 4 SGI1-REs carried by *Vibrio* did not harbor any MDR region.

#### Features of Cluster 2 (*Proteus* Genomic Island 1 Cluster)

The backbones of the PGI1 cluster were more diverse, and the BLAST alignment showed that the complete SGI1-RE backbones of 6 *Salmonella* of various serovars, and *S. algae* A3/19, were closer to PGI1 (Figure 2A). Variations in the first part of the backbone occurred in *Vibrio* (*n* = 8/8) and *Shewanella* (3/4) species, *Salmonella* (3/10), *E. coli* N63148, and *R. nanhaiensis* E407-8 (Figure 2B and Supplementary Figure 2B). In most cases, the conjugal transfer gene *traN* showed very low nt identity with *traN*_PGI1_ (13/16), a *traH* (absent from PGI1) was found upstream of *traG* (11/16), and changes occurred in the P011-P016 region that exhibited nt homology with parts of undescribed GIs from *A. veronii* CB51 and *Aeromonas simiae* A6 (CP040449) (Supplementary Figures 3A,B). The gene (*dinG*) encoding a DEAD/DEAH box helicase (P011) was replaced by a segment including the A6033_05010 locus of *A. veronii* CB51 (13/16).

Moreover, in SGI1-REs of 3 *V. cholerae* (strains BD21, 01-8_S93 and SAMEA4057619), the *rep-traN* segment was closely related to 3 loci encoding a TrfA replication protein, a DNA-binding protein, and TraN found in *A. veronii* CB51 and *A. simiae* A6 (Supplementary Figures 3A,B). This *trfA*-like *rep* gene encoded a different protein from that encoded by *rep*_PGI1_ (pfam07042 and pfam 03090, respectively). It should be noted that the *Rep* protein of PGI1 belonged to a different family than that of SGI1, PGI2, and AGI1 (pfam 04796). For these 3 strains, the P011–P012 segment was closely related to the FE240_17960 and FE240_17965 loci of *A. simiae* A6 (Supplementary Figure 3B).

The SGI1-RE of *E. coli* KK-NP016 harboring only *int*_PGI2_ of PGI2 was composed of a large segment of PGI1, and therefore was part of this cluster. The ORF P011 was also replaced by the A6033_05010 locus of *A. veronii* CB51, and an ORF encoding an ImmA/IrrE family metallo-endopeptidase was also found in this strain, as in *Salmonella* Montevideo FSIS11814837 (Supplementary Figure 3A).

The SGI1-RE backbone of *R. nanhaiensis* E407-8 was more different. The P011-P016 region showed nt identity with the A6033_05010 locus of *A. veronii* CB51 and the PGI2-015-PGI2-022 segment of PGI2.

The second part of the PGI1 backbone consisted of 4 ORFs: P022 (*pgiT*), P023 (*pgiA*), and *res* encoding a toxin-antitoxin system (subtilisin and ATPase) and a resolvase, located before the MDR region, and the ORF P025 located after the MDR region. The *pgiT-pgiA* segment and ORF P025 shared 90.53 and 83.10% of nt identity with *sgiT-sgiA* and ORF S044, respectively (Supplementary Figure 4B). *S. algae* A3/19 carried a complete SGI1-RE (99.96% of nt identity with PGI1), with an IS*26*-disrupted *res* gene, which did not harbor any MDR region. In this cluster, most SGI1-REs found in *Vibrio* and *Shewanella* (11/12) lacked an MDR region, as did *R. nanhaiensis* E407-8, and Enterobacteriales [*E. coli* N63148, *Salmonella* Infantis 355107, *Salmonella* Montevideo FSIS11814837 and *S*. Montevideo FSIS11706644 (Supplementary Table 2)].

Variations occurred in the P022–P025 region. A segment sharing approximately 95% of nt identity with ORFs S023–S024 encoding a helicase and an endonuclease was inserted between P020 and P022 in the SGI1-RE of *R. nanhaiensis* E407-8. In other SGI1-REs, this segment was followed by 3 ORFs encoding a BsuBI/PstI restriction endonuclease and an N-6 DNA methylase sharing only 71% of nt identity with A025 and A026 of AGI1, and a resolvase which showed no significant similarity to the *res* gene of known SGI1-REs. These 5 ORFs replaced the P021-*res*_PGI1_ region in SGI1-REs from *S. fodinae* 74A, *Shewanella* sp. KCT, *E. coli* N63148 and *V. mimicus* SCCF01 (Supplementary Figures 4C,D). The right-hand side of the backbone (P025, only 86% of nt identity) was followed by 3 ORFs encoding proteins of unknown function in *E. coli* N63148. It was replaced by an ORF encoding a DUF4365 domain-containing protein in both *Shewanella* sp. KCT and *V. mimicus* SCCF01.

In the SGI1-REs from *S.* Infantis 355107, *V. navarrensis* VN-0515 and *V. cholerae* SAMEA4057619, the P021-*res* region was replaced by an 8.5 kb segment with 7 ORFs, some of which encoded a DNA helicase (no nt identity with S023 and P011), a macro domain-containing protein, 2 DUF4433 domain-containing proteins, and a resolvase (Supplementary Figure 5A).

In SGI1-REs of *S. algae* Sh392 and *V. cholerae* 10432-62, a segment sharing 96–98% of nt identity with A023 and A024 of AGI1 was inserted between P020 and P022, and in the SGI1-RE of *S.* Montevideo FSIS11706644, it replaced the P021-*res* region (Supplementary Figure 4E).

#### Features of Cluster 3 (PGI2 Cluster)

The SGI1-REs of *M. morganii* zy_m28 and *P. aeruginosa* MRSN1906 were almost identical to the PGI2 described in *P. mirabilis* (Lei et al., 2018). The central region of *traN* in SGI1-REs from *K. pneumoniae* KpvST48_NDM, *V. cholerae* N2775, and 984-81 shared nt identity with *traN* of PGI1 (Supplementary Figures 2C, 3C).

Variations were observed mainly in the PGI2-014-PGI2-019 region in *Vibrio* species. In SGI1-REs of *V. mimicus* 2011V-1073 and 08-2414, this region matched the segment of loci FE240_17965 to FE240_17990 of *A. simiae* A6, with the FE240_17970-FE240_17985 segment sharing nt identity with P013-P016 of PGI1 (Supplementary Figures 3B,C). Other parts of this region matched P012-P014 (in SGI1-REs of *V. cholerae* N2775 and 984-81) or P012-P013 (in the SGI1-RE of *V. parahaemolyticus* V67).

The SGI1-RE of *E. coli* KK-NP016 was classified as PGI2 based on the identity of its integrase gene with *int*_PGI2_. The first part of its backbone revealed that only *int*_GI_ shared an nt identity with PGI2. The segment from *xis* to *oriT* matched the corresponding region in PGI1 where P011 was replaced by the A6033_05010 locus of *A. veronii* CB51, and an ORF encoding an ImmA/IrrE family metallo-endopeptidase was found between P016 and P017 (Supplementary Figures 3A,C). These modifications confirmed that the backbone of the SGI1-RE of *E. coli* KK-NP016 was more closely related to that of PGI1, given its position in the phylogenetic tree (Figure 2A).

The second part of the PGI2 backbone did not include a *res* gene. Outside the left part of the MDR region, 2 ORFs were found (PGI2-023 and PGI2-024), the latter sharing 99.44% of nt identity with A024 of AGI1; and the right end of the backbone contained 3 ORFs (PGI2-066-PGI2-068), the first of which corresponded to the part of S044 (593/630 nt, 97.42% of nt identity) and the other two encoded a universal stress protein and a sulfate permease, respectively. The SGI1-RE of *C. sakazakii* CFSAN019572 was the only one carrying both a *res* gene and the 3 ORFs at its right end. Two additional ORFs encoding a BsuBI/PstI restriction endonuclease and an N-6 DNA methylase found between PGI2-024 and *res* shared 99.62% of nt identity with A025–A026 of AGI1. They were also found in the SGI1-RE of *V. cholerae* SA10G; and in SGI1-REs of *V. cholerae* N2775 and 984-81, they were preceded by the Sputw3181_4076 locus of *Shewanella* sp. W3-18-1 (Supplementary Figures 4A,D).

The SGI1-RE of *K. pneumoniae* KpvST48_NDM showed the same changes as the recently described KGI (Cummins et al., 2020): the PGI2-023-PGI2-024 segment was composed of 5 ORFs: 2 ORFs encoding a helicase and an endonuclease (99.78% of nt with S023–S024), 2 ORFs encoding a subtilisin protease and an AAA-ATPase (99.89% of nt identity with *sgiT-sgiA*) and a *res* gene (99.49% of nt identity with *res*_SGI1_) (Supplementary Figures 4B,C). The PGI2-066-PGI2-068 segment matched P025 (99.21% of nt identity).

In the SGI1-REs of *V. mimicus* 08-2414 and 2011V-1073, and *E. coli* KK-NP016, the PGI2-023-PGI2-024 segment also shared an nt identity (91–97%) with S023–S024 but was followed by 2 ORFs encoding a BsuBI/PstI restriction endonuclease and an N-6 DNA methylase that showed a low nt identity (71%) with A025–A026 of AGI1 and a *res* gene (72% of nt identity with *res*_*AGI*1_) (Supplementary Figures 4C,D). These 3 ORFs showed greater than 98% of nt identity with those found in the SGI1-RE of *V. mimicus* SCCF01 that belonged to the PGI1 cluster (Supplementary Figure 5B). For most *Vibrio*, the right end of the backbone shared nt identity with P025 (>98.57%), whereas it matched the *Shewanella loihica* PV-4 (CP000606) nt sequence from bases 4593806 to 4593138 (98.65% of nt identity) in *E. coli* KK-NP016.

The second part of the SGI1-RE of *V. parahaemolyticus* V67 was unusual. The PGI2-023-PGI2-068 region corresponded to 5 ORFs encoding a mechanosensitive ion channel family protein, a magnesium and cobalt transport protein CorA, a FAD: protein FMN transferase, a calcium/sodium antiporter, and an undecaprenyl-diphosphate phosphatase. This part had neither a *res* gene nor an MDR region.

#### Features of Cluster 4 (*Acinetobacter* Genomic Island 1 Cluster)

This cluster was relatively homogeneous. An SGI1-RE belonging to the AGI1 cluster was detected for the first time among *P. mirabilis* (2 strains). Previously described minor deletions occurred (loss of A008 or shorter intergenic region between A018 and A019, Supplementary Figure 2D). Moreover, a Tn*3*-like element was inserted into the A015 of the SGI1-RE of *V. cholerae* 495747.

The A014–A018 region of the SGI1-REs of *V. cholerae* Vc3017 and N2769 shared nt identity with the FE240_17965-FE240_17990 segment of *A. simiae* A6, as described for SGI1-REs of *V. cholerae* 4874 and *V. mimicus* 2011V-1073 belonging to the PGI2 cluster (Supplementary Figure 3B).

The second part of the AGI1 backbone consisted of 5 ORFs (A023–A024, A025–A026 encoding a BsuB1-Pst1 restriction endonuclease and an N-6 DNA methylase, and res_AGI1_) located before the MDR region, and the ORF A028 located after the MDR region. The main changes were the deletion of the A023–A024 region and replacement by a segment sharing nt identity with the Sputw3181_4076 locus of SGI1-RE5*Ss*W3-18-1 (98.76%) or deletion of A023-*res* as previously described (Siebor and Neuwirth, 2020) (Supplementary Figure 4A). Moreover, the ORF A026 encoding an N-6 DNA methylase was disrupted by an IS*Vch4*-like element in the SGI1-RE of *V. cholerae* N2769.

In the SGI1-RE of *P. mirabilis* PmBR607, the A023-*res* region shared an nt identity with P022-*res* (99.98%), which included the toxin-antitoxin system composed of ORFs encoding a subtilisin protease and an AAA-ATPase (Supplementary Figure 4B).

#### Features of Cluster 5

*Salmonella* genomic island 1-related elements of this cluster were only found in *Shewanella* and *Vibrio* species. Most of the backbone of SGI1-RE5*Ss*W3-18-1 (ORFs named here Sh001-Sh027) was almost identical to that of SGI1 (*int*_SGI1_-ORF S022 and ORF S044, 95 and 90% of nt identity, respectively) and AGI1 (*rep-*A022 and A025-*res*, 96 and 99% of nt identity, respectively) (Siebor and Neuwirth, 2020). The SGI1-RE of *S. frigidimarina* CG_2015-04_42_94 exhibited 94.52% of nt identity with SGI1-RE5*Ss*W3-18-1 (Figure 2A and Supplementary Figure 2E). There were few variations in this first part of the backbone. In the SGI1-RE of *Shewanella* sp. DC2-4, the region between Sh014 and Sh016 shared nt identity with P012-P014 (Supplementary Figure 3C). More variations occurred in the SGI1-RE of *V. cholerae* VN-2808 corresponding to the insertion of mobile elements: transposases of the IS*4*, IS*5*, and IS*66* families between *xis* and *rep*, in Sh009 and between Sh013 and Sh015, respectively. Furthermore, the central region of *traN* shared nt identity with *traN* of PGI1 (Supplementary Figure 3C).

The second part of the backbone consisted of 5 ORFs: the Sputw3181_4076 locus (ORF Sh023) also found in some AGI1 variants (Siebor et al., 2019), the other two ORFs (Sh024–Sh025) encoded a restriction endonuclease belonging to the BsuB1*-*Pst1 family protein and a restriction-modification methylase related to Eco57I, as in AGI1 (99.37% of nt identity) (Supplementary Figure 4D), a *res* gene and the Sputw3181_4072 locus (Sh027, 92.53% of nt identity with S044). Sh027 was present in all SGI1-REs of this cluster, except in the SGI1-RE of *Shewanella* sp. DC2-4 where an ORF encoding an AAA family ATPase was found. No SGI1-REs in this cluster carried an MDR region.

Variations occurred in the Sh024-*res* region. In the SGI1-RE of *V. parahaemolyticus* A1EZ952, the ORFs Sh024-Sh025 encoded a restriction endonuclease belonging to the XcyI family and a site-specific DNA-methyltransferase (99.36 and 98.24% of nt identity with those of SGI1-REs from *V. cholerae* N2786 and N2757 in the SGI1 cluster, respectively), and the adjacent *res* gene (99.66 and 97.47% of nt identity, respectively) was only 71% identical to *res*_SGI1–RE5*Ss*W3–18–1_ (Supplementary Figure 5C).

In the SGI1-RE of *V. cholerae* VN-2808, the Sh023-*res* region was replaced by a 2,482 bp nt sequence which exhibited no significant homology with sequences deposited in the NCBI database. The BLASTX alignment identified 2 ORFs encoding a YkgJ family cysteine cluster protein and a type II toxin-antitoxin system PemK/MazF family toxin. The *res* gene was absent. The ORF Sh027 at the 3′-end of the backbone showed only 92.53% of nt identity with that of SGI1-RE5*Ss*W3-18-1.

#### Features of Clusters 6 and 7

The backbone of SGI1-RE6*It*CC-PW-9 was composed of 27 ORFs named here I001-I027. The gene synteny of its first part (I001–I022) was highly conserved compared to SGI1. Indeed, it contained genes and ORFs whose products exhibited significant homology with known protein families, in particular, a recombination module (Int and Xis), a replicase, a transcriptional regulator, proteins involved in conjugation (TraN, and TraG) and mobilization (MpsB/MpsA), and a SgaCD-like transcriptional activator (Supplementary Figure 2F).

In the SGI1-REs of cluster 6, some variations were linked to the insertion of IS elements. An insertion sequence of the IS*1380* family was found between *traN* and ORF I006 in the SGI1-RE of *H. meridiana* Eplume2. An IS*L3*-like element was inserted between *xis* and *rep* and in I006 in SGI1-REs from *P. nitratireducens* SCS-111 and CGMCC 1.15425. The I013–I014 region in the SGI1-REs of both *P. nitratireducens* showed low similarity to P013. Regarding cluster 7, an IS*66* family transposase was inserted between *xis* and *rep* in the SGI1-RE of *M. adhaerens* KG14.

The second part of the SGI1-RE6*It*CC-PW-9 backbone consisted of 5 ORFs: ORFs encoding a radical SAM family maturase, a BsuB1-Pst1 restriction endonuclease and an N-6 DNA methylase sharing only 71% of nt identity with A025-A026 (Supplementary Figure 4D), and a resolvase that were located before the MDR region; and the right end of the backbone contained an ORF (I027) encoding an AAA family ATPase (97.28% of nt identity with that found in the SGI1-RE of *Shewanella* sp. DC2-4 from cluster 5). Only SGI1-RE6*It*CC-PW-9 harbored an MDR region.

Changes occurred in the I023–I025 region. In the SGI1-REs of cluster 6 (from *H. meridiana* Eplume2, *P. nitratireducens* SCS-111, and CGMCC 1.15425), I023 corresponded to an ORF encoding an AAA family ATPase exhibiting no significant similarity with the ORF I027 at the right end of SGI1-RE6*It*CC-PW-9. Furthermore, ORF I027 shared 86% of nt identity with S044.

Other changes were found in the SGI1-RE of *M. lutaoensis* T5054: the I023–I025 segment was replaced by 4 ORFs encoding a magnesium/cobalt transporter CorA, a FAD: protein FMN transferase, a calcium/sodium antiporter, and an undecaprenyl-diphosphate phosphatase. In the SGI1-RE of *Idiomarina* sp. REDSEA-S21_B4, the ORF I023 matched an IS*3* family transposase, and I027 corresponded to 2 ORFs encoding a chromate efflux transporter and a helix-turn-helix transcriptional regulator.

In the SGI1-REs of cluster 7 (*M. adhaerens* KG14 and *Marinobacter* sp. SAT109), the I023–I027 region corresponded to 4 ORFs encoding an AAA family ATPase, a DNA mismatch endonuclease Vsr, a DUF4928 family protein, and a DNA (cytosine-5-)-methyltransferase. The *res* gene was absent.

#### G + C Content of *Salmonella* Genomic Island 1-Related Elements

The G + C contents of SGI1-RE backbones ranged from 42.90 to 44.97% for the SGI1, PGI2, AGI1 clusters, and cluster 5, except for the SGI1-RE of *E. coli* KK-NP016, whose backbone was mainly composed of a PGI1 element, from 46.61 to 49.46% in the PGI1 cluster, and from 47.83 to 50.51% in clusters 6 and 7 (Supplementary Table 2). On comparison, it was found that the median G + C content of host genomes ranged from 38.8% in *P. mirabilis* to 66.5% in *P. aeruginosa*. It is noteworthy that the G + C content of *Vibrio* and *Shewanella* genomes (45.3–50% and 41.35–53%, respectively) was similar to that of SGI1-REs (Supplementary Table 5).

### Boundaries of *Salmonella* Genomic Island 1-Related Elements

All SGI1-REs were integrated at the 3′-end of *trmE*, except for the 3 PGI1 variants of *S.* Heidelberg that were inserted into the intergenic region between the *sodB* and *purR* genes. The 18-bp attachment sites (*attL* and *attR*) were identified (Supplementary Table 2). Based on the analysis of the *att* sites after conjugation experiments, the last 9-bp of *att* sites were examined (Siebor and Neuwirth, 2020). As expected, the last 9-bp of *attR* sites corresponding to the 3′-end of the *trmE* gene were variable, as *trmE* genes are quite different among the bacterial species (Doublet et al., 2007) (Supplementary Figure 6). Only two different sequences were detected for the last 9-bp of *attL* sites corresponding to the *attP* site of the circular form of SGI1-REs. The 9-bp GGGAAGTAA sequence was found in all SGI1-REs of SGI1 cluster, and GGGAAGTGA in SGI1-REs of the other clusters (PGI1, PGI2, AGI1 clusters, and clusters 5, 6, and 7), except for SGI1-REs of *V. mimicus* 2011V-1073 and 08-2414 from PGI2 cluster, and that of *M. adhaerens* KG14 from cluster 7, which were the same as for SGI1 cluster.

### Coexistence in the Tandem Arrays of Genomic Islands Elements

Complex GIs consisted of two elements sharing the same attachment site and integrated in tandem array. GI tandems were found in 2 strains, in addition to the one previously described in *P. mirabilis* JN40. In each case, a classical SGI1-RE (from different cluster) was inserted at the 3′-end of *trmE* and was followed by a GI of other families that targeted the same attachment site. The GI in tandem (23,741 bp) with SGI1-*Pm*JN40 from *P. mirabilis* JN40 showed 100% of nt identity with GIs found at the 3′-end of *trmE* in *P. mirabilis* strains isolated from wild animal samples (CP073245, CP053681, CP053682, and CP053614). It harbored 17 ORFs and contained genes encoding an integrase, DNA binding proteins (including an excisionase family protein), transcriptional regulators belonging to the AlpA family phage regulatory protein and the XRE family transcriptional regulator, a toxin-antitoxin module, and a DEAD/DEAH box helicase (Bie et al., 2018). The *int*_GI_ gene showed 89% of nt identity with that of *V. parahaemolyticus* strains (CP012950 and CP034305) whose GIs were also integrated at the 3′-end of *trmE*. The last 9-bp of the *attL* site were identical to those of the PGI1 cluster.

A GI not belonging to the SGI1-RE family was located at the right end of SGI1-REs in *V. cholerae* 555448 of the SGI1 cluster, and in *V. cholerae* N2775 of the PGI2 cluster. The former (19,970 bp) harbored 17 ORFs including genes encoding an integrase, a DNA-binding protein (excisionase family), transcriptional regulators (AlpA and XRE families), a methyltransferase, a cysteine desulfurase, and a PAPS sulfotransferase. The backbone shared approximatively 90% of nt identity (84% coverage) with the GIs of *V. parahaemolyticus* strains (CP034305 and CP051111) also located at the 3′-end of *trmE.* However, the *int*_GI_ gene was only 93.25% identical to that of *V. parahaemolyticus* 2012V-1165 (CP051111).

The GI in tandem *V. cholerae* N2775 (17,347 bp) harbored 15 ORFs and was closer to that found in *S. algae* 2NE11 (CP055159) at the 3′-end of *trmE* (96.98% of nt identity with 89% coverage). It carried genes encoding an integrase, a DNA-binding protein (excisionase family), a helicase, transcriptional regulators (AlpA and XRE families), a virulence RhuM family protein, a restriction endonuclease, N-6 DNA methylases, and a resolvase. Its *int*_GI_ gene showed 99.91, 94.96, and 94.19% of nt identity with the *int*_GI_ of a GI found downstream of *trmE* in *V. cholerae* FORC_073 (CP024082), *S. algae* 2NE11 (CP055159), and GIPmi1 (MF490433), respectively. We noticed that for both GIs in the tandem of *V. cholerae*, the last 9-bp of the *attL* site was identical to those of the SGI1 cluster.

### Boundaries of the Multidrug Resistance Regions

As usual, MDR regions were inserted upstream of the *res* gene and generated a 5-bp target site duplication. In some cases, insertion of the MDR region resulted in larger or smaller deletions of *res*, or on the 3′-side of the backbone, or on both sides. The insertion site was then determined on the intact side when present. Of the 89 uncharacterized SGI1-REs, 41 of them lacked an MDR region. These MDR-free SGI1-REs were detected mainly in bacteria not belonging to the Enterobacteriales, particularly *Vibrio* (*n* = 18), *Shewanella* (9), and marine bacteria (8): *Rheinheimera*, *Halomonas, Idiomarina, Marinobacter*, and *Pseudohongiella*. Furthermore, 4 of them (belonging to the PGI2 cluster and to clusters 5 and 7) did not harbor a *res* gene (Supplementary Table 2).

Except in the case of SGI2 for which the MDR region was not inserted upstream of the *res* gene, but in S023, the MDR regions of SGI1-REs of the SGI1 cluster were inserted at the *res* site in 3 different positions (de Curraize et al., 2021). Most of them were at the ACTTG site. The backbones of SGI1-*Pm*2CHAMA and VGI-*Vc*2CHAMA were identical and their MDR region was inserted at position AACTT, as for the SGI1-RE of *Salmonella* Saintpaul FNE0134. This position was shifted one bp to the left relative to ACTTG. For SGI-V and SGI1-RE from *P. stuartii* FDAARGOS_294, whose backbone was identical to that of SGI-V, their MDR region was inserted in another position (AAATT). It is noteworthy that this position was also observed for the insertion of the MDR region into PGI1. Indeed, *res*_SGI–V_ showed only 97.26% of nt identity with *res*_SGI1_, but 99.32% of nt identity with *res*_PGI1_. As with SGI1-XJ9S, the insertion site of the MDR region of 7 SGI1-REs could not be determined due to the deletion of parts of *res* and S044 after insertion of the MDR region flanked by IS elements. Finally, in addition to SGI0 and VGI, 4 SGI1-REs from *Vibrio* did not contain an MDR region.

Regarding the PGI1 cluster, the position of the MDR region was only detected at a single position (AAATT) for the published SGI1-REs. Only the insertion site of the MDR region of the SGI1-RE from *S. fodinae* 74A (ATGAC) was different from that of PGI1. Indeed, the P022-*res* region and P025 located on either side of the MDR region were different, including the genes encoding a BsuBI/PstI restriction endonuclease and an N-6 DNA methylase downstream of *res* (Supplementary Figures 2B, 5B). Its *res* gene shared from 99.00 to 99.34% of nt identity with *res* from 6 other SGI1-REs (3 from PGI1 clusters and 3 from PGI2 cluster) that also harbored these same ORFs downstream of *res*, but they were free from MDR region. Of the SGI1-REs recovered, 16/23 did not harbor an MDR region; among them were *E. coli* (*n* = 1), *Salmonella* (3), but mostly *Vibrio* (8), *Shewanella* (3), and *R. nanhaiensis* E407-8.

The insertion site of the MDR region in PGI2 could not be determined due to the absence of the *res* gene and a truncated S044-like element at the right end of the backbone. In contrast to PGI2, a *res* gene and an MDR region were present in SGI1-REs of *C. sakazakii* CFSAN019572, *V. cholerae* 984-81, and N2775. This *res* gene showed 99.66–99.83% of nt identity with *res*_AGI1_. The insertion sites of MDR region for these SGI1-REs were determined (CATAG) and were the same as some insertion sites of the MDR region in the AGI1 cluster. This insertion site was previously found for the AGI1 variants of *S. enterica* Derby 2014LSAL02547 and *V. cholerae* 4874. As for KGI, 6/11 new SGI1-REs were exempt from MDR region, among them were *E. coli* (*n* = 1), *K. pneumoniae* (1), and *Vibrio* (4). Moreover, the SGI1-RE of *V. parahaemolyticus* V67, which had a unique PGI2-023-PGI2-068 region, did not harbor a *res* gene.

Five insertion sites of the MDR region were previously described (ATAGG, CATAG, CCATA, TAGGT, and TGCAC) for SGI1-REs of the AGI1 cluster (Siebor and Neuwirth, 2020). The ATAGG position found in AGI1 was the most common. The other positions were shifted 1-bp, 2-bp to the left and 1-bp, 32-bp to the right relative to ATAGG, respectively. Moreover, a particular situation was observed for the SGI1-RE of *P. mirabilis* PmBR607, which was characterized by the absence of a target site duplication generated by the insertion of the MDR region. This feature was a consequence of the backbone composition. Indeed, the *res* gene showed 100% of nt identity with resPGI1 and the 5-bp near the 5′-end of the class 1 integron were AAATT, as for PGI1; at the right end of the backbone was A028 (100% nt identity) and the 5-bp near the 3′-end of the integron were ATAGG, as for AGI1. Finally, the MDR region of the SGI1-RE from *V. cholerae* 495747 made of a Tn*3*-like element was inserted into A015 and created the duplication of the ATCGT site.

In clusters 5 and 7, none of the SGI1-REs carried an MDR region. It should be noted that 3 SGI1-REs among them did not harbor a *res* gene (*V. cholerae* VN-2808 from cluster 5, *M. adhaerens* KG14, and *Marinobacter* sp. SAT109 from cluster 7). In cluster 6, only the SGI1-RE from *I. tyrosinivorans* CC-PW-9 carried an MDR region. Its *res* gene showed 97.61% of nt identity with the *res* of SGI1-REs without MDR region from *H. meridiana* Eplume2 and *P. nitratireducens*. Insertion of the MDR region generated a 5-bp duplication site (AAATG).

### Resistance Genes Associated With *Salmonella* Genomic Island 1-Related Elements

Only SGI1-REs with a complete MDR region obtained on a single contig caught our attention. In most cases, the MDR regions contained a class 1 integron that was inserted by a transposition event upstream of the *res* gene, resulting in a duplication of the target site. Only genes conferring resistance to the main antibiotics used in the clinical practice or a particular resistance that has never been described in an MDR region will be presented.

*Salmonella* genomic island 1-related elements contributed to the diffusion of multidrug resistance genes, including extended spectrum beta-lactamase (ESBL) or carbapenemase genes, sometimes associated with 16S rRNA methyltransferase genes or quinolone resistance genes. Coexistence of the *bla*_VEB–6_ ESBL gene and the *qnrA1* quinolone resistance gene was found in the MDR region of the SGI1-RE harbored by *P. stuartii* FDAARGOS_294, as previously described in SGI-V (Siebor and Neuwirth, 2011).

In the MDR region of the AGI1 variant of *P. mirabilis* PmBR607, the class 1 integron was followed by an ISCR1 element and the *bla*_CTXM–2_ ESBL gene. The MDR region of the recently described PGI2 variant of *M. morganii* zy_m28 (Xiang et al., 2021) contained numerous copies of IS*26* elements and antibiotic resistance genes (ARGs), including the *bla*_CTX–M–3_ ESBL gene, the fosfomycin resistance gene *fosA3*, and the fluoroquinolone acetylating aminoglycoside-(6′)-*N*-acetyltransferase gene *aac*(6′)-Ib-cr, as already described in PGI2-C55 (Lei et al., 2020b).

The carbapenemase gene, *bla*_*NDM–1*_ and the 16S rRNA methyltransferase gene, *armA* conferring resistance to most beta-lactams, including carbapenems, and most aminoglycosides, respectively, were present in the SGI-NDM-1 of *V. cholerae* BRV8. The NDM-1 carbapenemase-encoding gene was already described in PGI1-*Pm*PEL (Girlich et al., 2015), while the ArmA-encoding gene had never been encountered on an SGI1-RE until now. In contrast, another 16S rRNA methyltransferase gene (*rmtC*) has been detected in SGI1-S (Wilson and Hall, 2010).

The MDR region of the PGI1 variant from *S. fodinae* 74A was a Tn*3* family transposon (91% of nt identity with Tn*5058*: Y17897) that harbored genes encoding MgtC/SapB and MgtA proteins involved in magnesium transport and two other hypothetical proteins of unknown function. It shared 87.61% of nt identity with a transposon from *Citrobacter* sp. Y3 (CP050009). Genes encoded by *mgtABC* have been described in *Salmonella* pathogenicity island 3 (SPI-3) (Fàbrega and Vila, 2013). They are involved in intracellular survival. To our knowledge, the mgtCB operon has never been found on a transposon.

The SGI1-RE of *I. tyrosinivorans* CC-PW-9 was the only one in cluster 6 to harbor an MDR region that contained a Tn*402*-like class 1 integron carrying *cmlA1* and *aadA2*. It was acquired *via* a transposition event as shown by the detection of direct repeats.

### Identification of AcaCD Binding Sites

Using the motif sequence of AcaCD binding sites, five target sites were detected upstream of *xis* (S002), S004, *traN* (S005), *traH* (S012), and S018 on SGI1, and homologous genes on PGI2, AGI1, and SGI1-RE5*Ss*W3-18-1 (Figures 3A,C–E, Supplementary Figure 7, and Supplementary Table 6). In SGI1-RE6*It*CC-PW-9, the target site upstream of *traG* (I011) was detected in place of that of *traH*, which was absent in this SGI1-RE (Figure 3F). In PGI1, six target sites were identified upstream of *xis* (P002), PS004, *traN* (P004), *traG* (P009), P012, and P016 (Figure 3B).

In the SGI1-REs of the SGI1, PGI2, AGI1 clusters, and cluster 5, the sequences of AcaCD sites upstream of each gene varied slightly among SGI1-REs (de Curraize et al., 2021). However, the sequences of AcaCD_PGI1_, AcaCD_SGI1–RE6_*_*It*_*_CC–PW–9_, and AcaCD_SGI1–RE7_*_*Ma*_*_KG14_ sites were more different from AcaCD_SGI1_, AcaCD_PGI2_, AcaCD_*AGI*1_, and AcaCD_SGI1–RE5_*_*Ss*_*_W3–18–1_ sites, for each gene. Furthermore, variations in the number of sites were detected in some SGI1-REs. This was a consequence of the changes found in the backbone sequence. The AcaCD_SGI1_ site upstream of *traN* lacked in the SGI1-HKL subgroup (de Curraize et al., 2020b). In the PGI1 cluster, an AcaCD site was found upstream of either *traG* or *traH*, depending on the *tra* genes present. In SGI1-REs of *V. cholerae* BD21, 01-8_S93 and SAMEA4057619 (from the PGI1 cluster), for backbone parts sharing homology with *A. simiae* A6, AcaCD sites upstream of *xis* and PS004 were closer to the sites of AcaCD_PGI2_, AcaCD_SGI1_, or AcaCD_AGI1_. Likewise, for parts of the backbone sharing homology with PGI1 in the PGI2 and AGI1 cluster, the AcaCD sites were similar to those of AcaCD_PGI1_.

## Discussion

The screening of SGI1-REs in the WGS database had made possible the detection of GIs whose backbone presented the same gene synteny as these elements. SGI1-REs were mainly found in the bacterial species frequently isolated not only in the human and animal samples (Enterobacteriales and *P. aeruginosa*), but also in the water samples (*V. cholerae*) and belonged to the 4 groups of SGI1-REs already described. Most SGI1-REs in Enterobacteriales carried an MDR region (36/42), but only 8/19 in *V. cholerae*. This would certainly be due to the acquisition of resistance genes following exposure to antibiotics in Enterobacteriales which constitute the intestinal flora, whereas *V. cholerae* is a bacterium of the water environment. SGI1-REs were also found in the bacterial species isolated from crustaceans and marine molluscs, and more surprisingly from groundwater and seawater at great depths (*V. cholerae*, *V. parahaemolyticus*, *V. mimicus*, *Shewanella*, *R. nanhaiensis*, *H. meridiana*, *Idiomarina*, *Marinobacter*, and *Pseudohongiella*). Based on their *int*_GI_ nt identity, the latter belonged mainly to the PGI1 cluster and the 3 new clusters 5, 6, and 7 described in this article. Only 3/22 of these SGI1-REs carried an MDR region. These observations suggested that environmental waterborne bacterial species could serve as a reservoir for GIs. These MDR-free GIs might be the forerunners of the MDR-harboring SGI1-REs, as supposed for the MDR-free VGI and SGI1 (Cummins et al., 2020). They could evolve thanks to the following genetic events (insertion, deletion, and homologous recombination), acquire ARGs, and be selected under antibiotic pressure.

Phylogeny of SGI1-REs based on the nt sequence of their integrase showed that the SGI1 cluster and cluster 5 on the one hand, the PGI1 and PGI2 clusters, on the other hand, were close. The construction of a phylogenetic tree of SGI1-REs based on the nt sequence of their complete backbone reflected variations in the backbone composition. The backbones in the SGI1 cluster were very close, while the greatest diversity was observed in the PGI1 cluster. The SGI1, AGI1, PGI2 clusters, and cluster 5 were quite close, which was also corroborated by their G + C content (43–45%). The PGI1 cluster, and clusters 6 and 7 were further apart (G + C content 46.5–48.5% and 48–50.5%, respectively). The backbone of GIs is generally characterized by a different G + C content than the genome. However, among the bacterial host species, *Vibrio* and *Shewanella* had a G + C% of 45.3–47.5 and 44.05–49, respectively, suggesting a probable origin from these species. Base composition and phylogeny analysis suggested that SGI1-REs of the PGI1 cluster and clusters 6 and 7 would originate from a different ancestor than those of the other clusters.

*Salmonella* genomic island 1-related elements from SGI1/PGI1/PGI2/AGI1 clusters and new clusters 5, 6, and 7 were all integrated at the 3′-end of *trmE*, except the PGI1 variants of *S.* Heidelberg which were integrated into a secondary attachment site located in the intergenic region between the *sodB* and *purR* chromosomal genes. Possible integration at this site has been already demonstrated by conjugation experiments with a deleted *trmE* gene (Doublet et al., 2008). The 3′-end of *trmE* is a known hot spot for GI integration, as several types of GIs are integrated at this site: GI*Pmi*1 (Siebor et al., 2018), GI*Pvu*1 (MK599199), and GI*Pvu*2 (MK599200). SGI1-REs were able to be arranged in tandem (Doublet et al., 2008). A similar configuration was observed in *V. cholerae* 555448 and N2775 in which an SGI1 or PGI2 element and an uncharacterized GI were in tandem. A similar tandem array with GIs of different families was previously detected in *P. mirabilis* JN40 in which SGI1 was associated with an uncharacterized GI (Bie et al., 2018). GIs from different families could be arranged in a tandem array since they target the same bacterial *attB* site. Indeed, the latter GI was found at the 3′-end of *trmE* from *P. mirabilis* MPE0108 (CP053614) (100% nt identity) isolated from zoo animals. The formation of a tandem array of GIs could contribute to their spread and persistence with or without antibiotic selection pressure as previously suggested (Doublet et al., 2008).

**FIGURE 4:**
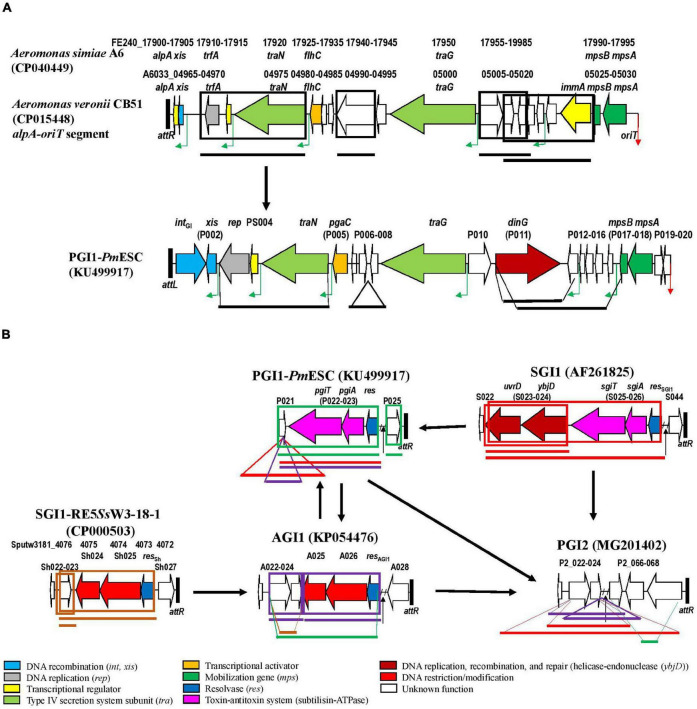
Evolution of SGI1-REs by the insertion or exchange of parts of genetic elements from another GI family or from an SGI1-RE of another cluster. **(A)** In the first part of the SGI1-RE backbone. **(B)** In the second part of the SGI1-RE backbone. The ORFs of the backbone used as a reference are represented. The following colors are used for the ORFs of known function: light blue, DNA recombination (*int*, *xis*); gray, DNA replication (*rep*); yellow, transcriptional regulator; light green: type IV secretion system subunit (*tra*); orange, transcriptional activator; dark green, mobilization gene (*mps*); dark blue, resolvase (*res*); pink, toxin-antitoxin system (subtilisin-ATPase); dark red, DNA replication, recombination, and repair (helicase-endonuclease (*ybjD*); red, DNA restriction/modification; and white, unknown function. The green angled arrows indicate AcaCD binding sites, and the red arrow indicates origin of transfer (*oriT*). Black vertical lines represent *attL* and *attR* attachment sites. Regions of the GI that have been relocated are framed: black for *yicC*-targeting GI from *Aeromonas*, red for SGI1, green for PGI1, blue for PGI2, purple for AGI1, and brown for SGI1-RE5*Ss*W3-18-1. Positions in the recipient GI are indicated by lines of the same size and color than in the donor.

Our study detected numerous mosaic structures and demonstrated that genetic exchanges by homologous recombination could occur between SGI1-REs (SGI1, PGI1, PGI2, and AGI1) and/or GIs of other types. In several SGI1-REs, segments of backbone matched with the elements from *Vibrio*, *Shewanella*, or *Aeromonas*. It should be noticed that the loci of *A. veronii* CB51 and *A. simiae* A6 sharing nt identity with the backbone segments were part of a GI integrated at the 3′-end of a gene encoding a putative stress-induced protein named *yicC*. These observations indicated that genetic exchange might also occur with GIs from other families and therefore play a key role in the evolution of SGI1-REs. A possible evolutionary history of these elements is reported in Figures 4A,B.

In each cluster, the first part of the backbone (from the *int* gene to the mobilization region carrying the *mpsA* and *mpsB* genes encoding proteins for the conjugative transfer of the SGI1-RE) was relatively well conserved in each SGI1-RE despite regions of low nt identity. For many SGI1-REs, significant exchanges occurred in the vicinity of *mpsB-mpsA*. Moreover, the *oriT* is located next to these two mobilization genes (Kiss et al., 2019). This highlights that *oriT* could be a potential site of recombination. This was observed notably for SGI1-REs of some *Vibrio* where the region next to *oriT* was different from that of the SGI1-RE used as a reference, as shown by the dispersion of some *oriT-attR* segments outside their cluster in Figure 2C. It should be noted that most of these SGI1-REs from *Vibrio* did not carry an MDR region.

Most pathogenic bacteria-harboring an SGI1-RE carried an MDR region that was not always inserted at the same site, mainly among SGI1-REs of the SGI1 and AGI1 clusters. This suggested that they did not belong to the same lineage and were not derived from a single ancestor. The unusual situation of the MDR region of *P. mirabilis* PmBR607 (different site at each side) probably resulted from genetic rearrangements in the backbone. The absence of the *res* gene in PGI2 could be the consequence of the insertion of the MDR region followed by the loss of part of the backbone on both sides of the MDR region. The left hand of the PGI2 backbone shared 99.97% of nt identity with the SGI1-RE of *C. sakazakii* CFSAN019572 harboring a *res* gene. Thus, the missing part in PGI2 could be a segment with genes encoding a BsuBI/PstI restriction endonuclease, a DNA methylase and a resolvase, as in *C. sakazakii.* Some SGI1-REs harbored several ARGs, such as ESBLs, carbapenemases, 16S rRNA methyltransferases, and fluoroquinolone resistance genes. The acquisition of these ARGs has led to the generation of superbugs that are resistant to last-resort antibiotics. Among the 17 SGI1-REs in clusters 5, 6, and 7, only one SGI1-RE harbored a class 1 integron acquired by transposition. It is noteworthy that only 4 SGI1-REs (*V. parahaemolyticus* V67 from PGI2 cluster, *V. cholerae* VN-2808 from cluster 5, and *M. adhaerens* KG14 and *Marinobacter* sp. SAT109 from cluster 7) did not carry a *res* gene. Therefore, this compromises the acquisition of an integron.

Dissemination of many GIs occurs by bacterial conjugation. The SGI1-REs of the SGI1/PGI1/PGI2/AGI1 clusters are integrative and mobilizable elements. They utilize the transfer machinery encoded by the IncA and IncC plasmids, but also the plasmid-encoded master regulator AcaCD to induce their excision and conjugation (Kiss et al., 2015). In this article, the AcaCD sequences were predicted by FIMO based on the AcaCD logo presented by Carraro et al. (2014). As well as *oriT*, mobilization genes and AcaCD binding sites were present in the SGI1-REs of clusters 5, 6, and 7, their conjugative transfer could occur with helper plasmids. Therefore, all the SGI1-REs characterized in this work might be transferred.

A more relevant classification of SGI1-REs is therefore needed. We propose that nomenclature is based on integrase gene similarity to classify SGI1-REs into different clusters, as specified for other genetic elements, such as integrons, to clarify the *int*_GI_ lineage. At present, there are seven clusters described in this article. New SGI1-REs should be assigned to an existing cluster according to the homology of their *int*_GI_ gene (nt homology > 97%, corresponding to AA homology > 98%) or within a new cluster when their *int*_GI_ gene does not reach this degree of homology. Since SGI1-REs are not genus-specific, they should be named, SGI1-RE followed by the cluster number (1–7) to which they belong, the bacterial species in which they were first described, and the backbone-specific number. SGI1-REs with the same modifications as published in another bacterial species should have the same name to be consistent with them and allow for the comparison of SGI-REs. For example, the SGI1-RE of *V. cholerae* 2011EL-1271 (<0.2% of nt substitutions relative to SGI1) was named SGI1-RE1-*S*Ty1; whereas the SGI1-RE of *V. parahaemolyticus* 20140829008-1 with new backbone modifications was called SGI1-RE1-*Vpa*14. Supplementary Table 7 contains the names we proposed for all the new SGI1-REs described in this study.

## Conclusion

The *trmE-*specific SGI1-REs with gene synteny are widely distributed in gamma-proteobacteria. Their backbone consists of a large similar part and another more variable part. Their diversity would be higher than what has been reported so far in the bacteria of medical importance. Environmental bacteria without pathogenicity characteristics that harbor SGI1-REs can be considered as reservoirs of GIs for bacteria responsible for human infections. Mosaicism of SGI1-REs would result from recombination events, and they would then evolve independently. Their plasticity and horizontal transfer might play a role in the genome evolution. All SGI1-REs can acquire an integron, except some that lack a resolvase gene, and participate in the spread of ARGs.

## Data Availability Statement

The original contributions presented in the study are included in the article/Supplementary Material, further inquiries can be directed to the corresponding author.

## Author Contributions

ES performed the data analysis and wrote the manuscript. ES and CN designed the study. CN revised the manuscript. Both authors approved the final version.

## Conflict of Interest

The authors declare that the research was conducted in the absence of any commercial or financial relationships that could be construed as a potential conflict of interest.

## Publisher’s Note

All claims expressed in this article are solely those of the authors and do not necessarily represent those of their affiliated organizations, or those of the publisher, the editors and the reviewers. Any product that may be evaluated in this article, or claim that may be made by its manufacturer, is not guaranteed or endorsed by the publisher.

## Footnotes

1
https://blast.ncbi.nlm.nih.gov/Blast.cgi

2
https://meme-suite.org/meme/tools/fimo

3
https://mafft.cbrc.jp/alignment/server/

4
https://www.ebi.ac.uk/Tools/msa/clustalo/
