# Supplementary material for: Overview of Salmonella Genomic Island 1-Related Elements Among Gamma-Proteobacteria Reveals Their Wide Distribution Among Environmental Species
Source: Front Microbiol. 2022 Apr 11;13:857492. doi: 10.3389/fmicb.2022.857492 (PMC9035990; doi:10.3389/fmicb.2022.857492)

Supplementary Figure 1. CLUSTAL Omega multiple sequence alignment of integrase aminoacid sequences of SGI1-REs used as a reference.

The meaning of the AA color code is as follows: red (small + hydrophobic), blue (acidic), pink (basic), and green (hydroxly + sulfhydryl + amine + G). The following symbols denote the degree of residue conservation. Asterix (*) indicates positions which have a single, fully conserved residue; colon (:) indicates conservation between groups of strongly similar properties; and period(.) indicates conservation between groups of weakly similar properties. The catalytic residue tyrosine (Y) is surrounded by three basic residues at the active site (highlighted in yellow: R-H-RH-Y). The Int_{GI} active site is conserved for all 126 SGI1-REs in this study.

```
AGI1_KP054476                       MFFSFLFTSPVFFWSYIALCVPQPYNRGEHGGKPSMKVSIEKRQPDREGKRSLRLVYYYG   60
SGI1_AF261825                       -----------------------------------MKVSVNKRNPNSKGLQQLRLVYYYG   25
SGI1-RE5SsW3-18-1_CP000503          -----------------------------------MKVSVNKRNPNSKGLQQLRLVYYYG   25
PGI1-PmESC_KU499917                 -----------------------------------MKVSVNKRNPNSKGLQQLRLVYYYG   25
PGI2_MG201402                       -----------------------------------MKVSVNKRNPNSKGLQQLRLVYYYG   25
SGI1-RE6ItCC-PW-9_PIQH01000002      -----------------------------------MKVSVNKRNPNSKGLQQLRLVYYYG   25
SGI1-RE7MaKG14_JABEVQ010000005      -----------------------------------MKVSVNKRNPNSKGLQQLRLVYYYG   25
                                                                       ****::**:*: :* :.********

AGI1_KP054476                       SN-VEDGQRKLKRSHEPLDIFIYDKPRSPAEREHNKEALRIAEAVRSKRLLESETSKHKL   119
SGI1_AF261825                       VVEGEDGKKRAKRDYEPLELYLYENPKTQAERQHNKEMLRQAEAARSARLVESHSNKFQL   85
SGI1-RE5SsW3-18-1_CP000503          VVEGEDGKKRAKRDYEPLELYLYENPKTQAERQHNKEMLRQAEAARSARLVESHSNKFQL   85
PGI1-PmESC_KU499917                 VVEGEDGKKRPKRDYEPLELYVYQSPKTQAERQHNKEMLRQAEAARSARLVESHANKFQL   85
PGI2_MG201402                       VVEGEDGKKRPKRDYEPLELYVYQNPKTQAERQHNKEMLRQAEAARSARLVESHANKFQL   85
SGI1-RE6ItCC-PW-9_PIQH01000002      VVEGEDGKKRPKRDYEPLELYVYHNPKTQAERQHNKEMMRQAEAARSARLVEAHANKFQL   85
SGI1-RE7MaKG14_JABEVQ010000005      VVEGEDGKKRPKRDYEPLELYVYHNPKTQAERQHNKEMMRQAEAARSARLVESHANKFQL   85
                                        ***::: **.:***::::*..*:: ***:**** :* ***.** **:*:.:.*.:*

AGI1_KP054476                       EDRTKLSASFFDYYDSVTVSKASGSKSNYSIWISAGHHLRRYHGRAELTFEDIDRAFLEG   179
SGI1_AF261825                       EDRVKLASSFYDYYDKLTASKESGSSSNYSIWISAGKHLRSYHGRAELTFEEIDKKFLEG   145
SGI1-RE5SsW3-18-1_CP000503          EDRVKLASSFYEYYDKLTASKESGSSSNYSIWISAGKHLRSYHGRAELTFEELDKKFLEG   145
PGI1-PmESC_KU499917                 EDRVKLASSFYEYYDRLTSTKESGSTSNYSIWVSAGKHLRSYHGRAELTFEEIDKKFLEG   145
PGI2_MG201402                       EDRVKLASSFYEYYDRLTSTKEPGSTSNYSIWVSAGKHLRSYHGRAELTFEEIDKKFLEG   145
SGI1-RE6ItCC-PW-9_PIQH01000002      EDRVKLASSFFDYYDQLTSTKESGSASNYSIWISAGKHLRNYHGRAELTFEEIDKQFLEG   145
SGI1-RE7MaKG14_JABEVQ010000005      EDRVKLASSFFDYYDQLTSTKESGSASNYSIWISAGKHLRNYHGRAELTFEEIDKPFLEG   145
                                    ***.**::**::*** :* :*  ** ******:***:*** **********::*: ****

AGI1_KP054476                       FKTYLLKSATTKSDQLLSRNTVSSYFNKIRAALNQAYQEGIIRDNPVRTVKSVKPETNQR   239
SGI1_AF261825                       FRKYLLEEPLTKSQSKLAKNTASSYFNKVRAALNEAFREGIIRDNPVQRVKSVKAENTQR   205
SGI1-RE5SsW3-18-1_CP000503          FRKYLLEEPLTKSKSKLAKNTASSYFNKVRAALNEAYREGIIRDNPVQRVKSVKAENTQR   205
PGI1-PmESC_KU499917                 FRKYLLEEPLTKSKSKLAKNTASSYFNKVRAALNEAYREGIIRDNPVQRVKSVKAENTKR   205
PGI2_MG201402                       FRKYLLEEPLTKSESKLAKNTASSYFNKVRAALNEAYREGIIRDNPVQRVKSVKAENTKR   205
SGI1-RE6ItCC-PW-9_PIQH01000002      LRKYLLEEKLTKSKTKLAKNTASSYFNKIRAALNEAYREGIIRDNPVQRVKSVKPENTKR   205
SGI1-RE7MaKG14_JABEVQ010000005      FRKYLLEEKLTKSKTRLAKNTASSYFNKVRAALNEAYREGMIRDNPVQRVKSVKAENTKR   205
                                    ::.***:.  ***.  *::**.******:*****:*::**:******: ***** *..:*

AGI1_KP054476                       VYLTLEEIKAMAKAECRYDVLKRAFLFSCTTGLRWSDIQKLVWSEVEEFEQGHFRIIFKQ   299
SGI1_AF261825                       TYLTLDEVRAMTKAECRYDVLKRAFLFSCTTGLRWSDIQKLTWKEIEEFQDGHYRIIFKQ   265
SGI1-RE5SsW3-18-1_CP000503          TYLTLDEVRAMTRAECRYDVLKRAFLFSCTTGLRWSDIQKLTWKEIEEFQDGHYRIIFKQ   265
PGI1-PmESC_KU499917                 TYLTLDEVRAMTKAECRYDVLKRAFLFSCTTGLRWSDINKLTWSEIEEFEPGHYRIIFDQ   265
PGI2_MG201402                       TYLTLDEVRAMTKAECRYDVLKRAFLFSCTTGLRWSDINKLTWSEIEEFEPGHYRIIFDQ   265
SGI1-RE6ItCC-PW-9_PIQH01000002      TYLSLDEVRALTKAECRYDVLKRAFLFSCTTGLRWSDINKLTWSEIEEFEPGHYRIIFDQ   265
SGI1-RE7MaKG14_JABEVQ010000005      TYLSLDEVRALTKAECRYDVLKRAFLFSCTTGLRWSDINKLTWREIEEFEPGHYRIIFDQ   265
                                    .**:*:*::*:::*************************:**.* *:***: **:****.*

AGI1_KP054476                       KKIQNRGTALQYLDLPDSAVRLMGE--RKDNDERVFKALRYSSYTNVALLHWAMLAGITK   357
SGI1_AF261825                       AKLLNAGNSLVYLDLPDSAVKLMGE--RQDKAERVFKGLKYSSYTNVALLHWAMLAGVQK   323
SGI1-RE5SsW3-18-1_CP000503          TKLLNAGNSLVYLDLPDSAVKLMGE--RQDKTERVFKGLKYSSYTNVALLHWAMLAGVQK   323
PGI1-PmESC_KU499917                 QKLKNGGNSLVYLDLPDSAVTLLNIQNKEGPDDRVFVGLKYNSYMNVALLQWAMRAGITK   325
PGI2_MG201402                       QKLKNGGNSLVYLDLPDSAVTLLNIQNREGPDDRVFVGLKYNSYMNVALLQWAMRAGITK   325
SGI1-RE6ItCC-PW-9_PIQH01000002      KKLKNGGKSLVYLDLPDSAVKLLDIDRKGEPEDRVFVGLKYNSYFNVALEKWAMRAGITK   325
SGI1-RE7MaKG14_JABEVQ010000005      QKLKNGGNSLVYLDLPDSAVKLLDIDQKGEPDDRVFVGLKYNSYFNVALLQWAMRAGITK   325
                                     *: * *.:* ********* *:.   :    :*** .*:*.** **** :*** **: *

AGI1_KP054476                       HVTFHAGRHSFAVNQLARGLDIYSLSRLLGHSELKTTEIYADILDQRRRDAMRSFPDIFA   417
SGI1_AF261825                       HVTFHVGRHTFAVAQLNRGVDIYSLSRLLGHSELRTTEIYADILESRRVTAMRGFPDIFE   383
SGI1-RE5SsW3-18-1_CP000503          HVTFHVGRHTFAVAQLNRGVDIYSLSRLLGHSELRTTEIYADILESRRVTAMRGFPDIFE   383
PGI1-PmESC_KU499917                 HVTFHAGRHTFAVAQLNRGVDIYSLSRLLGHSELRTTEIYADILESRRVTAMRSFPDIFE   385
PGI2_MG201402                       HVTFHAGRHTFAVAQLNRGVDIYSLSRLLGHSELRTTEIYADILESRRVTAMRGFPDIFE   385
SGI1-RE6ItCC-PW-9_PIQH01000002      HVTFHVGRHTFAVAQLNRGVDIYSLSRLLGHSELRTTEDYADILESRRVTAMRSFPDIFK   385
SGI1-RE7MaKG14_JABEVQ010000005      HVTFHAGRHTFAVAQLNRGVDIYSLSRLLGHSELRTTEIYADILESRRVMAMRSFPDIFK   385
                                    *****.***:*** ** **:**************:*** *****:.**  ***.*****

AGI1_KP054476                       DSL-------------------        420
SGI1_AF261825                       DKVQESGTCCPHCGKSVLNKTL        405
SGI1-RE5SsW3-18-1_CP000503          DKVKEPETCCPHCGKSVPHKTL        405
PGI1-PmESC_KU499917                 EQAKEFGSCCPHCGQQANP---        404
PGI2_MG201402                       DKVKESGACCPHCGKSVMNKTL        407
SGI1-RE6ItCC-PW-9_PIQH01000002      ERESENN-RCSHCGQAVPMAIA        406
SGI1-RE7MaKG14_JABEVQ010000005      ERESEDN-RCSRCGQAVPMAIA        406
```

Supplementary Figures 2. BLAST alignment of the nucleotide sequences of the backbones of SGI1-REs with the reference GI as request.

The colors of the strains correspond to the clusters of SGI1-REs: red (cluster1: SGI1 cluster), green (cluster 2: PGI1 cluster), blue (cluster 3: PGI2 cluster), purple (cluster 4: AGI1 cluster), brown (cluster 5), black (cluster 6), and grey (cluster 7). The characterized SGI1-REs are underlined. The ORFs of the backbone used as a reference are represented. The following colors are used for ORFs of known function:

- DNA recombination (*int*, *xis*)
- DNA replication (*rep*)
- Transcriptional regulator
- Type IV secretion system subunit (*tra*)
- Transcriptional activator
- Mobilization gene (*mps*)
- Resolvase (*res*)
- Toxin-antitoxin system (subtilisin-ATPase)
- DNA replication, recombination, and repair (helicase-endonuclease (*ybjD*))
- DNA restriction/modification
- Unknown function

The green angled arrows indicate AcaCD binding sites, the red arrow indicates *oriT* and the black arrow indicates the MDR region. Black vertical lines represent *attL* and *attR* attachment sites and the chromosomal *trmE* gene is shown in black. Backbone variations are described for each SGI1-RE.

(2A) Alignment of SGI1-REs of the SGI1 cluster (cluster 1) with the SGI1 backbone.

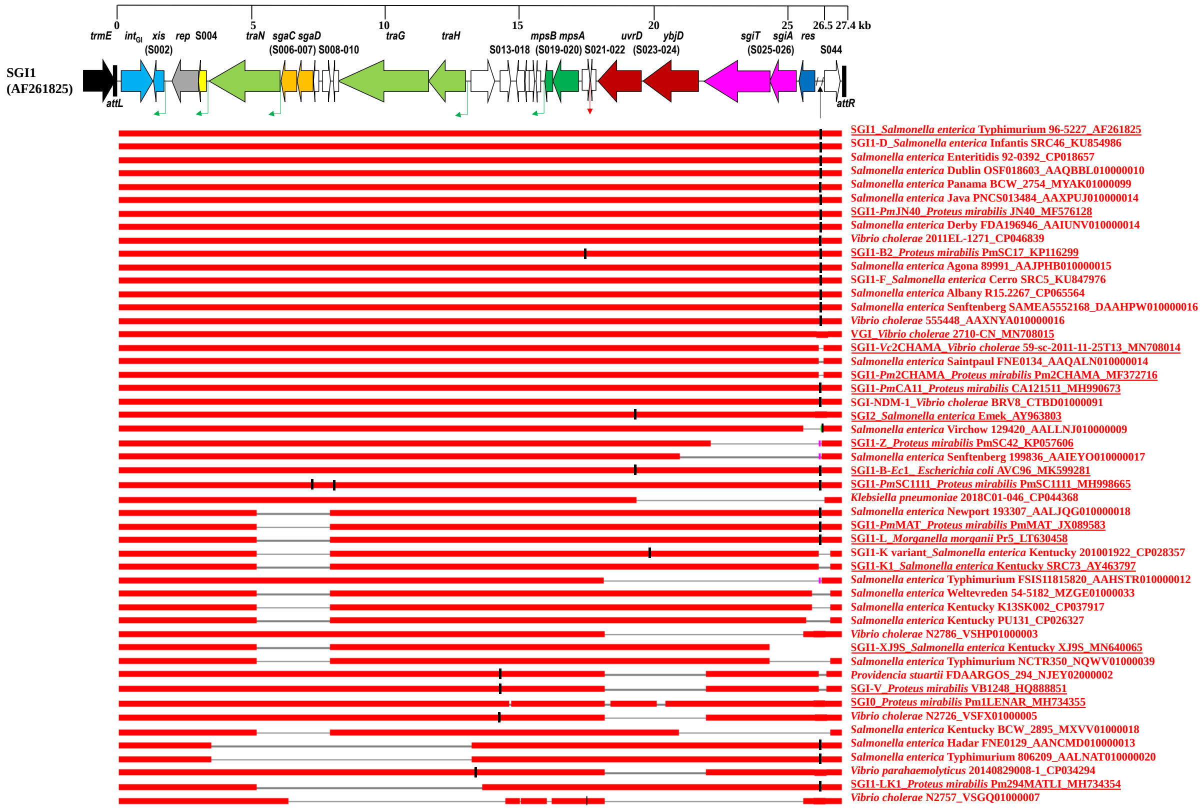

<u>SGI1 *Salmonella enterica* Typhimurium 96-5227 (AF261825)</u> (as a reference); ORFs and genes encoding proteins of known function: **S001** (***int***$_{\mathbf{GI}}$) (integrase), **S002** (***xis***) (excisionase), **S003** (***rep***) (replication protein), **S004** (helix-turn-helix domain-containing protein), **S005** (***traN***) (conjugal transfer protein), **S006** (***sgaC***) (flagellar transcriptional activator subunit C), **S007** (***sgaD***) (flagellar transcriptional activator subunit D), **S011** (***traG***) (conjugal transfer protein), **S012** (***traH***) (conjugal transfer protein), **S019** (***mpsB***) (mobilization protein MpsB), **S020** (***mpsA***) (mobilization protein MpsA), **S023** **(*uvrD*)** (ATP-dependent helicase), **S024** **(*ybjD*)** (ATP-dependent endonuclease), **S025** (***sgiT***) (toxin: subtilisin protease), **S026** (***sgiA***) (antitoxin: AAA-ATPase), **S027** (***res***) (resolvase).

SGI1-D *Salmonella enterica* Infantis SRC46 (KU854986): complete backbone.

***Salmonella enterica* Enteritidis 92-0392 (CP018657):** complete backbone.

***Salmonella enterica* Dublin OSF018603 (AAQBBL010000010):** complete backbone.

***Salmonella enterica* Panama BCW_2754 (MYAK01000099/4/781/962):** complete backbone.

***Salmonella enterica* Java PNCS013484 (AAXPUJ010000014):** complete backbone.

SGI1-*Pm*JN40 *Proteus mirabilis* JN40 (MF576128): complete backbone. (SGI1-RE followed by another GI in tandem).

***Salmonella enterica* Derby FDA196946 (AAIUNV010000014):** complete backbone.

***Vibrio cholerae* 2011EL-1271 (CP046839):** complete backbone.

SGI1-B2 *Proteus mirabilis* PmSC17 (KP116299): complete backbone; additional MDR region in **S021**.

***Salmonella enterica* Agona 89991 (AAJPHB010000015):** complete backbone.

SGI1-F *Salmonella enterica* Cerro SRC5 (KU847976): complete backbone.

***Salmonella enterica* Albany R15.2267 (CP065564):** complete backbone.

***Salmonella enterica* Senftenberg SAMEA5552168 (DAAHPW010000016):** complete backbone.

***Vibrio cholerae* 555448 (AAXNYA010000016):** complete backbone. (SGI1-RE followed by another GI in tandem).

VGI *Vibrio cholerae* 2710-CN (MN708015): complete backbone; absence of MDR region.

SGI1-*Vc*2CHAMA *Vibrio cholerae* 59-sc-2011-11-25T13 (MN708014): deletion of **part of S044** due to insertion of the MDR region.

***Salmonella enterica* Saintpaul FNE0134 (AAQALN010000014/15):** deletion of **part of S044** due to insertion of the MDR region.

SGI1-*Pm*2CHAMA *Proteus mirabilis* Pm2CHAMA (MF372716): deletion of **part of S044** due to insertion of the MDR region.

SGI1-PmCA11 *Proteus mirabilis* CA121511 (MH990673): complete backbone.

SGI-NDM-1 *Vibrio cholerae* BRV8 (CTBD01000091): complete backbone; SGI1-RE carrying bla_{NDM1} and *armA*.

SGI2 *Salmonella enterica* Emek SRC19 (AY963803): complete backbone; MDR region in **S023**.

Salmonella enterica* Virchow 129420 (AALLNJ010000009):** deletion of **part of *res and **part of the right end** of the backbone upstream of S044 due to the insertion of an IS element of the MDR region.

SGI1-Z *Proteus mirabilis* PmSC42 (KP057606): deletion of **part of S025 and S026-*res*** due to the insertion of an IS element of the MDR region.

Salmonella enterica* Senftenberg 199836 (AAIEYO010000017):** deletion of **part of S024 and S025-*res due to the insertion of an IS element of the MDR region.

SGI1-B-*Ec*1 *Escherichia coli* AVC96 (MK599281): insertion of IS*Ec43* in **S023**.

SGI1-*Pm*SC1111 *Proteus mirabilis* PmSC1111 (MH998665): insertion of an IS element of the IS*3* family in **S007**; insertion of part of the MDR region in **S010**; **part of S010-*res*** in inverse orientation due to the presence of IS*26* elements in the MDR region.

***Klebsiella pneumoniae* 2018C01-046 (CP044368):** deletion of **part of S023, S024-*res* and part of S044** due to insertion of the MDR region.

***Salmonella enterica* Newport 193307 (AALJQG010000018):** deletion of **part of *traN*, S006-S008 and part of S009** due to insertion of IS*Vch4*.

SGI1-*Pm*MAT *Proteus mirabilis* PmMAT (JX089583): deletion of **part of *traN*, S006-S008 and part of S009**.

SGI1-L *Morganella morganii* Pr5 (LT630458): deletion of **part of *traN*, S006-S008 and part of S009** due to insertion of IS*Vch4*.

SGI1-K variant *Salmonella enterica* Kentucky 201001922 (CP028357): deletion of **part of *traN*, S006-S008 and part of S009** due to insertion of IS*Vch4*; insertion of part of the MDR region in **S024**; deletion of **part of S044** due to insertion of the MDR region.

SGI1-K1 *Salmonella enterica* Kentucky SRC73 (AY463797): deletion of **part of *traN*, S006-S008 and part of S009** due to insertion of IS*Vch4*; deletion of **part of S044** due to insertion of the MDR region.

Salmonella enterica* Typhimurium FSIS11815820 (AAHSTR010000012):** deletion of **S023-*res due to the insertion of an IS element of the MDR region.

Salmonella enterica* Weltevreden 54-5182 (MZGE01000033/781):** deletion of **part of *traN*, S006-S008 and part of S009** due to insertion of IS*Vch4*; deletion of **part of *res and **part of S044** due to insertion of the MDR region.

Salmonella enterica* Kentucky K13SK002 (CP037917):** deletion of **part of *traN*, S006-S008 and part of S009** due to insertion of IS*Vch4*; deletion of **part of *res and **part of S044** due to insertion of the MDR region.

Salmonella enterica* Kentucky PU131 (CP026327):** deletion of **part of *traN*, S006-S008 and part of S009** due to insertion of IS*Vch4*; deletion of **part of *res and **part of S044** due to insertion of an IS*26* element.

Vibrio cholerae* N2786 (VSHP01000003): S023-*res region composed of 5 ORFs: 2 ORFs share nt identity with the Sputw3181_4077 and Sputw3181_4076 loci of *Shewanella* sp. W3-18-1 (CP000503) and 3 ORFs encoding an *Xcy*I family restriction endonuclease, a site-specific DNA-methyltransferase and a resolvase; absence of MDR region.

SGI1-XJ9S *Salmonella enterica* Kentucky XJ9S (MN640065): deletion of **part of S025, S026-*res* and S044** due to insertion of the MDR region.

Salmonella enterica* Typhimurium NCTR350 (NQWV01000039/27):** deletion of **part of *traN*, S006-S008 and part of S009** due to insertion of IS*Vch4*; deletion of **part of S025, S026-*res and **part of S044** due to insertion of the MDR region.

***Providencia stuartii* FDAARGOS_294 (NJEY02000002):** insertion of 3 ORFs in **S014** including an ORF encoding a hydrolase; deletion of **S023-S024;** deletion of **part of S044**; SGI1-RE carrying $bla_{\text{VEB-6}}$ and *qnrA1*.

SGI-V (SGI1-V) *Proteus mirabilis* VB1248 (HQ888851): insertion of 3 ORFs in **S014** including an ORF encoding a hydrolase; deletion of **S023-S024**; deletion of **part of S044**; SGI1-RE carrying $bla_{\text{VEB-6}}$ and *qnrA1*.

SGI0 *Proteus mirabilis* Pm1LENAR (MH734355): lower nt identity for the different regions: **S014**, **S021-S022** and **S023-S024**; absence of MDR region.

***Vibrio cholerae* N2726 (VSFX01000005):** insertion of 2 ORFs coding for an MBL fold metallo-hydrolase and an AraC family transcriptional regulator in **S014**; deletion of **S023-S024**; absence of MDR region.

Salmonella enterica* Kentucky BCW_2895 (MXVV01000018):** deletion of **part of *traN*, S006-S008** and **part of S009** due to insertion of IS*Vch4*; deletion of **part of S024, S025-*res and **part of S044** due to insertion of the MDR region).

Salmonella enterica* Hadar FNE0129 (AANCMD010000013):** deletion of the ***traN-traH region**.**

Salmonella enterica* Typhimurium 806209 (AALNAT010000020):** deletion of the ***traN -traH region.

***Vibrio parahaemolyticus* 20140829008 (CP034294):** 2751 bp insertion **between *traH* and S013** including 2 ORFs, one of which encodes an immunity 49 family protein; deletion of **S023-S024**; absence of MDR region.

<u>**SGI1-LK1 *Proteus mirabilis* Pm294MATLI (MH734354):**</u> deletion of **part of *traN*, S006-S012 and part of S013** due to insertion of IS*Vch4*.

Vibrio cholerae* N2757 (VSGQ01000007):** deletion of **part of S006, S007-S013 and part of S014**; lower nt identity for the **S015-S018** region, but better nt identity with P013-P015; insertion of 2 ORFs sharing nt identity with locus A6033_05025 and a region encoding an ImmA/IrrE family metallo-endopeptidase from *Aeromonas veronii* CB51 (CP015448) **between S018 and S019**; lower nt identity for the **S021-S022** region; the **S023-*res region shares nt identity with the Sputw3181_4076 locus of *Shewanella* sp. W3-18-1 (CP000503), and 3 ORFs encoding an *Xcy*I family restriction endonuclease, a site-specific DNA-methyltransferase and a recombinase; absence of MDR region.

(2B) Alignment of SGI1-REs of the PGI1 cluster (cluster 2) with the PGI1-*Pm*ESC backbone.

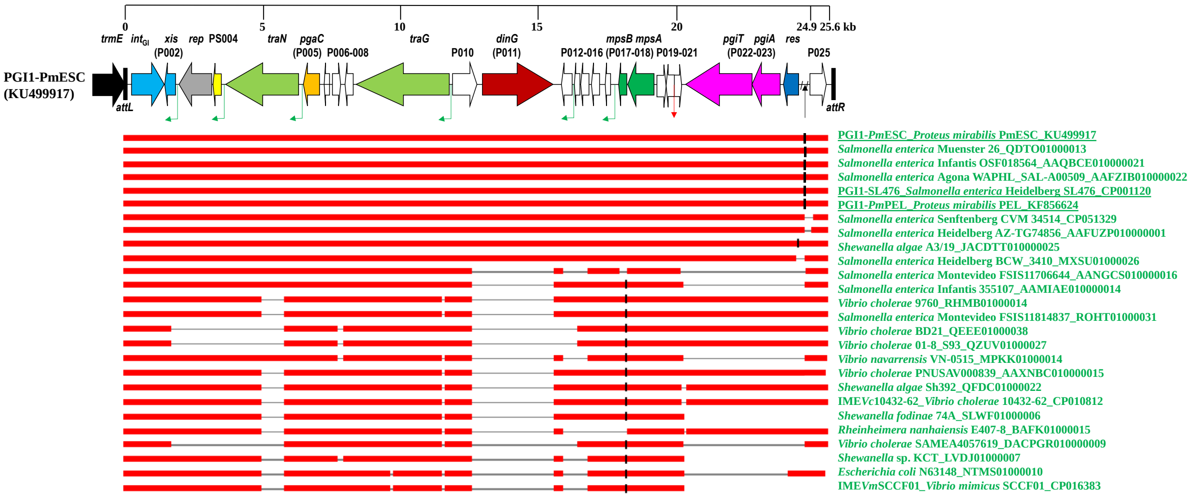

<u>**PGI1-*Pm*ESC *Proteus mirabilis* PmESC (KU499917)**</u> (as a reference); ORFs and genes encoding proteins of known function: **P001** (***int*GI**) (integrase), **P002** (***xis***) (excisionase), **P003** (***rep***) (replication protein), **PS004** (helix-turn-helix domain-containing protein), **P004** (***traN***) (conjugal transfer protein), **P005** (***pgaC***) (flagellar transcriptional activator subunit C), **P009** (***traG***) (conjugal transfer protein), **P011** **(*dinG*)** (DEAD/DEAH box helicase), **P017** (***mpsB***) (mobilization protein MpsB), **P018** (***mpsA***) (mobilization protein MpsA), **P022** (***pgiT***) (toxin: subtilisin protease), **P023** (***pgiA***) (antitoxin: AAA-ATPase), **P024** (***res***) (resolvase).

***Salmonella enterica* Muenster 26 (QDTO01000013):** complete backbone.

***Salmonella enterica* Infantis OSF018564 (AAQBCE010000021/23):** complete backbone.

***Salmonella enterica* Agona WAPHL_SAL-A00509 (AAFZIB010000022/24):** complete backbone.

<u>**PGI1-SL476 *Salmonella enterica* Heidelberg SL476 (CP001120):**</u> complete SGI1-RE inserted in the intergenic region between *sodB* and *purR*.

__PGI1-*PmPEL* *Proteus mirabilis* PEL (KF856624):__ 21-bp deletion in *int*; complete SGI1-RE carrying bla_{VEB-6}, bla_{NDM1} and bla_{DHA-1}).

Salmonella enterica **Senftenberg CVM 34514 (CP051329): P025** truncated by an IS element of the MDR region.

Salmonella enterica **Heidelberg AZ-TG74856 (AAFUZP010000001):** SGI1-RE inserted in the intergenic region between *sodB* and *purR***; P025** truncated by an IS element of the MDR region.

Shewanella algae **A3/19 (JACDTT010000025/49):** insertion of an IS*26* element in ***res***; absence of MDR region.

Salmonella enterica **Heidelberg BCW_3410 (MXSU01000026/6):** SGI1-RE inserted in the intergenic region between *sodB* and *purR*; ***res*** truncated by an IS element of the MDR region.

Salmonella enterica **Montevideo FSIS11706644 (AANGCS010000016): P011-P014** region shares nt identity with the A6033_05010 and A6033_05015 loci of *Aeromonas veronii* CB51 (CP015448); region **between P016 and P017** shares nt identity with a region of *Aeromonas veronii* CB51 containing 2 ORFs, one of which encodes an ImmA/IrrE family metallo-endopeptidase; **P021-*res*** region shares nt identity with A023 and A024; absence of MDR region.

Salmonella enterica **Infantis 355107 (AAMIAE010000014): P011** region shares nt identity with the A6033_05005 and A6033_05010 loci of *Aeromonas veronii* CB51 (CP015448); longer intergenic region **between P016 and P017**; **P021-*res*** region corresponds to an 8.5 kb segment with 7 ORFs showing no significant identity to known gene sequences, including ORFs encoding a DNA helicase, a macro domain-containing protein, 2 DUF4433 domain-containing proteins, and a resolvase; absence of MDR region.

Vibrio cholerae **9760 (RHMB01000014):** very low nt identity with PGI1 for ***traN***; *traH* found upstream of ***traG***; **P011** region shares nt identity with the A6033_05010 locus of *Aeromonas veronii* CB51 (CP015448); longer intergenic region **between P016 and P017**; absence of MDR region.

Salmonella enterica **Montevideo FSIS11814837 (ROHT01000031):** very low nt identity with PGI1 for ***traN***; *traH* found upstream of ***traG***; **P011** region shares nt identity with the A6033_05010 locus of *Aeromonas veronii* CB51 (CP015448); longer intergenic region **between P016 and P017**; absence of MDR region.

Vibrio cholerae **BD21 (QEEE01000038): *rep-traN*** region shares nt identity with the FE240_17910, FE240_17915 and FE240_17920 loci encoding a TrfA protein, a DNA-binding protein and TraN in *Aeromonas simiae* A6 (CP040449); region **between P007 and P008** shares nt identity with the A6033_04990 locus of *Aeromonas veronii* CB51 (CP015448); **P011-P012** region shares nt identity with the FE240_17960 and FE240_17965 loci of *Aeromonas simiae* A6; longer intergenic region **between P016 and P017**; absence of MDR region.

Vibrio cholerae **01-8_S93 (QZUV01000027): *rep-traN*** region shares nt identity with the FE240_17910, FE240_17915 and FE240_17920 loci encoding a TrfA protein, a DNA-binding protein and TraN in *Aeromonas simiae* A6 (CP040449); region **between P007 and P008** shares nt identity with the A6033_04990 locus of *Aeromonas veronii* CB51 (CP015448); **P011-P012** region shares nt identity with the FE240_17960 and FE240_17965 loci of *Aeromonas simiae* A6; longer intergenic region **between P016 and P017**; absence of MDR region.

Vibrio navarrensis **VN-0515 (MPKK01000014):** region **between P007 and P008** shares nt identity with the A6033_04990 locus of *Aeromonas veronii* CB51 (CP015448); *traH* found upstream of ***traG***; **P011-P014** region shares nt identity with the A6033_05010 and A6033_05015 loci of *Aeromonas veronii* CB51; longer intergenic region **between P016 and P017**; **P021-*res*** region corresponds to an 8.5 kb segment with 7 ORFs showing no significant identity to known gene sequences, including ORFs encoding a DNA helicase, a macro domain-containing protein, 2 DUF4433 domain-containing proteins, and a resolvase; absence of MDR region.

Vibrio cholerae **PNUSAV000839 (AAXNBC010000015/39):** very low nt identity with PGI1 for ***traN***; *traH* found upstream of ***traG***; **P011** region shares nt identity with the A6033_05010 locus of *Aeromonas veronii* CB51 (CP015448); longer intergenic region **between P016 and P017**; absence of MDR region.

Shewanella algae **Sh392 (QFDC01000022/74/49):** very low nt identity with PGI1 for ***traN***; *traH* found upstream of ***traG***; **P011** region shares nt identity with the A6033_05010 locus of *Aeromonas veronii* CB51 (CP015448);

longer intergenic region **between P016 and P017**; region **between P020 and P022** shares nt identity with A023 and A024; absence of MDR region.

IME*Vc*10432-62 *Vibrio cholerae* 10432-62 (CP010812): very low nt identity with PGI1 for ***traN***; *traH* found upstream of ***traG***; **P011** region shares nt identity with the A6033_05010 locus of *Aeromonas veronii* CB51 (CP015448); longer intergenic region **between P016 and P017**; region **between P020 and P022** shares nt identity with A023-A024; absence of MDR region.

Shewanella fodinae* 74A (SLWF01000006):** very low nt identity with PGI1 for ***traN; *traH* found upstream of ***traG***; **P011** region shares nt identity with the A6033_05010 locus of *Aeromonas veronii* CB51 (CP015448); longer intergenic region **between P016 and P017**; **P022-*res*** region shares nt identity with S023-S024 followed by 3 ORFs encoding a *Bsu*BI/*Pst*I restriction endonuclease, an N-6 DNA methylase and a resolvase; **P025** region corresponds to an ORF encoding a DUF4365 domain-containing protein.

Rheinheimera nanhaiensis* E407-8 (BAFK01000015):** very low nt identity with PGI1 for ***traN; *traH* found upstream of ***traG***; **P011-P016** region shares nt identity with the A6033_05010 locus of *Aeromonas veronii* CB51 (CP015448) and PGI2-015-PGI2-018; region **between P020 and P022** shares nt identity with S023-S024; absence of MDR region.

Vibrio cholerae* SAMEA4057619 (DACPGR010000009): *rep-traN region shares nt identity with the FE240_17910, FE240_17915 and FE240_17920 loci encoding a TrfA protein, a DNA-binding protein and TraN in *Aeromonas simiae* A6 (CP040449); ***traH*** found upstream of ***traG***; **P011-P012** region shares nt identity with the FE240_17960 and FE240_17965 loci of *Aeromonas simiae* A6; longer intergenic region **between P016 and P017**; **P021-*res*** region corresponds to an 8.5 kb segment with 7 ORFs showing no significant identity to known gene sequences, including ORFs encoding a DNA helicase, a macro domain-containing protein, 2 DUF4433 domain-containing proteins, and a resolvase; absence of MDR region.

Shewanella* sp. KCT (LVDJ01000007):** very low nt identity with PGI1 for ***traN; region **between P007 and P008** shares nt identity with the A6033_04990 locus of *Aeromonas veronii* CB51 (CP015448); region **between P010 and P015** shares nt identity with the A6033_05010 and A6033_05015 loci of *Aeromonas veronii* CB51; longer intergenic region **between P016 and P017**; **P022-*res*** region shares nt identity with S023-S024 followed by 3 ORFs encoding a *Bsu*BI/*Pst*I restriction endonuclease, an N-6 DNA methylase and a resolvase; **P025** region corresponds to an ORF encoding a DUF4365 domain-containing protein; absence of MDR region.

Escherichia coli* N63148 (NTMS01000010):** very low nt identity with PGI1 for ***traN; low nt identity with PGI1 for ***traG***; *traH* found upstream of ***traG***; region **between P010 and P015** shares nt identity with the A6033_05010 and A6033_05015 loci of *Aeromonas veronii* CB51 (CP015448); longer intergenic region **between P016 and P017**; **P022-*res*** region shares nt identity with S023-S024 followed by 3 ORFs encoding a *Bsu*BI/*Pst*I restriction endonuclease, an N-6 DNA methylase and a resolvase; **P025** region followed by 3 ORFs encoding proteins of unknown function; absence of MDR region.

IME*Vm*SCCF01 *Vibrio mimicus* SCCF01 (CP016383): very low nt identity with PGI1 for ***traN***; low nt identity with PGI1 for ***traG***; *traH* found upstream of ***traG***; region **between P010 and P015** shares nt identity with the A6033_05010 and A6033_05015 loci of *Aeromonas veronii* CB51 (CP015448); longer intergenic region **between P016 and P017**; **P022-*res*** region shares nt identity with S023-S024 followed by 3 ORFs encoding a *Bsu*BI/*Pst*I restriction endonuclease, an N-6 DNA methylase and a resolvase; **P025** region corresponds to an ORF encoding a DUF4365 domain-containing protein; absence of MDR region.

(**2C**) Alignment of SGI1-REs of the PGI2 cluster (cluster 3) with the PGI2 backbone.

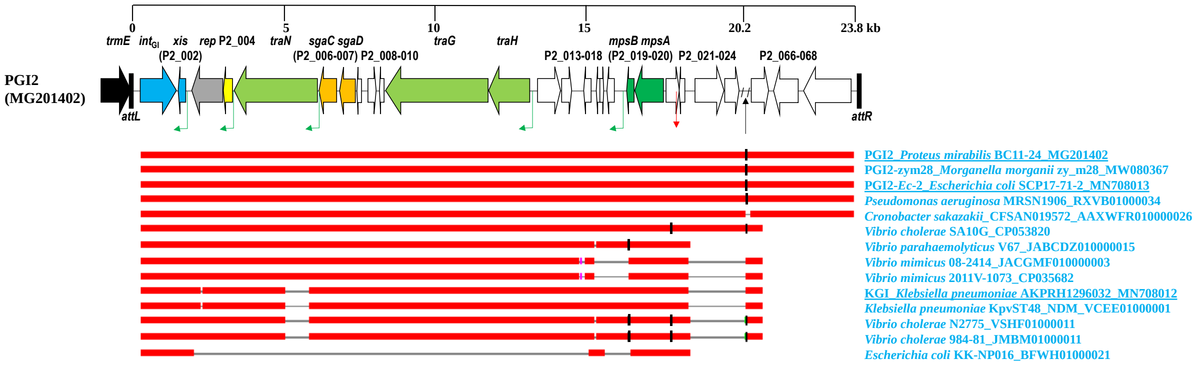

PGI2 *Proteus mirabilis* BC11-24 (MG201402) (as a reference); ORFs and genes encoding proteins of known function: **PGI2-001** (***int***$_{GI}$) (integrase), **PGI2-002** (***xis***) (excisionase), **PGI2-003** (***rep***) (replication protein), **PGI2-004** (helix-turn-helix domain-containing protein), **PGI2-005** (***traN***) (conjugal transfer protein), **PGI2-006** (***sgaC***) (flagellar transcriptional activator subunit C), **PGI2-007** (***sgaD***) (flagellar transcriptional activator subunit D), **PGI2-011** (***traG***) (conjugal transfer protein), **PGI2-012** (***traH***) (conjugal transfer protein), **PGI2-019** (***mpsB***) (mobilization protein MpsB), **PGI2-020** (***mpsA***) (mobilization protein MpsA); absence of *res* (resolvase) probably due to insertion of the MDR region.

PGI2-zym28 *Morganella morganii* zy_m28 (MW080367): complete backbone; absence of *res* probably due to insertion of the MDR region. SGI1-RE carrying *bla*$_{CTXM-3}$, *fosA3* and *aac*(6')-Ib-cr.

PGI2-*Ec*2 *Escherichia coli* SCP17-71-2 (MN708013): complete backbone; absence of *res* probably due to insertion of the MDR region.

***Pseudomonas aeruginosa* MRSN1906 (RXVB01000034/21):** complete backbone; absence of *res* probably due to insertion of the MDR region.

***Cronobacter sakazakii* CFSAN019572 (AAXWFR010000026/21):** region **between PGI2-024 and PGI2-066** shares nt identity with A025-*res* encoding a *Bsu*BI/*Pst*I restriction endonuclease, an N-6 DNA methylase and a resolvase; **PGI2-066** truncated by insertion of the MDR region.

***Vibrio cholerae* SA10G (CP053820):** very low nt identity with PGI2 for the **PGI2-021-PGI2-022** region and the region is longer; region **between PGI2-024 and PGI2-066** shares nt identity with A025-*res*, genes encoding a *Bsu*BI/*Pst*I restriction endonuclease, an N-6 DNA methylase and a recombinase; **P2-066-P2-068** region shares nt identity with P025; absence of MDR region.

***Vibrio parahaemolyticus* V67 (JABCDZ010000015):** region **between PGI2-014 and PGI2-015** shares nt identity with P012 and P013; **PGI2-023-PGI2-068** region corresponds to 5 ORFs encoding a mechanosensitive ion channel family protein, a magnesium and cobalt transport protein CorA, a FAD:protein FMN transferase, a calcium/sodium antiporter and an undecaprenyl-diphosphate phosphatase; absence of *res*; absence of MDR region.

***Vibrio mimicus* 08-2414 (JACGMF010000003):** region **between PGI2-014 and PGI2-019** shares nt identity with the FE240_17965 locus of *Aeromonas simiae* A6 (CP040449), P013-P016 which shares nt identity with the FE240_17970, FE240_17975, FE240_17980 and FE240_17985 loci of *Aeromonas simiae* A6, and the FE240_17990 locus of *Aeromonas simiae* A6 encoding a permease; region **between PGI2-022 and PGI2-066** shares nt identity with S023-S024, followed by 3 ORFs encoding a *Bsu*B*I/PstI* restriction endonuclease, an N-6 DNA methylase and a resolvase; **P2-066-P2-068** region shares nt identity with P025; absence of MDR region.

***Vibrio mimicus* 2011V-1073 (CP035682):** region **between PGI2-014 and PGI2-019** shares nt identity with the FE240_17965 locus of *Aeromonas simiae* A6 (CP040449), P013-P016 which shares nt identity with the FE240_17970, FE240_17975, FE240_17980 and FE240_17985 loci of *Aeromonas simiae* A6, and the FE240_17990 locus of *Aeromonas simiae* A6 encoding a permease; region **between PGI2-022 and PGI2-066** shares nt identity with S023-S024, followed by 3 ORFs encoding a *Bsu*B*I/PstI* restriction endonuclease, an N-6 DNA methylase and a resolvase; **P2-066-P2-068** region shares nt identity with P025; absence of MDR region.

<u>KGI_*Klebsiella pneumoniae* AKPRH1296032 (MN708012):</u> deletion **between PGI2-002 and *rep***; **central region of *traN*** shares nt identity with P004 (*traN*); **PGI2-023-PGI2-024** region shares nt identity with S023-*res*; **PGI2-066-PGI2-068** region shares nt identity with P025; absence of MDR region.

Klebsiella pneumoniae* KpvST48_NDM (VCEE01000001):** deletion **between PGI2-002 and *rep; **central region of *traN*** shares nt identity with P004 (*traN*); **PGI2-023-PGI2-024** region shares nt identity with S023-*res*; **PGI2-066-PGI2-068** region shares nt identity with P025; absence of MDR region.

Vibrio cholerae* N2775 (VSHF01000011/12): central region of *traN shares nt identity with P004 (*traN*); **PGI2-015** region shares nt identity with P012-P014; longer **intergenic region between PGI2-018 and PGI2-019**; longer **region PGI2-020 -PGI2-021**; **PGI2-023-PGI2-024** region shares nt identity with the Sputw3181_4076 locus of *Shewanella* sp. W3-18-1 (CP000503), and with A025-*res* encoding a *Bsu*BI/*Pst*I restriction endonuclease, an N-6 DNA methylase and a resolvase; **PGI2-066-PGI2-068** region shares nt identity with P025. (SGI1-RE followed by another GI in tandem).

Vibrio cholerae* 984-81 (JMBM01000011): central region of *traN shares nt identity with P004 (*traN*); **PGI2-015** region shares nt identity with P012-P014; longer **intergenic region between PGI2-018 and PGI2-019**; longer region **PGI2-020 -PGI2-021**; **PGI2-023-PGI2-024** region shares nt identity with the Sputw3181_4076 locus of *Shewanella* sp. W3-18-1 (CP000503), and with A025-*res* encoding a *Bsu*BI/*Pst*I restriction endonuclease, an N-6 DNA methylase and a resolvase; **PGI2-066-PGI2-068** region shares nt identity with P025.

***Escherichia coli* KK-NP016 (BFWH01000021):** most of the backbone (**PGI2-002-PGI2-024** region) shares nt identity with PGI1 regions: P002-P010 region; followed by P011-P014 region that shares nt identity with the A6033_05010 and A6033_05015 loci of *Aeromonas veronii* CB51 (CP015448); P015-P016 region; region between P016 and P017 shares nt identity with a region of *Aeromonas veronii* CB51 between the A6033_05020 and A6033_05025 loci, containing an ORF encoding an ImmA/IrrE family metallo-endopeptidase; P017-P021 region followed by a region sharing nt identity with S023-S024 and 3 ORFs encoding a *Bsu*BI/*Pst*I restriction endonuclease, an N-6 DNA methylase and a resolvase; **PGI2-66-PGI2-68** region shares nt identity with *Shewanella loihica* PV-4 (CP000606) from bases 4593806 to 4593138; absence of MDR region.

(2D) Alignment of SGI1-REs of the AGI1 cluster (cluster 4) with the AGI1 backbone.

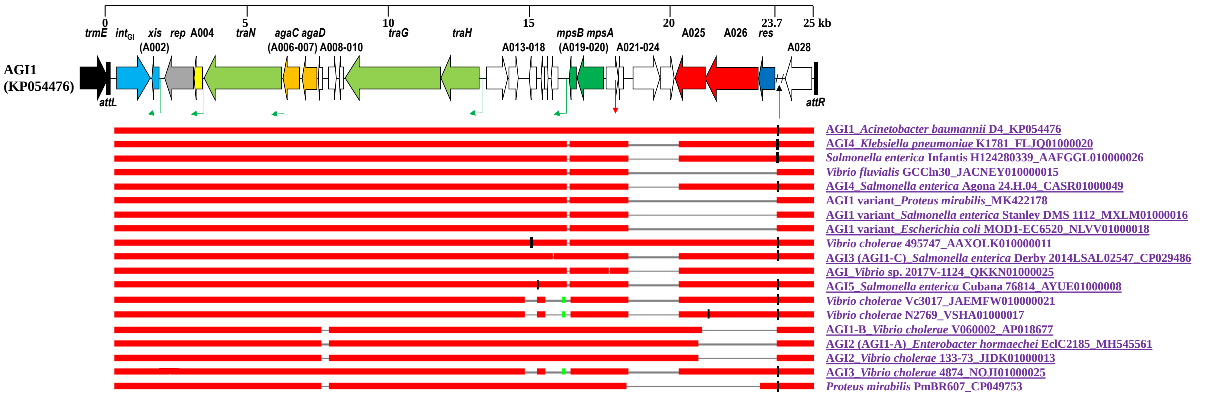

<u>AGI1 *Acinetobacter baumannii* D4 (KP054476)</u> (as a reference); ORFs and genes encoding proteins of known function: **A001** (***int*GI**) (integrase), **A002** (***xis***) (excisionase), **A003** (***rep***) (replication protein), **A004** (helix-turn-helix domain-containing protein), **A005** (***traN***) (conjugal transfer protein), **A006** (***agaC***) (flagellar transcriptional activator subunit C), **A007** (***agaD***) (flagellar transcriptional activator subunit D), **A011** (***traG***) (conjugal transfer protein), **A012** (***traH***) (conjugal transfer protein), **A019** (***mpsB***) (mobilization protein MpsB), **A020** (***mpsA***) (mobilization protein MpsA), **A025** (*Bsu*B1-*Pst*1 restriction endonuclease family protein), **A026** (N-6 DNA methylase), **A027** (***res***) (resolvase). SGI1-RE carrying $bla_{\text{PER-1}}$.

<u>AGI4 *Klebsiella pneumoniae* K1781 (FLJQ01000020/22):</u> shorter intergenic region **between A018 and A019**; **A023-A024** region corresponds to the Sputw3181_4076 locus of *Shewanella* sp. W3-18-1 (CP000503).

***Salmonella enterica* Infantis H124280339 (AAFGGL010000026/29):** shorter intergenic region **between A018 and A019**; **A023-A024** region corresponds to the Sputw3181_4076 locus of *Shewanella* sp. W3-18-1 (CP000503).

Vibrio fluvialis* GCCln30 (JACNEY010000015):** shorter intergenic region **between A018 and A019**; **A023-*res region corresponds to part of the Sputw3181_4076 locus of *Shewanella* sp. W3-18-1 (CP000503).

AGI4 *Salmonella enterica* Agona 24.H.04 (CASR01000049/44): shorter intergenic region **between A018 and A019**; **A023-A024** region corresponds to the Sputw3181_4076 locus of *Shewanella* sp. W3-18-1 (CP000503).

AGI1 variant *Proteus mirabilis* (MK422178): shorter intergenic region **between A018 and A019**; **A023-*res*** region corresponds to part of the Sputw3181_4076 locus of *Shewanella* sp. W3-18-1 (CP000503).

AGI1 variant *Salmonella enterica* Stanley DMS 1112 (MXLM01000016): shorter intergenic region **between A018 and A019**; **A023-*res*** region corresponds to part of the Sputw3181_4076 locus of *Shewanella* sp. W3-18-1 (CP000503).

AGI1 variant *Escherichia coli* MOD1-EC6520 (NLVV01000018/47/28): shorter intergenic region **between A018 and A019**; **A023-*res*** region corresponds to part of the Sputw3181_4076 locus of *Shewanella* sp. W3-18-1 (CP000503).

***Vibrio cholerae* 495747 (AAXOLK010000011):** insertion of a transposon into **A015**; shorter intergenic region **between A018 and A019**; absence of MDR region.

AGI3 (AGI1-C) *Salmonella enterica* Derby 2014LSAL02547 (CP029486): shorter intergenic region **between A017 and A018**; **A023-A024** region corresponds to the Sputw3181_4076 locus of *Shewanella* sp. W3-18-1 (CP000503).

AGI *Vibrio* sp. 2017V-1124 (QKKN01000025/42): shorter intergenic region **between A018 and A019**; shorter intergenic region **between A020 and A021**; **A023-A024** region corresponds to the Sputw3181_4076 locus of *Shewanella* sp. W3-18-1 (CP000503); absence of MDR region.

AGI5 *Salmonella enterica* Cubana 76814 (AYUE01000008/66): A015 region shares nt identity with P012-P014; shorter intergenic region **between A018 and A019**; **A023-A024** region corresponds to the Sputw3181_4076 locus of *Shewanella* sp. W3-18-1 (CP000503).

***Vibrio cholerae* Vc3017 (JAEMFW010000021): A015-A018** region shares nt identity with the FE240_17965 locus of *Aeromonas simiae* A6 CP040449), P013-P016 region that shares nt identity with the FE240_17970, FE240_17975, FE240_17980 and FE240_17985 loci of *Aeromonas simiae* A6, followed by the FE240_17990 locus of *Aeromonas simiae* A6 encoding a permease; **A023-A024** region shares nt identity with the Sputw3181_4076 locus of *Shewanella* sp. W3-18-1 (CP000503).

***Vibrio cholerae* N2769 (VSHA01000017/3): A014-A018** region shares nt identity with the FE240_17965 locus of *Aeromonas simiae* A6 CP040449), P013-P016 region that shares nt identity with the FE240_17970, FE240_17975, FE240_17980 and FE240_17985 loci of *Aeromonas simiae* A6 , followed by the FE240_17990 locus of *Aeromonas simiae* A6 encoding a permease; **A023-A024** region shares nt identity with the Sputw3181_4076 locus of *Shewanella* sp. W3-18-1 (CP000503); insertion of an IS*Vch4*-like element in **A026**.

AGI1 (AGI1-B) *Vibrio cholerae* V060002 (AP018677): deletion **between A007 and A009** leading to the loss of **A008**; deletion of part of **A025, and A026-*res*** due to insertion of the MDR region.

AGI2 (AGI1-A) *Enterobacter hormaechei* EclC2185 (MH545561): deletion **between A007 and A009** leading to the loss of **A008**; deletion of part of **A025, and A026-*res*** due to insertion of the MDR region.

AGI2 *Vibrio cholerae* 133-73 (JIDK01000013): deletion **between A007 and A009** leading to the loss of **A008**; deletion of part of **A025, and A026-*res*** due to insertion of the MDR region.

AGI3 *Vibrio cholerae* 4874 (NOJI01000025/29/33): A015-A018 region shares nt identity with the FE240_17965 locus of *Aeromonas simiae* A6 CP040449), P013-P016 region that shares nt identity with the FE240_17970, FE240_17975, FE240_17980 and FE240_17985 loci of *Aeromonas simiae* A6, followed by the FE240_17990

locus of *Aeromonas simiae* A6 encoding a permease; **A023-A024** region shares nt identity with the Sputw3181_4076 locus of *Shewanella* sp. W3-18-1 (CP000503).

Proteus mirabilis* PmBR607 (CP049753):** deletion **between A007 and A009** leading to the loss of **A008**; ***A023-res region shares nt identity with P021-*res* including ORFs encoding a subtilisin and an ATPase. SGI1-RE carrying *bla*$_{\mathrm{CTXM\text{-}2}}$.

(2E) Alignment of SGI1-REs of the cluster 5 with the SGI1-RE5*Ss*W3-18-1 backbone.

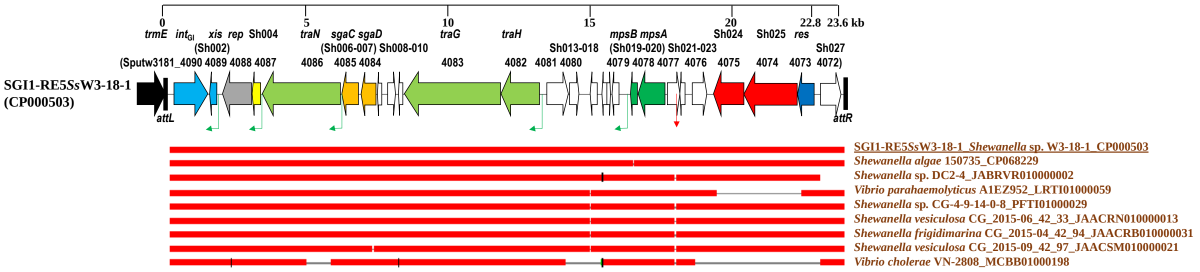

SGI1-RE5*Ss*W3-18-1 *Shewanella* sp W3-18-1 (CP000503): (as a reference); ORFs and genes encoding proteins of known function: **Sh001** (***int*GI**) (integrase), **Sh002** (***xis***) (excisionase), **Sh003** (***rep***) (replication protein), **Sh004** (helix-turn-helix domain-containing protein), **Sh005** (***traN***) (conjugal transfer protein), **Sh006** (***sgaC***) (flagellar transcriptional activator subunit C), **Sh007** (***sgaD***) (flagellar transcriptional activator subunit D), **Sh011** (***traG***) (conjugal transfer protein), **Sh012** (***traH***) (conjugal transfer protein), **Sh019** (***mpsB***) (mobilization protein MpsB), **Sh020** (***mpsA***) (mobilization protein MpsA), **Sh024** (*Bsu*B1-*Pst*1 restriction endonuclease family protein), **Sh025** (N-6 DNA methylase), **Sh026** (resolvase), absence of MDR region.

***Shewanella algae* 150735_CP068229:** shorter intergenic region **between Sh018 and Sh019.**

***Shewanella* sp DC2-4 (JABRVR010000002):** region **between Sh014 and Sh016** shares nt identity with P012-P014; shorter **Sh021-Sh022** region; **Sh027** region corresponds to an ORF encoding an AAA family ATPase; absence of MDR region.

***Vibrio parahaemolyticus* A1EZ952 (LRTI01000059):** shorter intergenic region **between Sh014 and Sh015**; **Sh024-Sh026** region corresponds to 3 ORFs encoding an *Xcy*I family restriction endonuclease, a site-specific DNA-methyltransferase and a resolvase; absence of MDR region.

***Shewanella* sp CG-4-9-14-0-8 (PFTI01000029):** (shorter intergenic region **between Sh014 and Sh015**; shorter **Sh021-Sh022** region; absence of MDR region.

***Shewanella vesiculosa* CG_2015-06_42_33 (JAACRN010000013):** shorter intergenic region **between Sh014 and Sh015**; shorter **Sh021-Sh022** region; absence of MDR region.

***Shewanella frigidimarina* CG_2015-04_42_94 (JAACRB010000031):** shorter intergenic region **between Sh014 and Sh015**; shorter **Sh021-Sh022** region; absence of MDR region.

***Shewanella vesiculosa* CG_2015-09_42_97 (JAACSM010000021/26):** shorter **Sh007** region; shorter intergenic region **between Sh014 and Sh015**; shorter **Sh021-Sh022** region; absence of MDR region.

Vibrio cholerae* VN-2808 (MCBB01000198/211/210/160):** insertion of an IS*4* element **between Sh002 and *rep; **central region of *traN*** shares nt identity with P004 (*traN*); insertion of an IS*5* element **in Sh009**; region **between Sh013 and Sh015** corresponds to the insertion of an IS*66* element and an ORF encoding a restriction endonuclease; shorter region **Sh021-Sh022**; **Sh023-*res*** region corresponds to 2 ORFs encoding an YkgJ family cysteine cluster protein and a type II toxin-antitoxin system PemK/MazF family toxin; absence of *res*; absence of MDR region.

(2F) Alignment of SGI1-REs of the clusters 6 and 7 with the SGI1-RE6*It*CC-PW-9 backbone.

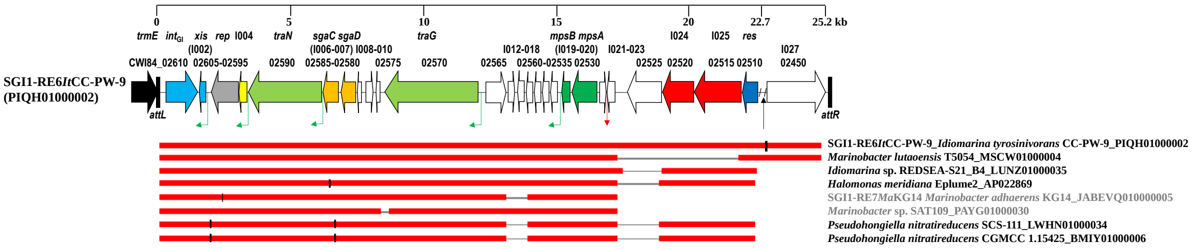

SGI1-RE6*It*CC-PW-9 *Idiomarina tyrosinivorans* CC-PW-9 (PIQH01000002): (as a reference); ORFs and genes encoding proteins of known function: **I001** (***int***GI) (integrase), **I002** (***xis***) (excisionase), **I003** (***rep***) (replication protein), **I004** (helix-turn-helix domain-containing protein), **I005** (***traN***) (conjugal transfer protein), **I006** (***sgaC***) (flagellar transcriptional activator subunit C), **I007** (***sgaD***) (flagellar transcriptional activator subunit D), **I011** (***traG***) (conjugal transfer protein), **I019** (***mpsB***) (mobilization protein MpsB), **I020** (***mpsA***) (mobilization protein MpsA), **I024** (*Bsu*B1-*Pst*1 restriction endonuclease family protein), **I025** (N-6 DNA methylase), **I026** (*res*) (resolvase).

Marinobacter lutaoensis **T5054 (MSCW01000004): I023-I025** region corresponds to 4 ORFs encoding a magnesium/cobalt transporter CorA, a FAD: protein FMN transferase, a calcium/sodium antiporter, and an undecaprenyl-diphosphate phosphatase; absence of MDR region.

Idiomarina **sp. REDSEA-S21_B4 (LUNZ01000035/12): I023** region corresponds to an IS*3* element; **I027** region corresponds to 2 ORFs encoding a chromate efflux transporter and a helix-turn-helix transcriptional regulator; absence of MDR region.

Halomonas meridiana **Eplume2 (AP022869):** insertion of an IS*1380* element **between *traN* and I006**; **I023** region corresponds to an ORF encoding an AAA family ATPase; **I027** region shares nt identity with S044; absence of MDR region.

SGI1-RE7*Ma*KG14 *Marinobacter adhaerens* KG14 (JABEVQ010000005/1) (cluster 7): insertion of an IS element of the IS*66* family with an ORF encoding a manganese efflux pump in the region **between I002 and *rep***; no significant similarity for the **I013-I014** region; **I023-I027** region corresponds to 4 ORFs encoding an AAA family ATPase, a DNA mismatch endonuclease Vsr, a DUF4928 family protein and a DNA (cytosine-5-)-methyltransferase; absence of *res*; absence of MDR region.

Marinobacter **sp. SAT109 (PAYG01000030/41) (cluster 7):** shorter region **between I009 and *traG***; **I023-I027** region corresponds to 4 ORFs encoding an AAA family ATPase truncated by an IS*L3* element, a DNA mismatch endonuclease Vsr, a DUF4928 family protein, a DNA (cytosine-5-)-methyltransferase; absence of *res*; absence of MDR region.

Pseudohongiella nitratireducens **SCS-111 (LWHN01000034/30/29):** insertion of an IS*L3*-like element **between I002 and *rep***; insertion of an IS*L3*-like element in **I006;** no significant similarity for the **I013-I014** region; **I023** region corresponds to an ORF encoding an AAA family ATPase; **I027** region shares nt identity with S044; absence of MDR region.

Pseudohongiella nitratireducens **CGMCC 1.15425 (BMIY01000006):** insertion of an IS*L3*-like element **between I002 and *rep***; insertion of an IS*L3*-like element in **I006;** no significant similarity for the **I013-I014** region; **I023** region corresponds to an ORF encoding an AAA family ATPase; **I027** region shares nt identity with S044; absence of MDR region.

Supplementary Figures 3. **BLAST alignment of the first part of the SGI1-RE backbones (*attL-oriT* segment).**

The colors of the strains correspond to the clusters of SGI1-REs: red (cluster1: SGI1 cluster), green (cluster 2: PGI1 cluster), blue (cluster 3: PGI2 cluster), purple (cluster 4: AGI1 cluster), brown (cluster 5). The characterized SGI1-REs are underlined. The ORFs of the query sequence are represented. The following colors are used for ORFs of known function:

- DNA recombination (*int*, *xis*)
- DNA replication (*rep*)
- Transcriptional regulator
- Type IV secretion system subunit (*tra*)
- Transcriptional activator
- Mobilization gene (*mps*)
- DNA replication, recombination, and repair (helicase)
- Unknown function

The green angled arrows indicate AcaCD binding sites, and the red arrow indicates *oriT*. The black vertical line represents *attL* or *attR* attachment sites

(3A) Alignment with the *alpA-oriT* segment of *Aeromonas veronii* CB51 (CP015448).

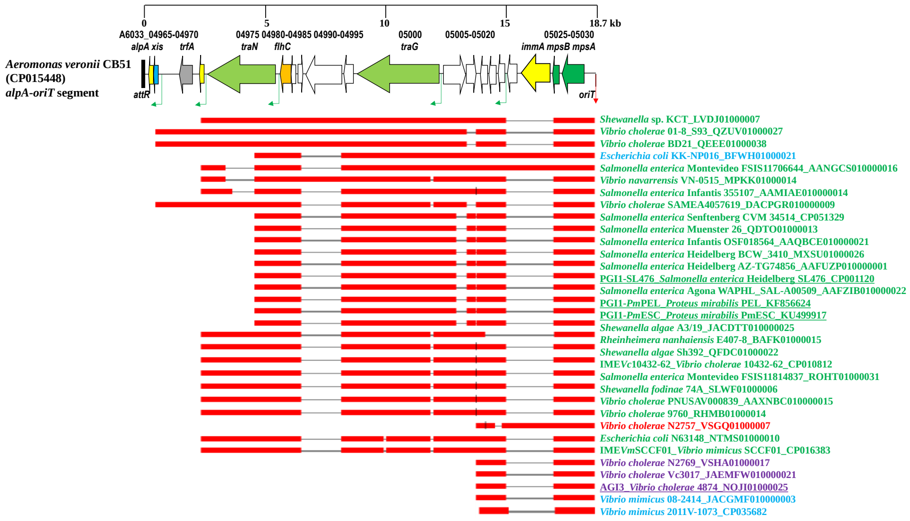

(3B) Alignment with the *alpA-oriT* segment of *Aeromonas simiae* A6 (CP040449).

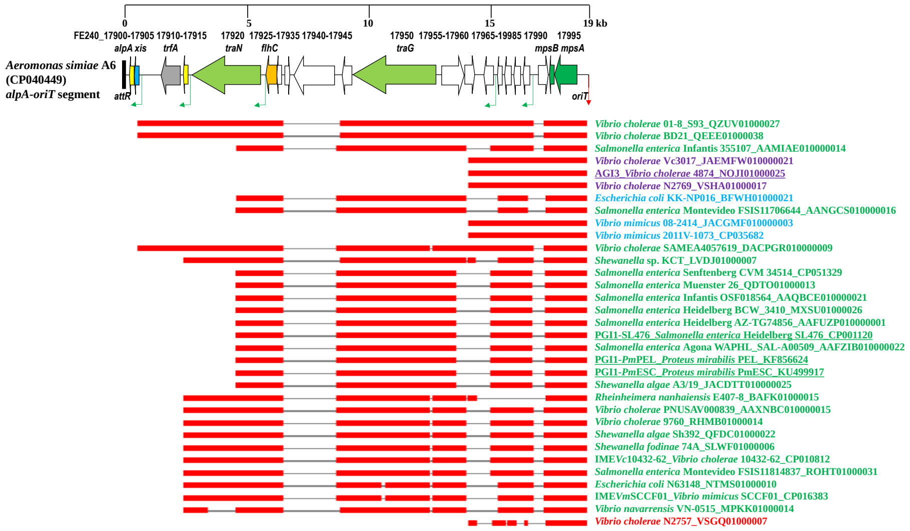

(3C) Alignment with the first part of PGI1 backbone.

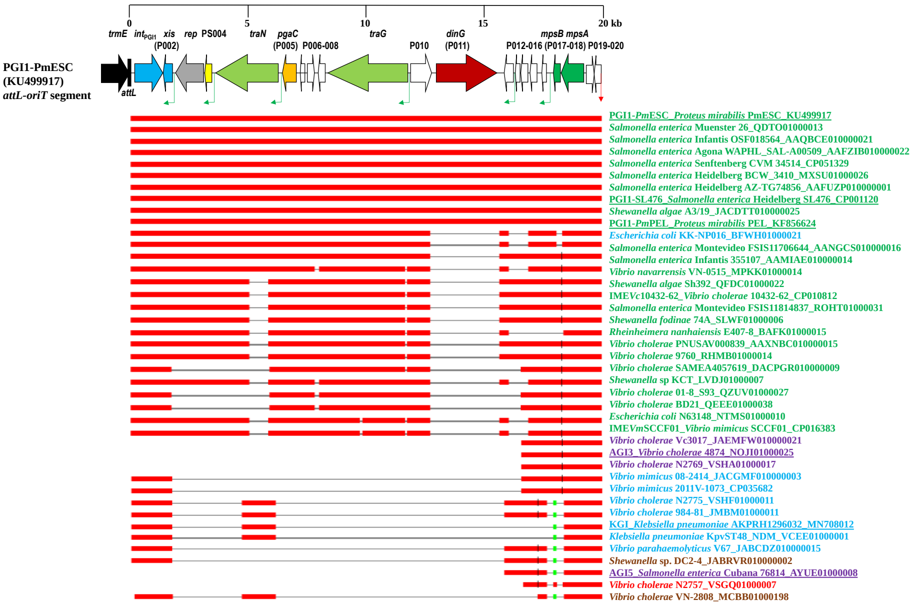

Supplementary Figures 4. BLAST alignment of the second part of the SGI1-RE backbones (*oriT*-*attR* segment) with the ORFs of known SGI1-REs.

The colors of the strains correspond to the clusters of SGI1-REs: red (cluster1: SGI1 cluster), green (cluster 2: PGI1 cluster), blue (cluster 3: PGI2 cluster), purple (cluster 4: AGI1 cluster), brown (cluster 5), black (cluster 6). The characterized SGI1-REs are underlined. The ORFs of the query sequence are represented. The following colors are used for ORFs of known function:

Toxin-antitoxin system (subtilisin-ATPase) DNA replication, recombination, and repair (helicase-endonuclease (*ybjD*)) DNA restriction/modification
Unknowm function

(4A) Alignment with the Sputw3181_4076 locus of *Shewanella* sp W3-18-1 (CP000503).

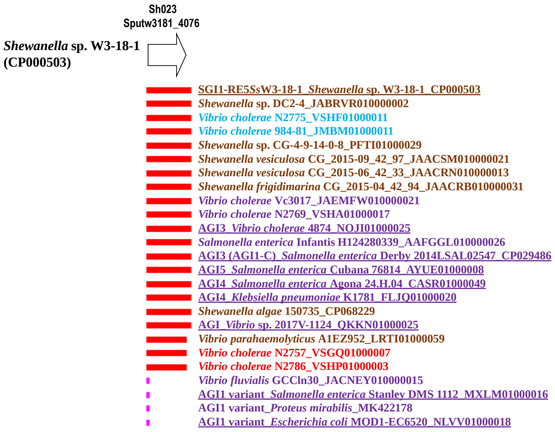

(4B) Alignment with S025-S026 (*sgiT*-*sgiA*) (encoding a subtilisin and an ATPase: TA).

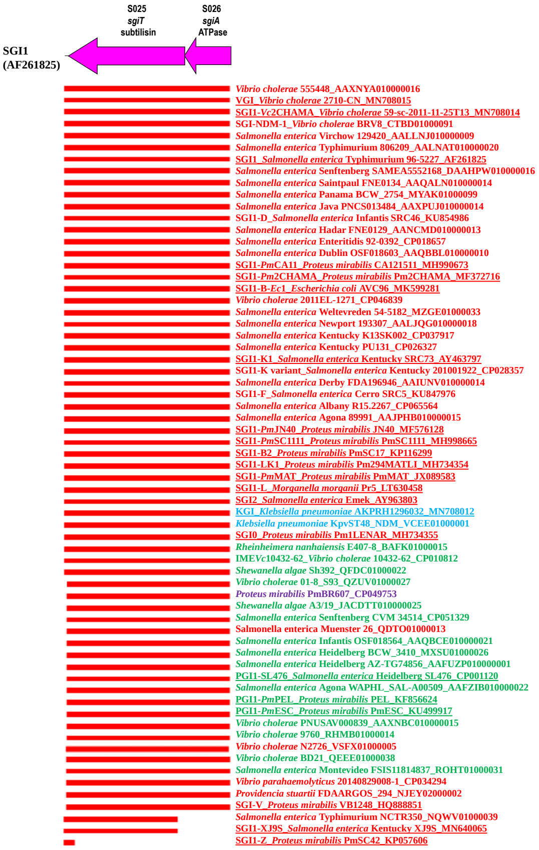

(4C) Alignment with S023-S024 (*uvrD-ybjD*) (encoding a helicase and an endonuclease).

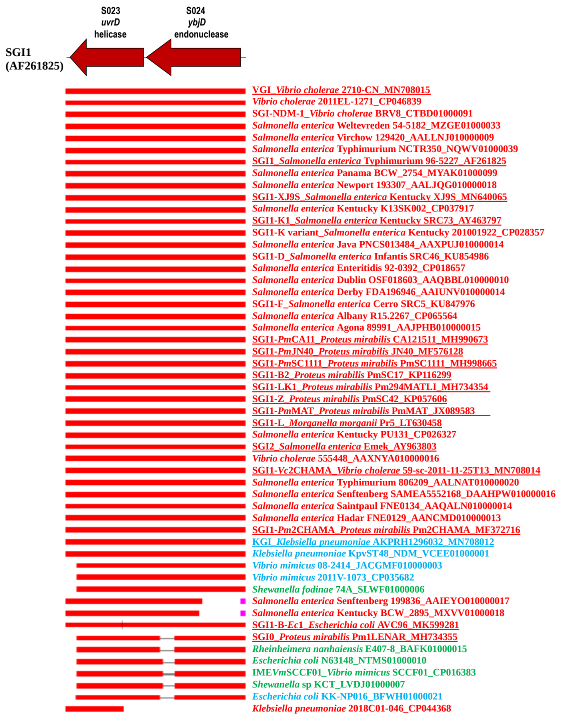

(4D) Alignment with A025-A026 (encoding a *Bsu*B1-*Pst*1 restriction endonuclease and an N-6 DNA methylase).

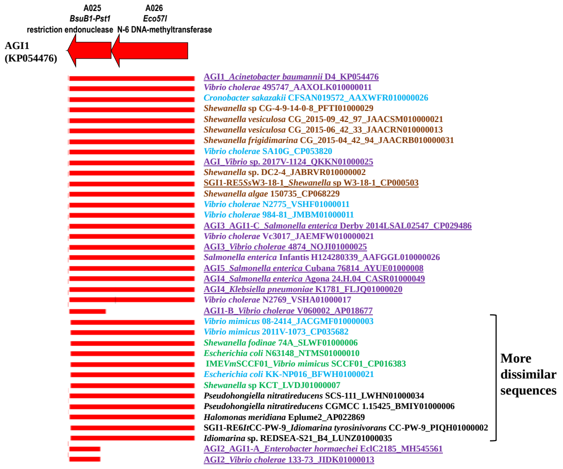

(4E) Alignment with A023-A024.

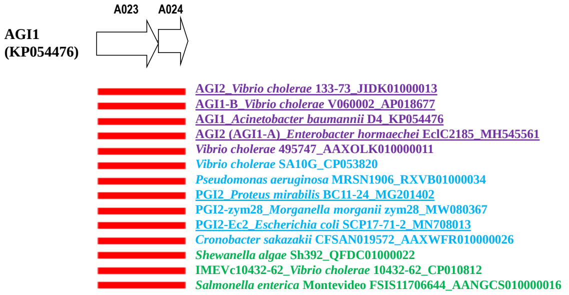

Supplementary Figures 5. BLAST alignment of the unusual ORFs of the second part of the SGI1-RE backbones (*oriT-attR* segment) with all SGI1-REs.

The colors of the strains correspond to the clusters of SGI1-REs: red (cluster1: SGI1 cluster), green (cluster 2: PGI1 cluster), blue (cluster 3: PGI2 cluster), brown (cluster 5). The ORFs of the query sequence are represented. The following colors are used for ORFs of known function:

Resolvase — DNA replication, recombination, and repair (helicase) — DNA restriction/modification — Unknown function

(5A) Alignment with the ORFs encoding a DNA-helicase, a macro domain-containing protein, two DUF4433 domain-containing proteins and a resolvase of *S.* Infantis 355107 (AAMIAE010000014).

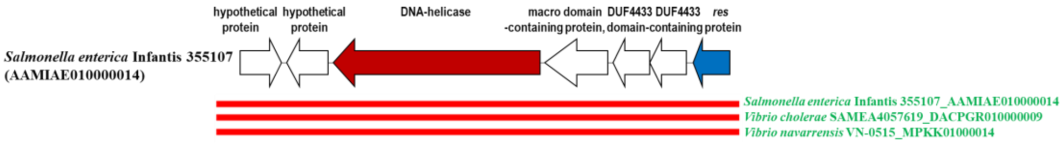

(5B) Alignment with the ORFs encoding a *Bsu*B1-*Pst*1 restriction endonuclease, an *Eco57*I restriction-modification methylase and a resolvase of IMEVmSCCF01 from *Vibrio mimicus* SCCF01 (CP016383).

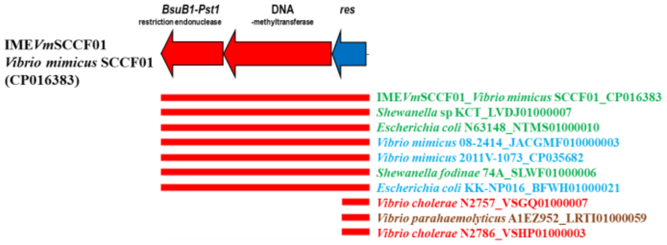

(5C) Alignment with the ORFs encoding an *Xcy*I restriction endonuclease, a DNA-methyltransferase, and a resolvase of *Vibrio cholerae* N2786 (VSHP01000003).

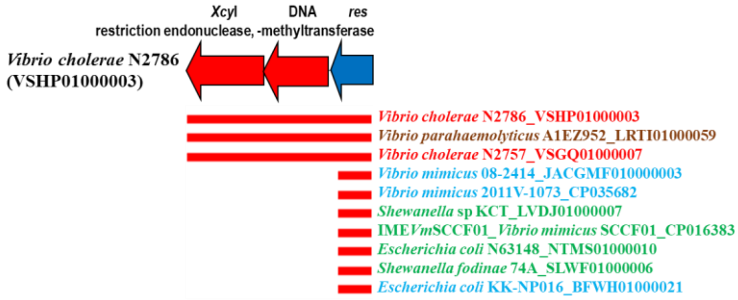

Supplementary Figure 6. **Weblogo of the *attL* and *attR* attachment sites.**

attL
(n=126)

attR
(n=125)

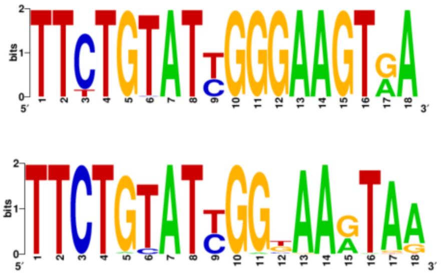

Supplementary Figure 7. **Weblogo of the AcaCD binding sites.**

AcaCD *xis*
(n=126)

AcaCD 004
(n=126)

AcaCD *traN*
(n=111)

AcaCD *traH*
(n=98)

AcaCD *traG*
(n=24)

AcaCD P012
(n=30)

AcaCD 018
(n=126)

All AcaCD
(n=641)

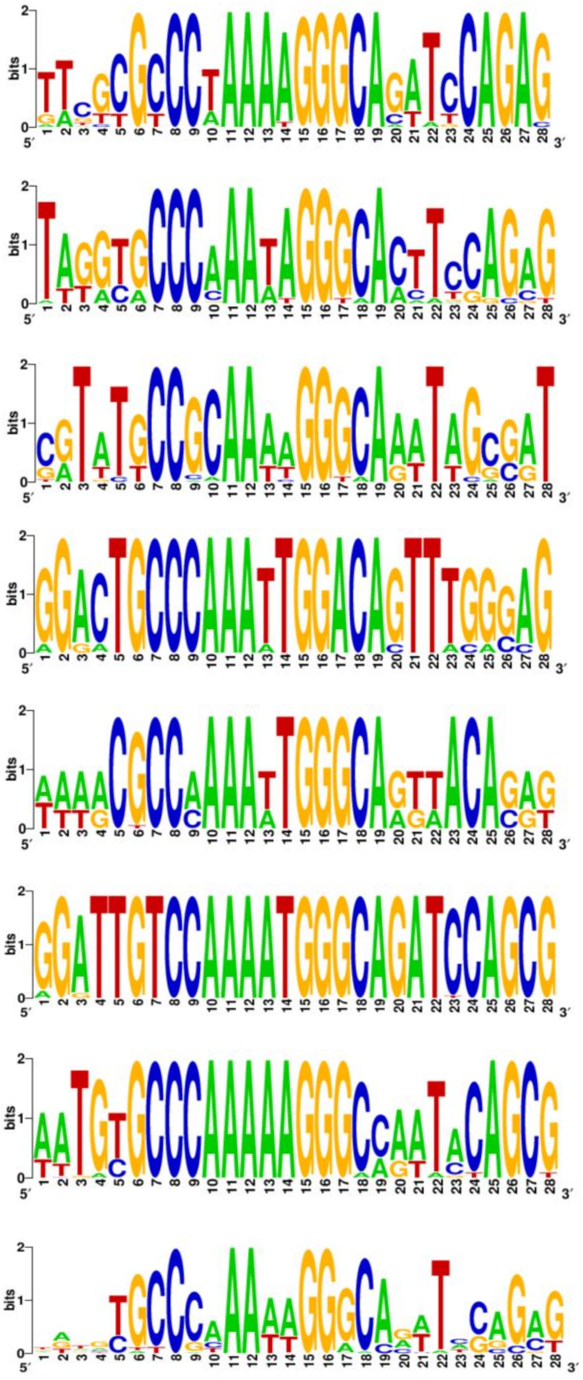

Supplement: Supplementary file 1 [file Presentation_1.pdf]
