# Supplementary material for: Overview of Salmonella Genomic Island 1-Related Elements Among Gamma-Proteobacteria Reveals Their Wide Distribution Among Environmental Species
Source: Front Microbiol. 2022 Apr 11;13:857492. doi: 10.3389/fmicb.2022.857492 (PMC9035990; doi:10.3389/fmicb.2022.857492)
Supplement: Supplementary file 2 [file Table_1.docx]

**Supplementary Table 1. Characteristics of the strains harboring the SGI1-REs analyzed in this study.**

| GI name^a^ | Strains^b^ | Accession number^c^ | Reference^d^ | Date^e^ | Country^e^ | Isolation source^e^ |
| --- | --- | --- | --- | --- | --- | --- |
| SGI1-B-*Ec*1  SGI1-L  SGI1-*Pm*MAT  SGI1-V (SGI-V)  SGI1-Z  SGI1-*Pm*2CHAMA  SGI1-LK1  SGI1-B2  SGI0  SGI1-*Pm*SC1111  SGI1-*PmJ*N40  SGI1-*Pm*CA11  SGI1-F  SGI2  SGI1-D  SGI1-K variant  SGI1-K1  SGI1-XJ9S  SGI1  SGI-NDM-1  SGI1-*Vc*2CHAMA  VGI | *Escherichia coli* AVC96  *Klebsiella pneumoniae* 2018C01-046  *Morganella morganii* Pr5  *Proteus mirabilis* PmMAT  *Proteus mirabilis* VB1248  *Proteus mirabilis* PmSC42  *Proteus mirabilis* Pm2CHAMA  *Proteus mirabilis* Pm294MATLI  *Proteus mirabilis* PmSC17  *Proteus mirabilis* Pm1LENAR  *Proteus mirabilis* PmSC1111  *Proteus mirabilis* JN40  *Proteus mirabilis* CA121511  *Providencia stuartii* FDAARGOS_294  *Salmonella enterica* Agona 89991  *Salmonella enterica* Albany R15.2267  *Salmonella enterica* Cerro SRC5  *Salmonella enterica* Derby FDA196946  *Salmonella enterica* Dublin OSF018603  *Salmonella enterica* Emek SRC19  *Salmonella enterica* Enteritidis 92-0392  *Salmonella enterica* Hadar FNE0129  *Salmonella enterica* Infantis SRC46  *Salmonella enterica* Java PNCS013484  *Salmonella enterica* Kentucky 201001922  *Salmonella enterica* Kentucky SRC73  *Salmonella enterica* Kentucky BCW_2895  *Salmonella enterica* Kentucky PU131  *Salmonella enterica* Kentucky K13SK002  *Salmonella enterica* Kentucky XJ9S  *Salmonella enterica* Newport 193307  *Salmonella enterica* Panama BCW_2754  *Salmonella enterica* Saintpaul FNE0134  *Salmonella enterica* Senftenberg 199836  *Salmonella enterica* Senftenberg SAMEA5552168  *Salmonella enterica* Typhimurium 96-5227  *Salmonella enterica* Typhimurium FSIS11815820  *Salmonella enterica* Typhimurium NCTR350  *Salmonella enterica* Typhimurium 806209  *Salmonella enterica* Virchow 129420  *Salmonella enterica* Weltevreden 54-5182  *Vibrio cholerae* BRV8  *Vibrio cholerae* N2786  *Vibrio cholerae* N2726  *Vibrio cholerae* N2757  *Vibrio cholerae* 59-sc-2011-11-25T13  *Vibrio cholerae* 2011EL-1271  *Vibrio cholerae* 2710-CN  *Vibrio cholerae* 555448  *Vibrio parahaemolyticus* 20140829008-1 | MK599281  CP044368  LT630458  JX089583  HQ888851  KP057606  MF372716  MH734354  KP116299  MH734355  MH998665  MF576128  MH990673  NJEY02000002  AAJPHB010000015  CP065564  KU847976  AAIUNV010000014  AAQBBL010000010  AY963803  CP018657  AANCMD010000013  KU854986  AAXPUJ010000014  CP028357  AY463797  MXVV01000018  CP026327  CP037917  MN640065  AALJQG010000018  MYAK01000099/4/781/962  AAQALN010000014/15  AAIEYO010000017  DAAHPW010000016  AF261825  AAHSTR010000012  NQWV01000039/27  AALNAT010000020  AALLNJ010000009  MZGE01000033/781  CTBD01000091  VSHP01000003  VSFX01000005  VSGQ01000007  MN708014  CP046839  MN708015  AAXNYA010000016  CP034294 | Cummins, 2019  Schultz, 2017  Siebor, 2013  Siebor, 2011  Lei, 2015  de Curraize, 2018  de Curraize, 2020b  Lei, 2015  de Curraize, 2020a  Wang, 2019  Bie, 2018  Xiao, 2019  (Levings, 2005)  Levings, 2008  (Levings, 2005)  (Hawkey, 2019)  Levings, 2007  Lei, 2020  Boyd, 2001  Cummins, 2020  Cummins, 2020 | 2009  2018  2013  2011  2009  2013  2013  2015  2013  2013  2016  2013  2012  Nc  2015  2015  1999  2002  2007  1999  1992  2013  2001  2017  2010  2001  Nc  2013  2013  2019  2015  2002  2013  2015  2018  Nc  2018  2005  2019  2015  Nc  2011  Nc  Nc  Nc  Nc  Nc  1986  2018  2014 | Australia  Taiwan  France  France  France  China  France  France  China  France  China  China  China  USA  United Kingdom  Taiwan  Australia  Viet Nam  USA  Australia  USA  Pakistan  Australia  Canada  Marocco  Australia  Nc  USA  South Korea  China  United Kingdom  Taiwan  Morocco  United Kingdom  Germany  Canada  USA  Egypt  United Kingdom  United Kingdom  Nc  United Kingdom (from India)  Nc  Nc  Nc  Nc  Nc  China  Nc  China | Poultry with colibacillosis  Blood  Urine sample  Bone tissue  Stool and blood cultures  Stool of pig farm  Urinary specimen  Feces  Liver of pig farm  Pus  Swine sample  Broiler carcasses  Sputum  Clinical isolate  Human sample  Stool  From a returned traveller from Thailand  Frozen scallops  Bovine (tissue)  Sewage effluent  Human sample  Licorice root (mafco root)  Clinical sample  Stool  Human sample  Spice imported from India  Nc  Human Feces  Chicken  Poultry slaughterhouse  Human sample  Human sample  Octopus  Human sample  Feed  Nc  Comminuted beef  Ground cumin seeds  Human sample  Human sample  Nc  Blood culture  Nc  Nc  Nc  Nc  Nc  Nc  Feces  AHPND infected Litopenaeus vannamei |
| PGI1-*Pm*ESC  PGI1-*Pm*PEL  PGI1-SL476  IMEVc10432-62  IME*Vm*SCCF01 | *Escherichia coli* N63148  *Proteus mirabilis* PmESC  *Proteus mirabilis* PEL  *Salmonella enterica* Agona WAPHL_SAL-A00509  *Salmonella enterica* Heidelberg SL476  *Salmonella enterica* Heidelberg AZ-TG74856  *Salmonella enterica* Heidelberg BCW_3410  *Salmonella enterica* Infantis 355107  *Salmonella enterica* Infantis OSF018564  *Salmonella enterica* Montevideo FSIS11814837  *Salmonella enterica* Montevideo FSIS11706644  *Salmonella enterica* Muenster 26  *Salmonella enterica* Senftenberg CVM 34514  *Vibrio cholerae* 9760  *Vibrio cholerae* 10432-62  *Vibrio cholerae* BD21  *Vibrio cholerae* 01-8_S93  *Vibrio cholerae* SAMEA4057619  *Vibrio cholerae* PNUSAV000839  *Vibrio mimicus* SCCF01  *Vibrio navarrensis* VN-0515  *Shewanella algae* A3/19  *Shewanella algae* Sh392  *Shewanella fodinae* 74A  *Shewanella* sp. KCT  *Rheinheimera nanhaiensis* E407-8 | NTMS01000010  KU499917  KF856624  AAFZIB010000022/24  CP001120  AAFUZP010000001  MXSU01000026/6  AAMIAE010000014  AAQBCE010000021/23  ROHT01000031  AANGCS010000016  QDTO01000013  CP051329  RHMB01000014  CP010812  QEEE01000038  QZUV01000027  DACPGR010000009  AAXNBC010000015/39  CP016383  MPKK01000014  JACDTT010000025/49  QFDC01000022/74/49  SLWF01000006  LVDJ01000007  BAFK01000015 | Siebor, 2016  Girlich, 2015  Siebor, 2014  (de Curraize, 2021)  (de Curraize, 2021) | 2015  2013  2012  2004  2003  2005  2003  2017  2007  2018  2017  2007  2004  1975  1962  2013  2001  2007  Nc  2013  2007  2019  2006  Nc  2014  Nc | USA  France  France  USA  USA  USA  USA  United Kingdom  USA  USA  USA  USA  USA  Russia  Philippines  Bangladesh  China  Thailand  USA  China  Germany  Brazil  Argentina  USA  Taiwan  China | Ground turkey  Implantable port  Urine sample  Ground turkey  Ground turkey  Ground turkey  Ground meat of Meleagris gallopavo (wild turkey)  Human sample  Camel  Animal-cattle-dairy cow  Product-raw-intact-beef  Equus ferus caballus Feces  Turkey  Cholera disease  Diarrhea  Water  Human sample  Penaeus monodon (giant tiger shrimp)  Nc  Yellow catfish  Pig  Swine pen  Skin and soft tissue infection  Freshwater and sediment post-fracking  Mollusk (common Orient clam: *Meretrix lusoria*)  Deep-sea sediment of the South China Sea (1,800 m) |
| PGI2‐*Ec*-2  KGI  PGI2-zym28  PGI2 | *Cronobacter sakazakii* CFSAN019572  *Escherichia coli* SCP17-71-2  *Escherichia coli* KK-NP016  *Klebsiella pneumoniae* KpvST48_NDM  *Klebsiella pneumoniae* AKPRH1296032  *Morganella morganii* zy_m28  *Proteus mirabilis* BC11-24  *Pseudomonas aeruginosa* MRSN1906  *Vibrio cholerae* 984-81  *Vibrio cholerae* N2775  *Vibrio cholerae* SA10G  *Vibrio mimicus* 2011V-1073  *Vibrio mimicus* 08-2414  *Vibrio parahaemolyticus* V67 | AAXWFR010000026/21  MN708013  BFWH01000021  VCEE01000001  MN708012  MW080367  MG201402  RXVB01000034/21  JMBM01000011  VSHF01000011/12  CP053820  CP035682  JACGMF010000003  JABCDZ010000015 | Cummins, 2020  Cummins, 2020  Lei, 2018 | 2014  Nc  2014  2018  Nc  2018  2016  2010  1981  Nc  2004  Nc  Nc  2015 | USA  Netherlands  Japan  United Kingdom  United Kingdom  China  China  USA  India  Nc  USA  Nc  Nc  China | Environmental swab  Human sample  Feces of Bos taurus  Rectal swab  Human sample  Pelvic cavity shunt fluid  Liver of a diarrheic piglet  Groin  Diarrhea  Nc  Water sample  Nc  Nc  Shrimp |
| AGI2 (AGI1-A)  AGI1 variant  AGI4  AGI1 variant  AGI4  AGI5  AGI3 (AGI1-C)  AGI1 variant  AGI1  AGI1-B  AGI2  AGI3  AGI | *Enterobacter hormaechei* EclC2185  *Escherichia coli* MOD1-EC6520  *Klebsiella pneumoniae* k1781  *Proteus mirabilis*  *Proteus mirabilis* PmBR607  *Salmonella enterica* Agona 24.H.04  *Salmonella enterica* Cubana 76814  *Salmonella enterica* Derby 2014LSAL02547  *Salmonella enterica* Infantis H124280339  *Salmonella enterica* Stanley DMS 1112  *Acinetobacter baumannii* D4  *Vibrio cholerae* V060002  *Vibrio cholerae* 133-73  *Vibrio cholerae* 4874  *Vibrio cholerae* N2769  *Vibrio cholerae* Vc3017  *Vibrio cholerae* 495747  *Vibrio fluvialis* GCCln30  *Vibrio* sp. 2017V-1124 | MH545561  NLVV01000018/47/28  FLJQ01000020/22  MK422178  CP049753  CASR01000049/44  AYUE01000008/66  CP029486  AAFGGL010000026/29  MXLM01000016  KP054476  AP018677  JIDK01000013  NOJI01000025/29/33  VSHA01000017/3  JAEMFW010000021  AAXOLK010000011  JACNEY010000015  QKKN01000025/42 | Siebor, 2019  Siebor, 2020  Siebor, 2020  Siebor, 2020  Siebor, 2020  Siebor, 2019  Siebor, 2020  Hamidian, 2015  Siebor, 2019  Siebor, 2020  Siebor, 2020  Siebor, 2020 | 2012  1990  2008  Nc  2015  2004  2004  2014  2012  1988  2006  1997  1973  2012  Nc  2018  2017  2018  2017 | France  India  United Kingdom  China  Brazil  China  USA  France  United Kingdom  United Kingdom  Australia  Japan (from Indonesia)  India  Bangladesh  Nc  China  England  Bangladesh  USA | Urine  Blood from *Gallus gallus domesticus*  Blood  Chicken  Urine  Human sample  Nc  Meat from pig – fresh  Human sample  Nc  Wound sample from patient  Human sample  Diarrhea  Water  Nc  Shrimp  Gastrointestinal sample  Stool  Wound |
| SGI1-RE5*Ss*W3-18-1 | *Vibrio cholerae* VN-2808  *Vibrio parahaemolyticus* A1EZ952  *Shewanella algae* 150735  *Shewanella frigidimarina* CG_2015-04_42_94  *Shewanella vesiculosa* CG_2015-06_42_33  *Shewanella vesiculosa* CG_2015-09_42_97  *Shewanella* sp. CG_4_9_14_0_8  *Shewanella* sp. W3-18-1  *Shewanella* sp. DC2-4 | MCBB01000198/211/210/160  LRTI01000059  CP068229  JAACRB010000031  JAACRN010000013  JAACSM010000021/26  PFTI01000029  CP000503  JABRVR010000002 | Siebor, 2020 | 2014  2001  2015  2015  2015  2015  2014  Nc  2019 | Germany  Canada  Spain  USA  USA  USA  USA  USA  USA | Costal water (North Sea)  Gastroenteritis clinical sample  Human wound  Subsurface aquifer (Crystal Geyser borehole)  Subsurface aquifer (Crystal Geyser borehole)  Subsurface aquifer (Crystal Geyser borehole)  Groundwater (Crystal Geyser borehole)  Pacific Ocean marine sediments (630 m)  Acid mine drainage |
| SGI1-RE6*It*CC-PW-9 | *Halomonas meridiana* Eplume2  *Idiomarina tyrosinivorans* CC-PW-9  *Idiomarina* sp. REDSEA-S21_B4  *Marinobacter lutaoensis* T5054  *Pseudohongiella nitratireducens* CGMCC 1.15425  *Pseudohongiella nitratireducens* SCS-111 | AP022869  PIQH01000002  LUNZ01000035/12  MSCW01000004  BMIY01000006  LWHN01000034/30/29 |  | 1995  Nc  2011  2003  Nc  2014 | Japan  China  Saudi Arabia  Taiwan  Nc  South China Sea | hydrothermal-plume, north east Pacific Ocean (2,000 m)  Seawater  Red Sea water column Station 91 – depth 500 m  Hot spring  Nc  sea water (450 m water depth) northern South China Sea |
| SGI1-RE7MaKG14 | *Marinobacter adhaerens* KG14  *Marinobacter* sp. SAT109 | JABEVQ010000005/1  PAYG01000030/41 |  | 2018  2009 | South Korea  Mediterranean Sea | Fermentation of ganjang, Korean traditional soy sauce  Marine water sample |

^a^ The SGI1-REs used as a reference in each cluster are underlined.

^b^ Clusters (n=7) are based on the homology of SGI1-REs integrase gene (nt homology >97%, corresponding to AA homology >98%).

^c^ Multiple accession numbers are shown for backbones in multiple unassembled contigs

^d^ References for backbones not described in detail are included in parentheses

^e^ Nc: Not collected

**Supplementary Table 2.** **Characteristics of the backbones of the SGI1-REs analyzed in this study and position of MDR regions.**

| GI name | Strains | Size  (bp) | G+C% | MDR region^a^  (insertion site)^b^ | *attL* attachment sites^i^ | *attR* attachment sites^i^ |
| --- | --- | --- | --- | --- | --- | --- |
| SGI1-B-*Ec*1  SGI1-L  SGI1-*Pm*MAT  SGI1-V (SGI-V)  SGI1-Z  SGI1-*Pm*2CHAMA  SGI1-LK1  SGI1-B2  SGI0  SGI1-*Pm*SC1111  SGI1-PmJN40  SGI1-PmCA11  SGI1-F  SGI2  SGI1-D  SGI1-K variant  SGI1-K1  SGI1  SGI-NDM-1  SGI1‐*Vc*2CHAMA  VGI | *Escherichia coli* AVC96  *Klebsiella pneumoniae* 2018C01-046  *Morganella morganii* Pr5  *Proteus mirabilis* PmMAT  *Proteus mirabilis* VB1248  *Proteus mirabilis* PmSC42  *Proteus mirabilis* Pm2CHAMA  *Proteus mirabilis* Pm294MATLI  *Proteus mirabilis* PmSC17  *Proteus mirabilis* Pm1LENAR  *Proteus mirabilis* PmSC1111  *Proteus mirabilis* JN40  *Proteus mirabilis* CA121511  *Providencia stuartii* FDAARGOS_294  *Salmonella enterica* Agona 89991  *Salmonella enterica* Albany R15.2267  *Salmonella enterica* Cerro SRC5  *Salmonella enterica* Derby FDA196946  *Salmonella enterica* Dublin OSF018603  *Salmonella enterica* Emek SRC19  *Salmonella enterica* Enteritidis 92-0392  *Salmonella enterica* Hadar FNE0129  *Salmonella enterica* Infantis SRC46  *Salmonella enterica* Java PNCS013484  *Salmonella enterica* Kentucky 201001922  *Salmonella enterica* Kentucky SRC73  *Salmonella enterica* Kentucky BCW_2895  *Salmonella enterica* Kentucky PU131  *Salmonella enterica* Kentucky K13SK002  *Salmonella enterica* Kentucky XJ9S  *Salmonella enterica* Newport 193307  *Salmonella enterica* Panama BCW_2754  *Salmonella enterica* Saintpaul FNE0134  *Salmonella enterica* Senftenberg 199836  *Salmonella enterica* Senftenberg SAMEA5552168  *Salmonella enterica* Typhimurium 96-5227  *Salmonella enterica* Typhimurium FSIS11815820  *Salmonella enterica* Typhimurium NCTR350  *Salmonella enterica* Typhimurium 806209  *Salmonella enterica* Virchow 129420  *Salmonella enterica* Weltevreden 54-5182  *Vibrio cholerae* BRV8  *Vibrio cholerae* N2786  *Vibrio cholerae* N2726  *Vibrio cholerae* N2757  *Vibrio cholerae* 59-sc-2011-11-25T13  *Vibrio cholerae* 2011EL-1271  *Vibrio cholerae* 2710-CN  *Vibrio cholerae* 555448  *Vibrio parahaemolyticus* 20140829008-1 | 29915  20259  25848  24583  25774  23265  27194  20051  27365  27297  28625  27365  27365  25774  27365  27365  27365  27364  27365  27364  27365  17436  27365  27365  25416  25416  20101  24942  25141  23114  25844  27365  27193  22119  27364  27366  19213  23561  17436  26694  25141  27365  22605  26324  16505  27194  27365  27396  27364  26435 | 44.74  44.82  44.11  44.13  43.97  44.71  44.04  44.13  43.96  43.88  43.93  43.96  43.96  43.97  43.96  43.95  43.95  43.96  43.96  44.03  43.96  43.92  43.96  43.96  44.16  44.16  44.97  44.16  44.19  44.52  44.12  43.96  44.05  44.66  43.96  43.97  44.53  44.44  43.92  43.97  44.19  43.96  43.95  43.78  44.17  44.04  43.96  43.97  43.96  43.98 | P (ACTTG)  P (nd)  P (ACTTG)  P (ACTTG)  P (AAATT)^c^  P (ACTTG)^d^  P (AACTT)^c^  P (ACTTG)  P (ACTTG)  -  P (ACTTG)  P (ACTTG)  P (ACTTG)  P (AAATT)^c^  P (ACTTG)  P (ACTTG)  P (ACTTG)  P (ACTTG)  P (ACTTG)  P (CCATG)^e^  P (ACTTG)  P (ACTTG)  P (ACTTG)  P (ACTTG)  P (ACTTG)^c^  P (ACTTG)^c^  P (nd)  P (nd)  P (nd)  P (nd)  P (ACTTG)  P (ACTTG)  P (AACTT)^c^  P (ACTTG)^d^  P (ACTTG)  P (ACTTG)  P (ACTTG)^d^  P (nd)  P (ACTTG)  P (nd)  P (nd)  P (ACTTG)  -  -  -  P (AACTT)^c^  P (ACTTG)  -  P (ACTTG)  - | TTCTGTATT**GGGAAGTAA**  TTCTGTATT**GGGAAGTAA**  TTTTGTATC**GGGAAGTAA**  TTCTGTATT**GGGAAGTAA**  TTCTGTATT**GGGAAGTAA**  TTCTGTATC**GGGAAGTAA**  TTCTGTATC**GGGAAGTAA**  TTCTGTATT**GGGAAGTAA**  TTCTGTATC**GGGAAGTAA**  TTCTGTATT**GGGAAGTAA**  TTCTGTATT**GGGAAGTAA**  TTCTGTATT**GGGAAGTAA**  TTCTGTATT**GGGAAGTAA**  TTCTGTATT**GGGAAGTAA**  TTCTGTATC**GGGAAGTAA**  TTCTGTATT**GGGAAGTAA**  TTCTGTATT**GGGAAGTAA**  TTCTGTATT**GGGAAGTAA**  TTCTGTATT**GGGAAGTAA**  TTCTGTATC**GGGAAGTAA**  TTCTGTATT**GGGAAGTAA**  TTCTGTATC**GGGAAGTAA**  TTCTGTATT**GGGAAGTAA**  TTCTGTATT**GGGAAGTAA**  TTCTGTATT**GGGAAGTAA**  TTCTGTATT**GGGAAGTAA**  TTCTGTATT**GGGAAGTAA**  TTCTGTATT**GGGAAGTAA**  TTCTGTATT**GGGAAGTAA**  TTCTGTATT**GGGAAGTAA**  TTCTGTATT**GGGAAGTAA**  TTCTGTATT**GGGAAGTAA**  TTCTGTATC**GGGAAGTAA**  TTCTGTATT**GGGAAGTAA**  TTCTGTATT**GGGAAGTAA**  TTCTGTATT**GGGAAGTAA**  TTCTGTATT**GGGAAGTAA**  TTCTGTATT**GGGAAGTAA**  TTCTGTATC**GGGAAGTAA**  TTCTGTATT**GGGAAGTAA**  TTCTGTATT**GGGAAGTAA**  TTCTGTATT**GGGAAGTAA**  TTCTGTATC**GGGAAGTAA**  TTCTGTATT**GGGAAGTAA**  TTCTGTATC**GGGAAGTAA**  TTCTGTATT**GGGAAGTAA**  TTCTGTATT**GGGAAGTAA**  TTCTGTATT**GGGAAGTAA**  TTCTGTATT**GGGAAGTAA**  TTCTGTATC**GGGAAGTAA** | TTCTGTATT**GGTAAGTAA**  TTCTGTATC**GGTAAGTAA**  TTCTGTATT**GGGAAGTAA**  TTCTGTATT**GGGAAGTAA**  TTCTGTATT**GGTAAGTAA**  TTCTGTATT**GGGAAGTAA**  TTCTGTATT**GGGAAGTAA**  TTCTGTATT**GGGAAGTAA**  TTCTGTATT**GGGAAGTAA**  TTCTGTATT**GGGAAGTAA**  TTCTGTATC**GGGAAGTAA**  TTCTGTATT**GGGAAGTGA**  TTCTGTATT**GGGAAGTAA**  TTCTGTATC**GGCAAGTAA**  TTCTGTATT**GGTAAGTAA**  TTCTGTATT**GGTAAGTAA**  TTCTGTATT**GGTAAGTAA**  TTCTGTATT**GGTAAGTAA**  TTCTGTATC**GGTAAGTAA**  TTCTGTATT**GGCAAATAA**  TTCTGTATC**GGTAAGTAA**  TTCTGTATC**GGCAAATAA**  TTCTGTATC**GGTAAGTAA**  TTCTGTATT**GGTAAGTAA**  TTCTGTATT**GGTAAGTAA**  TTCTGTATT**GGTAAGTAA**  TTCTGTATT**GGTAAGTAA**  TTCTGTATT**GGTAAGTAA**  TTCTGTATT**GGTAAGTAA**  Deleted  TTCTGTATC**GGCAAATAA**  TTCTGTATC**GGTAAGTAA**  TTCTGTATC**GGCAAATAA**  TTCTGTATC**GGTAAGTAA**  TTCTGTATC**GGTAAGTAA**  TTCTGTATC**GGTAAGTAA**  TTCTGTATC**GGTAAGTAA**  TTCTGTATT**GGTAAGTAA**  TTCTGTATC**GGCAAATAA**  TTCTGTATC**GGCAAATAA**  TTCTGTATT**GGTAAGTAA**  TTCTGTATT**GGGAAATAG**  TTCTGTATT**GGGAAATAG**  TTCTTTATT**GGGAAATAG**  TTCTGTATC**GGGAAATAG**  TTCTGTATT**GGGAAATAG**  TTCTGTATT**GGGAAATAG**  TTCTGTATT**GGTAAGTAA**  TTCTGTATC**GGGAAGTAA**  TTCTGTATC**GGTAAATAA** |
| PGI1-*Pm*ESC  PGI1-*Pm*PEL  PGI1-SL476  IMEVc10432-62  IMEVmSCCF01 | *Escherichia coli* N63148  *Proteus mirabilis* PmESC  *Proteus mirabilis* PEL  *Salmonella enterica* Agona WAPHL_SAL-A00509  *Salmonella enterica* Heidelberg SL476  *Salmonella enterica* Heidelberg AZ-TG74856  *Salmonella enterica* Heidelberg BCW_3410  *Salmonella enterica* Infantis 355107  *Salmonella enterica* Infantis OSF018564  *Salmonella enterica* Montevideo FSIS11814837  *Salmonella enterica* Montevideo FSIS11706644  *Salmonella enterica* Muenster 26  *Salmonella enterica* Senftenberg CVM 34514  *Vibrio cholerae* 9760  *Vibrio cholerae* 10432-62  *Vibrio cholerae* BD21  *Vibrio cholerae* 01-8_S93  *Vibrio cholerae* SAMEA4057619  *Vibrio cholerae* PNUSAV000839  *Vibrio mimicus* SCCF01  *Vibrio navarrensis* VN-0515  *Shewanella algae* A3/19  *Shewanella algae* Sh392  *Shewanella fodinae* 74A  *Shewanella* sp. KCT  *Rheinheimera nanhaiensis* E407-8 | 28120  25651  25672  25651  25651  25413  25357  27222  25651  24707  21371  25651  25340  24707  26628  24780  24780  28896  24707  27191  29287  26479  26627  28034  27124  27168 | 48.35  47.00  47.02  47.01  47.00  47.08  47.06  48.89  47.01  47.92  46.36  47.01  47.07  47.88  46.61  47.14  47.13  49.46  47.84  48.34  48.21  47.17  46.63  48.27  47.59  47.09 | -  P (AAATT)  P (AAATT)  P (AAATT)  P (AAATT)  P (AAATT)^c^  P (AAATT)^d^  -  P (AAATT)  -  -  P (AAATT)  P (AAATT)^c^  -  -  -  -  -  -  -  -  -  -  P (ATGAC)  -  - | TTCTGTATT**GGGAAGTGA**  TTCTGTATT**GGGAAGTGA**  TTCTGTATT**GGGAAGTGA**  TTCTGTATT**GGGAAGTGA**  TTTTGTATT**GGGAAGTGA**  TTTTGTATT**GGGAAGTGA**  TTTTGTATT**GGGAAGTGA**  TTCTGTATT**GGGAAGTGA**  TTCTGTATT**GGGAAGTGA**  TTCTGTATC**GGGAAGTGA**  TTCTGTATC**GGGAAGTGA**  TTCTGTATT**GGGAAGTGA**  TTCTGTATT**GGGAAGTGA**  TTCTGTATT**GGGAAGTGA**  TTCTGTATC**GGGAAGTGA**  TTCTGTATT**GGGAAGTGA**  TTCTGTATT**GGGAAGTGA**  TTCTGTATT**GGGAAGTGA**  TTCTGTATT**GGGAAGTGA**  TTCTGTATC**GGGAAGTGA**  TTTTGTATT**GGGAAGTGA**  TTCTGTATC**GGGAAGTGA**  TTCTGTATC**GGGAAGTGA**  TTCTGTATC**GGGAAGTGA**  TTCTGTATC**GGGAAGTGA**  TTTTGTATC**GGGAAGTGA** | TTCTGTATT**GGTAAGTAA**  TTCTGTATT**GGGAAGTAA**  TTCTGTATT**GGGAAGTAA**  TTCTGTATT**GGTAAGTAA**  TTCTATATT**GATAACTGT***  TTCTATATT**GATAACTGT***  TTCTATATT**GATAACTGT***  TTCTGTATT**GGTAAGTAA**  TTCTGTATT**GGTAAGTAA**  TTCTGTATC**GGTAAGTAA**  TTCTGTATT**GGTAAGTAA**  TTCTGTATC**GGCAAATAA**  TTCTGTATT**GGTAAGTAA**  TTCTGTATC**GGGAAATAG**  TTCTGTATT**GGGGAAATAG**  TTCTGTATT**GGGAAATAG**  TTCTGTATT**GGGAAATAG**  TTCTGTATT**GGGAAATAG**  TTCTGTATT**GGGAAATAG**  TTCTGTATT**GGTAAATAA**  TTCTGTATT**GGCAAGTAA**  TTCTGTATC**GGGAAGTAA**  TTCTGTATC**GGGAAGTAA**  TTCTGTATC**GGGAAGTAA**  TTCTGTATC**GGTAAATAA**  TTCTGTATC**GGGAAGTAA** |
| PGI2‐*Ec-*2  KGI  PGI2-zym28  PGI2 | *Cronobacter sakazakii* CFSAN019572  *Escherichia coli* SCP17-71-2  *Escherichia coli* KK-NP016  *Klebsiella pneumoniae* KpvST48_NDM  *Klebsiella pneumoniae* AKPRH1296032  *Morganella morganii* zy_m28  *Proteus mirabilis* BC11-24  *Pseudomonas aeruginosa* MRSN1906  *Vibrio cholerae* 984-81  *Vibrio cholerae* N2775  *Vibrio cholerae* SA10G  *Vibrio mimicus* 2011V-1073  *Vibrio mimicus* 08-2414  *Vibrio parahaemolyticus* V67 | 27175  23847  26756  27238  27241  23848  23847  23848  24537  24537  24612  28120  28120  24882 | 43.32  43.23  47.06  44.00  44.00  43.23  43.23  43.22  44.09  44.09  43.58  45.53  45.53  44.70 | P (catag)^c^  P (nd)  -^f^  -^f^  -^f^  P (nd)  P (nd)  P (nd)  P (catag)^f^  P (CATAG)^f^  -^f^  -^f^  -^f^  -^g^ | TTCTGTATC**GGGAAGTGA**  TTCTGTATT**GGGAAGTGA**  TTCTGTATT**GGGAAGTGA**  TTCTGTATC**GGGAAGTGA**  TTCTGTATC**GGGAAGTGA**  TTTTGTATT**GGGAAGTGA**  TTCTGTATT**GGGAAGTGA**  TTCTGTATC**GGGAAGTGA**  TTCTGTATC**GGGAAGTGA**  TTCTGTATC**GGGAAGTGA**  TTCTGTATT**GGGAAGTGA**  TTCTGTATC**GGGAAGTAA**  TTCTGTATC**GGGAAGTAA**  TTCTGTATC**GGGAAGTGA** | TTCTGTATT**GGTAAGTAA**  TTCTGCATC**GGTAAGTAA**  TTCTGTATC**GGTAAGTAA**  TTCTGCATC**GGCAAGTAA**  TTCTGCATC**GGCAAGTAA**  TTCTGTATC**GGGAAGTAA**  TTCTGTATT**GGGAAGTAA**  TTCTGCATC**GGCAAGTAA**  TTCTGTATT**GGGAAATAG**  TTCTGTATT**GGGAAATAG**  TTCTGTATT**GGGGAAATAG**  TTCTGTATC**GGTAAATAG**  TTCTGTATC**GGTAAATAG**  TTCTGTATC**GGTAAATAA** |
| AGI2 (AGI1-A)  AGI1 variant  AGI4  AGI1 variant  AGI4  AGI5  AGI3 (AGI1-C)  AGI1 variant  AGI1  AGI1-B  AGI2  AGI3  AGI | *Enterobacter hormaechei* EclC2185  *Escherichia coli* MOD1-EC6520  *Klebsiella pneumoniae* k1781  *Proteus mirabilis*  *Proteus mirabilis* PmBR607  *Salmonella enterica* Agona 24.H.04  *Salmonella enterica* Cubana 76814  *Salmonella enterica* Derby 2014LSAL02547  *Salmonella enterica* Infantis H124280339  *Salmonella enterica* Stanley DMS 1112  *Acinetobacter baumannii* D4  *Vibrio cholerae* V060002  *Vibrio cholerae* 133-73  *Vibrio cholerae* 4874  *Vibrio cholerae* N2769  *Vibrio cholerae* Vc3017  *Vibrio cholerae* 495747  *Vibrio fluvialis* GCCln30  *Vibrio* sp. 2017V-1124 | 22019  19828  24011  19831  23933  24011  24830  24032  24011  19831  25045  22147  22017  25623  26882  25624  24950  19831  24053 | 42.90  44.06  43.84  44.09  43.26  43.83  43.93  43.78  43.83  44.09  43.01  42.91  42.90  44.49  44.53  44.63  43.05  44.10  43.93 | P (CCATA)^d^  P (ATAGG)^d^  P (TAGGT)  P (ATAGG)^d^  P (AAATT/ATAGG)^h^  P (TAGGT)  P (TGCAC)  P (CATAG)  P (TAGGT)  P (ATAGG)^d^  P (ATAGG)  P (ATAGG)^d^  P (CCATA)^d^  P (CATAG)  P (CCATA)  P (CATAG)  P (ATCGT)^e^  P (ATAGG)^d^  - | TTCTGCATT**GGGAAGTGA**  TTCTGTATT**GGGAAGTGA**  TTCTGTATC**GGGAAGTGA**  TTCTGTATT**GGGAAGTGA**  TTCTGTATT**GGGAAGTGA**  TTCTGTATT**GGGAAGTGA**  TTCTGTATT**GGGAAGTGA**  TTCTGTATT**GGGAAGTGA**  TTCTGTATT**GGGAAGTGA**  TTCTGTATT**GGGAAGTGA**  TTCTGTATC**GGGAAGTGA**  TTCTGTATC**GGGAAGTGA**  TTCTGTATT**GGGAAGTGA**  TTCTGTATT**GGGAAGTGA**  TTCTGTATC**GGGAAGTGA**  TTCTGTATT**GGGAAGTGA**  TTCTGTATC**GGGAAGTGA**  TTCTGTATC**GGGAAGTGA**  TTCTGTATT**GGGAAGTGA** | TTCTGTATC**GGTAAGTAA**  TTCTGTATT**GGTAAGTAA**  TTCTGTATT**GGCAAGTAA**  TTCTGTATT**GGGAAGTAA**  TTCTGTATT**GGGAAGTAA**  TTCTGTATT**GGTAAGTAA**  TTCTGTATC**GGTAAGTAA**  TTCTGCATC**GGTAAGTAA**  TTCTGTATC**GGTAAGTAA**  TTCTGTATT**GGTAAATAA**  TTCTGTATC**GGAAAATAA**  TTCTGTATC**GGGAAATAG**  TTCTGTATT**GGGAAATAG**  TTCTGTATT**GGGAAATAG**  TTCTGTATT**GGGAAATAG**  TTCTGTATT**GGGAAATAG**  TTCTGTATT**GGGAAATAG**  TTCTGTATT**GGGAAGTAA**  TTCTGTATT**GGGAAATAG** |
| SGI1-RE5SsW3-18-1 | *Vibrio cholerae* VN-2808  *Vibrio parahaemolyticus* A1EZ952  *Shewanella algae* 150735  *Shewanella frigidimarina* CG_2015-04_42_94  *Shewanella vesiculosa* CG_2015-06_42_33  *Shewanella vesiculosa* CG_2015-09_42_97  *Shewanella* sp CG_4_9_14_0_8  *Shewanella* sp W3-18-1  *Shewanella* sp. DC2-4 | 26244  22661  23652  23698  23698  23640  23698  23725  26231 | 44.38  43.96  44.08  44.03  44.03  44.04  44.03  43.97  43.52 | -^g^  -  -  -  -  -  -  -  - | TTCTGTATT**GGGAAGTGA**  TTCTGTATC**GGGAAGTGA**  TTCTGTATC**GGGAAGTGA**  TTTTGTATC**GGGAAGTGA**  TTTTGTATC**GGGAAGTGA**  TTTTGTATC**GGGAAGTGA**  TTTTGTATC**GGGAAGTGA**  TTCTGTATC**GGGAAGTGA**  TTCTGTATC**GGGAAGTGA** | TTCTGTATT**GGGAAATAG**  TTCTGTATC**GGTAAATAA**  TTCTGTATC**GGGAAATAA**  TTCTGTATC**GGAAAATAA**  TTCTGTATC**GGAAAATAA**  TTCTGTATC**GGAAAATAA**  TTCTGTATC**GGAAAATAA**  TTCTGTATC**GGGAAGTAA**  TTCTGTATC**GGAAAATAA** |
| SGI1-RE6*It*CC-PW-9 | *Halomonas meridiana* Eplume2  *Idiomarina tyrosinivorans* CC-PW-9  *Idiomarina* sp. REDSEA-S21_B4  *Marinobacter lutaoensis* T5054  *Pseudohongiella nitratireducens* CGMCC 1.15425  *Pseudohongiella nitratireducens* SCS-111 | 26575  25214  24547  24985  30521  30708 | 50.37  47.83  49.96  49.13  50.51  50.45 | -  P (aaatg)  -  -  -  - | TTCTGTATC**GGGAAGTGA**  TTTTGTATC**GGGAAGTGA**  TTCTGTATC**GGGAAGTGA**  TTCTGTATC**GGGAAGTGA**  TTTTGTATC**GGGAAGTGA**  TTTTGTATC**GGGAAGTGA** | TTCTGCATC**GGCAAATAA**  ttctgcatc**gggaagtga**  TTCTGTATC**GGTAAATAA**  TTCTGCATC**GGGAAGTAA**  TTCTGCATC**GGAAAATAA**  TTCTGCATC**GGAAAATAA** |
| SGI1-RE7MaKG14 | *Marinobacter adhaerens* KG14  *Marinobacter* sp. SAT109 | 25752  26314 | 50.29  49.35 | -^g^  -^g^ | TTTTGTATT**GGGAAGTAA**  TTCTGCATC**GGGAAGTGA** | TTCTGTATC**GGGAAGTAA**  TTCTGCATC**GGCAAATAA** |

The SGI1-REs used as a reference in each cluster are underlined and clusters (n=7) are based on the homology of SGI1-REs integrase.

^a^ P: present (with 5-bp duplication flanking the MDR region)

^b^ nd: not determinable, due to the loss of a part of the backbone on each side of the MDR region

^c^ 5-bp on the left side of the MDR region (because of loss of part of the backbone on the right side of the MDR region)

^d^ 5-bp on the right side of the MDR region (because of loss of part of the backbone on the left side of the MDR region)

^e^ MDR region inserted at a different position into the backbone, not upstream of the *res* gene

^f^ presence of a *res* gene in PGI2 cluster

^g^ absence of *res* gene and MDR region

^h^ MDR region inserted between the *res* gene of PGI1 and A028

^i^ Attachment sites of 18-bp and their last 9-bp in bold and underlined.

**Supplementary Tables 3. Percent Identity Matrix created by Clustal Omega for integrase genes.**

**(3A)** *int* genes of SGI1-REs used as a reference in each cluster.

| *int*_GI_ (% nt identity) | SGI1 | IMESsW3-18-1 | PGI2 | PGI1-*Pm*ESC | IMEItCC-PW-9 | IMEMaKG14 | AGI1 |
| --- | --- | --- | --- | --- | --- | --- | --- |
| SGI1 | 100.00 | 94.99 | 81.43 | 79.57 | 72.82 | 71.66 | 64.82 |
| SGI1-RE5*Ss*W3-18-1 | 94.99 | 100.00 | 82.09 | 79.90 | 73.06 | 72.16 | 64.82 |
| PGI2 | 81.43 | 82.09 | 100.00 | 94.98 | 75.16 | 74.51 | 63.70 |
| PGI1-*Pm*ESC | 79.57 | 79.90 | 94.98 | 100.00 | 76.73 | 75.08 | 63.53 |
| SGI1-RE6*It*CC-PW-9 | 72.82 | 73.06 | 75.16 | 76.73 | 100.00 | 89.59 | 62.32 |
| SGI1-RE7*Ma*KG14 | 71.66 | 72.16 | 74.51 | 75.08 | 89.59 | 100.00 | 62.23 |
| AGI1 | 64.82 | 64.82 | 63.70 | 63.53 | 62.32 | 62.23 | 100.00 |

**(3B)** *int* genes of SGI1-REs from the SGI1 cluster (cluster 1)**.**

| *int*_GI_ (% nt identity) | SGI1 | *Kp* 2018C01-046 | *Se* Panama BCW_2754 | *Vc* N2786 | *Vp* 20140829008-1 | SGI1-V | SGI0 |
| --- | --- | --- | --- | --- | --- | --- | --- |
| SGI1-B-Ec1 *Escherichia coli* AVC96  SGI1-L *Morganella morganii* Pr5  SGI1-*Pm*MAT *Proteus mirabilis* PmMAT  SGI1-Z *Proteus mirabilis* PmSC42  SGI1-*Pm*2CHAMA *Proteus mirabilis* Pm2CHAMA  SGI1-LK1 *Proteus mirabilis* Pm294MATLI  SGI1-B2 *Proteus mirabilis* PmSC17  SGI1-*Pm*SC1111 *Proteus mirabilis* PmSC1111  SGI1-*Pm*JN40 *Proteus mirabilis* JN40  SGI1-*Pm*CA11 *Proteus mirabilis* CA121511  *Salmonella enterica* Agona 89991  *Salmonella enterica* Albany R15.2267  SGI1-F *Salmonella enterica* Cerro SRC5  *Salmonella enterica* Derby FDA196946  *Salmonella enterica* Dublin OSF018603  SGI2 *Salmonella enterica* Emek SRC19  *Salmonella enterica* Enteritidis 92-0392  *Salmonella enterica* Hadar FNE0129  SGI1-D *Salmonella enterica* Infantis SRC46  *Salmonella enterica* Java PNCS013484  SGI1-K variant *Salmonella enterica* Kentucky 201001922  SGI1-K1 *Salmonella enterica* Kentucky SRC73  *Salmonella enterica* Kentucky BCW_2895  *Salmonella enterica* Kentucky PU131  *Salmonella enterica* Kentucky K13SK002  SGI1-XJ9S *Salmonella enterica* Kentucky XJ9S  *Salmonella enterica* Newport 193307  *Salmonella enterica* Saintpaul FNE0134  *Salmonella enterica* Senftenberg 199836  *Salmonella enterica* Senftenberg SAMEA5552168  **SGI1** *Salmonella enterica* Typhimurium 96-5227  *Salmonella enterica* Typhimurium FSIS11815820  *Salmonella enterica* Typhimurium NCTR350  *Salmonella enterica* Typhimurium 806209  *Salmonella enterica* Virchow 129420  *Salmonella enterica* Weltevreden 54-5182  SGI-NDM-1 *Vibrio cholerae* BRV8  *Vibrio cholerae* N2757  SGI1-Vc2CHAMA *Vibrio cholerae* 59-sc-2011-11-25T13  *Vibrio cholerae* 2011EL-1271  VGI *Vibrio cholerae* 2710-CN  *Vibrio cholerae* 555448 | 100.00 | 99.92 | 99.92 | 99.75 | 99.67 | 99.59 | 99.10 |
| *Klebsiella pneumoniae* 2018C01-046 | 99.92 | 100.00 | 99.84 | 99.67 | 99.59 | 99.51 | 99.01 |
| *Salmonella enterica* Panama BCW_2754 | 99.92 | 99.84 | 100.00 | 99.67 | 99.59 | 99.51 | 99.18 |
| *Vibrio cholerae* N2786  *Vibrio cholerae* N2726 | 99.75 | 99.67 | 99.67 | 100.00 | 99.92 | 99.84 | 98.85 |
| *Vibrio parahaemolyticus* 20140829008-1 | 99.67 | 99.59 | 99.59 | 99.92 | 100.00 | 99.92 | 98.77 |
| SGI1-V_SGI-V *Proteus mirabilis* VB1248  *Providencia stuartii* FDAARGOS_294 | 99.59 | 99.51 | 99.51 | 99.84 | 99.92 | 100.00 | 98.69 |
| SGI0 *Proteus mirabilis* Pm1LENAR | 99.10 | 99.01 | 99.18 | 98.85 | 98.77 | 98.69 | 100.00 |

**(3C)** *int* genes of SGI1-REs from the PGI1 cluster (cluster 2)**.**

| *int*_GI_ (% nt identity) | PGI1-*PmE*SC | PGI1-*Pm*PEL | *Sa* A3/19 | *Vc* SAMEA  4057619 | *Vc* 01-8_S93 | *Vn* VN-0515 | *Vc* BD21 | *Vm* SCCF01 | *Sh* KCT | *Ec* N63148 | *Se*M FSIS11814837 | *Vc* 9760 | *Se*I 355107 | *Se*M FSIS11706644 | *Vc* 10432-62 |
| --- | --- | --- | --- | --- | --- | --- | --- | --- | --- | --- | --- | --- | --- | --- | --- |
| **PGI1-*Pm*ESC** *Proteus mirabilis* PmESC  *Salmonella enterica* Agona WAPHL_SAL-A00509  PGI1-SL476 *Salmonella enterica* Heidelberg SL476  *Salmonella enterica* Heidelberg AZ-TG74856  *Salmonella enterica* Heidelberg BCW_3410  *Salmonella enterica* Infantis OSF018564  *Salmonella enterica* Muenster 26  *Salmonella enterica* Senftenberg CVM 34514 | 100.00 | 99.92 | 99.92 | 99.26 | 99.18 | 99.18 | 99.09 | 99.09 | 99.01 | 98.93 | 98.85 | 98.76 | 98.68 | 98.68 | 98.68 |
| PGI1-*Pm*PEL *Proteus mirabilis* PEL | 99.92 | 100.00 | 99.84 | 99.18 | 99.09 | 99.09 | 99.01 | 99.01 | 98.93 | 98.85 | 98.77 | 98.68 | 98.60 | 98.60 | 98.60 |
| *Shewanella algae* A3/19 | 99.92 | 99.84 | 100.00 | 99.18 | 99.09 | 99.09 | 99.01 | 99.01 | 98.93 | 98.85 | 98.76 | 98.68 | 98.60 | 98.60 | 98.60 |
| *Vibrio cholerae* SAMEA4057619 | 99.26 | 99.10 | 99.18 | 100.00 | 99.92 | 99.75 | 99.84 | 99.67 | 99.59 | 99.51 | 99.42 | 99.34 | 98.60 | 98.60 | 99.26 |
| *Vibrio cholerae* strain 01-8_S93 | 99.18 | 99.09 | 99.09 | 99.92 | 100.00 | 99.67 | 99.92 | 99.59 | 99.51 | 99.42 | 99.34 | 99.26 | 98.52 | 98.52 | 99.18 |
| *Vibrio navarrensis* VN-0515 | 99.18 | 99.09 | 99.09 | 99.75 | 99.67 | 100.00 | 99.59 | 99.59 | 99.51 | 99.42 | 99.34 | 99.26 | 98.52 | 98.52 | 99.18 |
| *Vibrio cholerae* BD21 | 99.09 | 99.01 | 99.01 | 99.84 | 99.92 | 99.59 | 100.00 | 99.51 | 99.42 | 99.34 | 99.26 | 99.18 | 98.44 | 98.44 | 99.09 |
| IME*Vm*SCCF01 *Vibrio mimicus* SCCF01 | 99.09 | 99.01 | 99.01 | 99.67 | 99.59 | 99.59 | 99.51 | 100.00 | 99.59 | 99.67 | 99.42 | 99.34 | 98.60 | 98.60 | 99.26 |
| *Shewanella* sp. KCT | 99.01 | 98.93 | 98.93 | 99.59 | 99.51 | 99.51 | 99.42 | 99.59 | 100.00 | 99.42 | 99.34 | 99.26 | 98.60 | 98.60 | 99.18 |
| *Escherichia coli* N63148 | 98.93 | 98.85 | 98.85 | 99.51 | 99.42 | 99.42 | 99.34 | 99.67 | 99.42 | 100.00 | 99.26 | 99.18 | 98.44 | 98.44 | 99.09 |
| *Salmonella enterica* Montevideo FSIS11814837  *Vibrio cholerae* PNUSAV000839  *Shewanella algae* Sh392  *Shewanella fodinae* 74A | 98.85 | 98.77 | 98.76 | 99.42 | 99.34 | 99.34 | 99.26 | 99.42 | 99.34 | 99.26 | 100.00 | 99.92 | 98.52 | 98.52 | 99.84 |
| *Vibrio cholerae* 9760  *Rheinheimera nanhaiensis* E407-8 | 98.76 | 98.68 | 98.68 | 99.34 | 99.26 | 99.26 | 99.18 | 99.34 | 99.26 | 99.18 | 99.92 | 100.00 | 98.44 | 98.44 | 99.75 |
| *Salmonella enterica* Infantis 355107 | 98.68 | 98.60 | 98.60 | 98.60 | 98.52 | 98.52 | 98.44 | 98.60 | 98.60 | 98.44 | 98.52 | 98.44 | 100.00 | 99.84 | 98.35 |
| *Salmonella enterica* Montevideo FSIS11706644 | 98.68 | 98.60 | 98.60 | 98.60 | 98.52 | 98.52 | 98.44 | 98.60 | 98.60 | 98.44 | 98.52 | 98.44 | 99.84 | 100.00 | 98.35 |
| IME*Vc*10432 *Vibrio cholerae* 10432-62 | 98.68 | 98.60 | 98.60 | 99.26 | 99.18 | 99.18 | 99.09 | 99.26 | 99.18 | 99.09 | 99.84 | 99.75 | 98.35 | 98.35 | 100.00 |

**(3D)** *int* genes of SGI1-REs from the PGI2 cluster (cluster 3)**.**

| *int*_GI_ (% nt identity) | PGI2 | *Vm* 2011V-1073 | *Vc* SA10G | *Kp*vST48_NDM | *Vc* 984-81 | *Ec* KK-NP016 |
| --- | --- | --- | --- | --- | --- | --- |
| *Cronobacter sakazakii* CFSAN019572  PGI2-*Ec*-2 *Escherichia coli* SCP17-71-2  PGI2-zym28 *Morganella morganii* zy_m28  **PGI2** *Proteus mirabilis* BC11-24  *Pseudomonas aeruginosa* MRSN1906 | 100.00 | 99.92 | 99.84 | 99.75 | 99.75 | 98.94 |
| *Vibrio mimicus* 2011V-1073  *Vibrio mimicus* 08-2414  *Vibrio parahaemolyticus* V67 | 99.92 | 100.00 | 99.92 | 99.84 | 99.84 | 99.02 |
| *Vibrio cholerae* SA10G | 99.84 | 99.92 | 100.00 | 99.75 | 99.75 | 98.94 |
| *Klebsiella pneumoniae* KpvST48_NDM  KGI *Klebsiella pneumoniae* AKPRH1296032 | 99.75 | 99.84 | 99.75 | 100.00 | 99.67 | 98.86 |
| *Vibrio cholerae* 984-81  *Vibrio cholerae* N2775 | 99.75 | 99.84 | 99.75 | 99.67 | 100.00 | 98.86 |
| *Escherichia coli* KK-NP016 | 98.94 | 99.02 | 98.94 | 98.86 | 98.86 | 100.00 |

**(3E)** *int* genes of SGI1-REs from the AGI1 cluster (cluster 4)**.**

| *int*_GI_ (% nt identity) | AGI1 | AGI3 *Se* Derby | *Vc*3017 | AGI2 EclC2185 | *V* sp. 2017V-1124 |
| --- | --- | --- | --- | --- | --- |
| AGI5 *Salmonella enterica* Cubana 76814  **AGI1** *Acinetobacter baumannii* D4 | 100.00 | 99.92 | 99.91 | 99.65 | 96.37 |
| AGI3_AGI1-C *Salmonella enterica* Derby 2014LSAL02547  AGI3 *Vibrio cholerae* 4874  *Vibrio cholerae* N2769 | 99.92 | 100.00 | 100.00 | 99.74 | 96.46 |
| *Vibrio cholerae* Vc3017  *Vibrio cholerae* 495747 | 99.91 | 100.00 | 100.00 | 99.74 | 96.46 |
| AGI2_AGI1-A *Enterobacter hormaechei* EclC2185  AGI1 variant *Escherichia coli* MOD1-EC6520  AGI4 *Klebsiella pneumoniae* K1781  AGI1 variant *Proteus mirabilis*  *Proteus mirabilis* PmBR607  AGI4 *Salmonella enterica* Agona 24.H.04  *Salmonella enterica* Infantis H124280339  AGI1 variant *Salmonella enterica* Stanley DMS 1112  AGI1-B *Vibrio cholerae* V060002  AGI2 *Vibrio cholerae* 133-73  *Vibrio fluvialis* GCCln30 | 99.65 | 99.74 | 99.74 | 100.00 | 96.72 |
| AGI *Vibrio* sp. 2017V-1124 | 96.37 | 96.46 | 96.46 | 96.72 | 100.00 |

**(3F)** *int* genes of SGI1-REs from cluster 5 (and SGI1, PGI1-*Pm*ESC, PGI2 and AGI1 used as reference).

| *int*_GI_ (% nt identity) | SGI1-RE5*Ss*W3-18-1 | *Sa* 150735 | *Sf* CG_2015-04_42_94 | *Sh* DC2-4 | *Vp* A1EZ952 | *Vc* VN-2808 | SGI1 | PGI2 | PGI1-*Pm*ESC | AGI1 |
| --- | --- | --- | --- | --- | --- | --- | --- | --- | --- | --- |
| SGI1-RE5*Ss*W3-18-1 *Shewanella* sp W3-18-1 | 100.00 | 99.18 | 98.36 | 98.28 | 97.95 | 97.54 | 94.99 | 82.27 | 79.90 | 64.34 |
| *Shewanella algae* 150735 | 99.18 | 100.00 | 98.85 | 98.77 | 98.44 | 98.03 | 94.83 | 82.18 | 79.82 | 64.59 |
| *Shewanella frigidimarina* CG_2015-04_42_94  *Shewanella vesiculosa* CG_2015-06_42_33  *Shewanella vesiculosa* CG_2015-09_42_97  *Shewanella* sp CG_4_9_14_0_8 | 98.36 | 98.85 | 100.00 | 99.92 | 99.59 | 97.70 | 95.07 | 82.51 | 80.07 | 64.85 |
| *Shewanella* sp. DC2-4 | 98.28 | 98.77 | 99.92 | 100.00 | 99.67 | 97.62 | 94.99 | 82.43 | 79.98 | 64.85 |
| *Vibrio parahaemolyticus* A1EZ952 | 97.95 | 98.44 | 99.59 | 99.67 | 100.00 | 97.29 | 94.75 | 82.18 | 79.74 | 64.68 |
| *Vibrio cholerae* VN-2808 | 97.54 | 98.03 | 97.70 | 97.62 | 97.29 | 100.00 | 94.09 | 82.59 | 80.07 | 64.51 |
| SGI1 | 94.99 | 94.83 | 95.07 | 94.99 | 94.75 | 94.09 | 100.00 | 81.61 | 79.40 | 64.42 |
| PGI2 | 82.27 | 82.18 | 82.51 | 82.43 | 82.18 | 82.59 | 81.61 | 100.00 | 94.73 | 63.30 |
| PGI1-*Pm*ESC | 79.90 | 79.82 | 80.07 | 79.98 | 79.74 | 80.07 | 79.40 | 94.73 | 100.00 | 63.13 |
| AGI1 | 64.34 | 64.59 | 64.85 | 64.85 | 64.68 | 64.51 | 64.42 | 63.30 | 63.13 | 100.00 |

**(3G)** *int* genes of SGI1-REs from clusters 6 and 7 (and SGI1, PGI1-*Pm*ESC, PGI2, AGI1 and SGI1-RE5*Ss*W3-18-1 from cluster 5 used as reference).

| *int*_GI_ (% nt identity) | SGI1-RE6*Id*CC-PW-9 | *Hm* Eplume2 | *I* REDSEA-S21_B4 | *M* SAT109 | SGI1-RE7*Ma*KG14 | PGI1-*Pm*ESC | PGI2 | SGI1-RE5*Ss*W3-18-1 | SGI1 | AGI1 |
| --- | --- | --- | --- | --- | --- | --- | --- | --- | --- | --- |
| SGI1-RE6*Id*CC-PW-9 *Idiomarina tyrosinivorans* CC-PW-9 | 100.00 | 99.92 | 99.84 | 89.60 | 89.52 | 75.47 | 74.61 | 72.26 | 72.10 | 62.18 |
| *Halomonas meridiana* Eplume2  *Marinobacter lutaoensis* T5054  *Pseudohongiella nitratireducens* CGMCC 1.15425  *Pseudohongiella nitratireducens* SCS-111 | 99.92 | 100.00 | 99.92 | 89.68 | 89.43 | 75.39 | 74.53 | 72.18 | 72.02 | 62.09 |
| *Idiomarina* sp. REDSEA-S21_B4 | 99.84 | 99.92 | 100.00 | 89.60 | 89.35 | 75.31 | 74.45 | 72.26 | 72.02 | 62.09 |
| *Marinobacter* sp. SAT109 (cluster 7) | 89.60 | 89.68 | 89.60 | 100.00 | 98.94 | 73.74 | 74.04 | 71.19 | 70.70 | 62.18 |
| SGI1-RE7*Ma*KG14 *Marinobacter* *adhaerens* KG14 (cluster 7) | 89.52 | 89.43 | 89.35 | 98.94 | 100.00 | 73.91 | 74.04 | 71.28 | 70.78 | 62.00 |
| PGI1-*Pm*ESC | 75.47 | 75.39 | 75.31 | 73.74 | 73.91 | 100.00 | 94.73 | 79.90 | 79.40 | 63.47 |
| PGI2 | 74.61 | 74.53 | 74.45 | 74.04 | 74.04 | 94.73 | 100.00 | 82.27 | 81.61 | 63.64 |
| SGI1-RE5*Ss*W3-18-1 | 72.26 | 72.18 | 72.26 | 71.19 | 71.28 | 79.90 | 82.27 | 100.00 | 94.99 | 64.68 |
| SGI1 | 72.10 | 72.02 | 72.02 | 70.70 | 70.78 | 79.40 | 81.61 | 94.99 | 100.00 | 64.68 |
| AGI1 | 62.18 | 62.09 | 62.09 | 62.18 | 62.00 | 63.47 | 63.64 | 64.68 | 64.68 | 100.00 |

**Supplementary Table 4. Percent Identity Matrix created by Clustal Omega for the backbones of SGI1-REs used as a reference in each cluster.**

| SGI1-RE backbone (% nt identity) | SGI1 | PGI2 | SGI1-RE5*Ss*W3-18-1 | AGI1 | PGI1-*Pm*ESC | SGI1-RE6*It*CC-PW-9 | SGI1-RE7*Ma*KG14 |
| --- | --- | --- | --- | --- | --- | --- | --- |
| SGI1 | 100.00 | 83.74 | 82.35 | 80.75 | 58.25 | 48.72 | 46.67 |
| PGI2 | 83.74 | 100.00 | 81.47 | 83.51 | 50.15 | 49.53 | 46.48 |
| SGI1-RE5*Ss*W3-18-1 | 82.35 | 81.47 | 100.00 | 89.28 | 49.00 | 55.65 | 47.30 |
| AGI1 | 80.75 | 83.51 | 89.28 | 100.00 | 47.30 | 53.55 | 46.72 |
| PGI1-*Pm*ESC | 58.25 | 50.15 | 49.00 | 47.30 | 100.00 | 45.41 | 42.52 |
| SGI1-RE6*It*CC-PW-9 | 48.72 | 49.53 | 55.65 | 53.55 | 45.41 | 100.00 | 75.17 |
| SGI1-RE7*Ma*KG14 | 46.67 | 46.48 | 47.30 | 46.72 | 42.52 | 75.17 | 100.00 |

**Supplementary Table 5**. **Median GC% of SGI1-REs hosts.**

| (Bacterial order)  Bacterial species | Median GC%* |
| --- | --- |
| (Enterobacteriales)  *Cronobacter sakazakii*  *Enterobacter hormaechei*  *Escherichia coli*  *Klebsiella pneumoniae*  *Morganella morganii*  *Proteus mirabilis*  *Providencia stuartii*  *Salmonella enterica* | 56.9  55.1  50.6  57.2  51  38.8  41.4  52.1 |
| (Pseudomonadales)  *Acinetobacter baumannii*  *Pseudomonas aeruginosa* | 39  66.2 |
| (Vibrionales)  *Vibrio cholerae*  *Vibrio fluvialis*  *Vibrio mimicus*  *Vibrio navarrensis*  *Vibrio parahaemolyticus*  *Vibrio* sp. | 47.5  50  46.3  48.4  45.3  44.5 |
| (Oceanospirillales)  *Halomonas meridiana* | 56.9 |
| (Alteromonadales)  *Idiomarina tyrosinivorans*  *Idiomarina* sp.  *Marinobacter adhaerens*  *Marinobacter lutaoensis*  *Marinobacter* sp.  *Shewanella algae*  *Shewanella fodinae*  *Shewanella frigidimarina*  *Shewanella putrefaciens*  *Shewanella vesiculosa*  *Shewanella* sp. | 49.3  47.2  55.9  63.3  57.2  53  49  41.35  44.5  41.7  44.05 |
| (Chromatiales)  *Rheinheimera nanhaiensis* | 51.3 |
| (Unclassified gamma-Proteobacteria)  *Pseudohongiella nitratireducens* | 51.8 |

* Data from the NCBI website: <https://www.ncbi.nlm.nih.gov/genome/>

**Supplementary Table 7. Names of the new SGI1-REs analyzed in this study based on the backbone modifications.**

| GI name^a^ | Strains^b^ | Accession number^c^ | Name of new SGI1-REs^d^ |
| --- | --- | --- | --- |
| SGI1  SGI1-D  SGI1-*PmJ*N40  SGI1-B2  SGI1-F  VGI  SGI1-*Pm*2CHAMA  SGI1-*Vc*2CHAMA  SGI1-*Pm*CA11  SGI-NDM-1  SGI2  SGI1-Z  SGI1-B-*Ec*1  SGI1-*Pm*SC1111  SGI1-*Pm*MAT  SGI1-L  SGI1-K1  SGI1-K variant  SGI1-XJ9S  SGI1-V (SGI-V)  SGI0  SGI1-LK1 | *Salmonella enterica* Typhimurium 96-5227  *Salmonella enterica* Infantis SRC46  *Salmonella enterica* Enteritidis 92-0392  *Salmonella enterica* Dublin OSF018603  *Salmonella enterica* Panama BCW_2754  *Salmonella enterica* Java PNCS013484  *Proteus mirabilis* JN40  *Salmonella enterica* Derby FDA196946  *Vibrio cholerae* 2011EL-1271  *Proteus mirabilis* PmSC17  *Salmonella enterica* Agona 89991  *Salmonella enterica* Cerro SRC5  *Salmonella enterica* Albany R15.2267  *Salmonella enterica* Senftenberg SAMEA5552168  *Vibrio cholerae* 555448  *Vibrio cholerae* 2710-CN  *Proteus mirabilis* Pm2CHAMA  *Vibrio cholerae* 59-sc-2011-11-25T13  *Salmonella enterica* Saintpaul FNE0134  *Proteus mirabilis* CA121511  *Vibrio cholerae* BRV8  *Salmonella enterica* Emek SRC19  *Salmonella enterica* Virchow 129420  *Proteus mirabilis* PmSC42  *Salmonella enterica* Senftenberg 199836  *Escherichia coli* AVC96  *Proteus mirabilis* PmSC1111  *Klebsiella pneumoniae* 2018C01-046  *Salmonella enterica* Newport 193307  *Proteus mirabilis* PmMAT  *Morganella morganii* Pr5  *Salmonella enterica* Kentucky SRC73  *Salmonella enterica* Kentucky 201001922  *Salmonella enterica* Typhimurium FSIS11815820  *Salmonella enterica* Kentucky K13SK002  *Salmonella enterica* Weltevreden 54-5182  *Salmonella enterica* Kentucky PU131  *Vibrio cholerae* N2786  *Salmonella enterica* Kentucky XJ9S  *Salmonella enterica* Typhimurium NCTR350  *Proteus mirabilis* VB1248  *Providencia stuartii* FDAARGOS_294  *Proteus mirabilis* Pm1LENAR  *Vibrio cholerae* N2726  *Salmonella enterica* Kentucky BCW_2895  *Salmonella enterica* Hadar FNE0129  *Salmonella enterica* Typhimurium 806209  *Vibrio parahaemolyticus* 20140829008-1  *Proteus mirabilis* Pm294MATLI  *Vibrio cholerae* N2757 | AF261825  KU854986  CP018657  AAQBBL010000010  MYAK01000099/4/781/962  AAXPUJ010000014  MF576128  AAIUNV010000014  CP046839  KP116299  AAJPHB010000015  KU847976  CP065564  DAAHPW010000016  AAXNYA010000016  MN708015  MF372716  MN708014  AAQALN010000014/15  MH990673  CTBD01000091  AY963803  AALLNJ010000009  KP057606  AAIEYO010000017  MK599281  MH998665  CP044368  AALJQG010000018  JX089583  LT630458  AY463797  CP028357  AAHSTR010000012  CP037917  MZGE01000033/781  CP026327  VSHP01000003  MN640065  NQWV01000039/27  HQ888851  NJEY02000002  MH734355  VSFX01000005  MXVV01000018  AANCMD010000013  AALNAT010000020  CP034294  MH734354  VSGQ01000007 | SGI1-RE1-*S*Ty1  SGI1-RE1-*S*Ty1  SGI1-RE1-*S*Ty1  SGI1-RE1-STy1  SGI1-RE1-*S*Ty1  SGI1-RE1-*S*Ty1  SGI1-*Pm*JN40  SGI1-RE1-*S*Ty1  SGI1-RE1-*S*Ty1  SGI1-B2  SGI1-RE1-STy1  SGI1-RE1-*S*Ty1  SGI1-RE1-*S*Ty1  SGI1-RE1-*S*Ty1  SGI1-RE1-*S*Ty1  VGI  SGI1-*Pm*2CHAMA  SGI1-*Vc*2CHAMA  SGI1-*Pm*2CHAMA  SGI1-*Pm*CA11  SGI1-*Pm*CA11  SGI2  SGI1-RE1-*S*Vi2  SGI1-Z  SGI1-RE1-*S*Se3  SGI1-B-*Ec*1  SGI1-*Pm*SC1111  SGI1-RE1-*Kpn*4  SGI1-RE1-*S*Ne5  SGI1-*Pm*MAT  SGI1-L  SGI1-K1  SGI1-K1  SGI1-RE1-*S*Ty6  SGI1-RE1-*S*Ke7  SGI1-RE1-*S*Ke7  SGI1-RE1-*S*Ke8  SGI1-RE1-*Vch*9  SGI1-XJ9S  SGI1-RE1-*S*Ty10  SGI1-V  SGI1-V  SGI0  SGI1-RE1-*Vch*11  SGI1-RE1-SKe12  SGI1-RE1-*S*Ha13  SGI1-RE1-SHa13  SGI1-RE1-*Vpa*14  SGI1-LK1  SGI1-RE1-*Vch*15 |
| PGI1-*Pm*ESC  PGI1-SL476  PGI1-*Pm*PEL  IMEVc10432-62  IME*Vm*SCCF01 | *Proteus mirabilis* PmESC  *Salmonella enterica* Muenster 26  *Salmonella enterica* Infantis OSF018564  *Salmonella enterica* Agona WAPHL_SAL-A00509  *Salmonella enterica* Heidelberg SL476  *Proteus mirabilis* PEL  *Salmonella enterica* Senftenberg CVM 34514  *Salmonella enterica* Heidelberg AZ-TG74856  *Shewanella algae* A3/19  *Salmonella enterica* Heidelberg BCW_3410  *Salmonella enterica* Montevideo FSIS11706644  *Salmonella enterica* Infantis 355107  *Vibrio cholerae* 9760  *Salmonella enterica* Montevideo FSIS11814837  *Vibrio cholerae* BD21  *Vibrio cholerae* 01-8_S93  *Vibrio navarrensis* VN-0515  *Vibrio cholerae* PNUSAV000839  *Vibrio cholerae* 10432-62  *Shewanella algae* Sh392  *Shewanella fodinae* 74A  *Rheinheimera nanhaiensis* E407-8  *Vibrio cholerae* SAMEA4057619  *Shewanella* sp. KCT  *Escherichia coli* N63148  *Vibrio mimicus* SCCF01 | KU499917  QDTO01000013  AAQBCE010000021/23  AAFZIB010000022/24  CP001120  KF856624  CP051329  AAFUZP010000001  JACDTT010000025/49  MXSU01000026/6  AANGCS010000016  AAMIAE010000014  RHMB01000014  ROHT01000031  QEEE01000038  QZUV01000027  MPKK01000014  AAXNBC010000015/39  CP010812  QFDC01000022/74/49  SLWF01000006  BAFK01000015  DACPGR010000009  LVDJ01000007  NTMS01000010  CP016383 | SGI1-RE2-*Pmi*1  SGI1-RE2-*Pmi*1  SGI1-RE2-*Pmi*1  SGI1-RE2-*Pmi*1  SGI1-RE2-*Pmi*1  PGI1-*Pm*PEL  SGI1-RE2-*S*Se2  SGI1-RE2-*S*Se2  SGI1-RE2-*Sal*3  SGI1-RE2-*S*He4  SGI1-RE2-*S*Mo5  SGI1-RE2-*S*In6  SGI1-RE2-*Vch*7  SGI1-RE2-*Vch*7  SGI1-RE2-*Vch*8  SGI1-RE2-*Vch*9  SGI1-RE2-*Vna*10  SGI1-RE2-*Vch*11  SGI1-RE2-*Vch*12  SGI1-RE2-*Vch*12  SGI1-RE2-*Sfo*13  SGI1-RE2-*Rna*14  SGI1-RE2-*Vch*15  SGI1-RE2-Ssp16  SGI1-RE2-*Eco*17  SGI1-RE2-*Vmi*18 |
| PGI2  PGI2-zym28  PGI2‐*Ec*-2  KGI | *Proteus mirabilis* BC11-24  *Morganella morganii* zy_m28  *Escherichia coli* SCP17-71-2  *Pseudomonas aeruginosa* MRSN1906  *Cronobacter sakazakii* CFSAN019572  *Vibrio cholerae* SA10G  *Vibrio parahaemolyticus* V67  *Vibrio mimicus* 2011V-1073  *Vibrio mimicus* 08-2414  *Klebsiella pneumoniae* AKPRH1296032  *Klebsiella pneumoniae* KpvST48_NDM  *Vibrio cholerae* 984-81  *Vibrio cholerae* N2775  *Escherichia coli* KK-NP016 | MG201402  MW080367  MN708013  RXVB01000034/21  AAXWFR010000026/21  CP053820  JABCDZ010000015  CP035682  JACGMF010000003  MN708012  VCEE01000001  JMBM01000011  VSHF01000011/12  BFWH01000021 | SGI1-RE3-*Pmi*1  SGI1-RE3-*Pmi*1  PGI2‐*Ec*-2  SGI1-RE3-*Pmi*1  SGI1-RE3-*Csa*2  SGI1-RE3-*Vch*3  SGI1-RE3-*Vpa*4  SGI1-RE3-*Vmi*5  SGI1-RE3-*Vmi*5  KGI  SGI1-RE3-*Kpn*6  SGI1-RE3-*Vch*9  SGI1-RE3-*Vch*9  SGI1-RE3-*Eco*10 |
| AGI1  AGI4  AGI4  AGI1 variant  AGI1 variant  AGI1 variant  AGI3 (AGI1-C)  AGI  AGI5  AGI3  AGI1-B  AGI2 (AGI1-A)  AGI2 | *Acinetobacter baumannii* D4  *Salmonella enterica* Agona 24.H.04  *Klebsiella pneumoniae* k1781  *Salmonella enterica* Infantis H124280339  *Salmonella enterica* Stanley DMS 1112  *Vibrio fluvialis* GCCln30  *Proteus mirabilis*  *Escherichia coli* MOD1-EC6520  *Vibrio cholerae* 495747  *Salmonella enterica* Derby 2014LSAL02547  *Vibrio* sp. 2017V-1124  *Salmonella enterica* Cubana 76814  *Vibrio cholerae* 4874  *Vibrio cholerae* Vc3017  *Vibrio cholerae* N2769  *Vibrio cholerae* V060002  *Enterobacter hormaechei* EclC2185  *Vibrio cholerae* 133-73  *Proteus mirabilis* PmBR607 | KP054476  CASR01000049/44  FLJQ01000020/22  AAFGGL010000026/29  MXLM01000016  JACNEY010000015  MK422178  NLVV01000018/47/28  AAXOLK010000011  CP029486  QKKN01000025/42  AYUE01000008/66  NOJI01000025/29/33  JAEMFW010000021  VSHA01000017/3  AP018677  MH545561  JIDK01000013  CP049753 | SGI1-RE4-*Aba*1  SGI1-RE4-*S*Ag2  SGI1-RE4-*S*Ag2  SGI1-RE4-*S*Ag2  SGI1-RE4-*S*St3  SGI1-RE4-*S*St3  SGI1-RE4-SSt3  SGI1-RE4-*S*St3  SGI1-RE4-*Vch*4  SGI1-RE4-SDe5  SGI1-RE4-*V*sp6  SGI1-RE4-*S*Cu7  SGI1-RE4-*Vch*8  SGI1-RE4-*Vch*8  SGI1-RE4-*Vch*9  SGI1-RE4-*Vch*10  SGI1-RE4-*Eho*11  SGI1-RE4-*Eho*11  SGI1-RE4-*Pmi*12 |
| SGI1-RE5*Ss*W3-18-1 | *Shewanella* sp. W3-18-1  *Shewanella algae* 150735  *Shewanella* sp. DC2-4  *Vibrio parahaemolyticus* A1EZ952  *Shewanella* sp. CG_4_9_14_0_8  *Shewanella frigidimarina* CG_2015-04_42_94  *Shewanella vesiculosa* CG_2015-06_42_33  *Shewanella vesiculosa* CG_2015-09_42_97  *Vibrio cholerae* VN-2808 | CP000503  CP068229  JABRVR010000002  LRTI01000059  PFTI01000029  JAACRB010000031  JAACRN010000013  JAACSM010000021/26  MCBB01000198/211/210/160 | SGI1-RE5-*S*sp1  SGI1-RE5-*Sal*2  SGI1-RE5-*S*sp3  SGI1-RE5-*Vpa*4  SGI1-RE5-*S*sp5  SGI1-RE5-*S*sp5  SGI1-RE5-*S*sp5  SGI1-RE5-*Sve*6  SGI1-RE5-*Vch*7 |
| SGI1-RE6*It*CC-PW-9 | *Idiomarina tyrosinivorans* CC-PW-9  *Marinobacter lutaoensis* T5054  *Idiomarina* sp. REDSEA-S21_B4  *Halomonas meridiana* Eplume2  *Pseudohongiella nitratireducens* SCS-111  *Pseudohongiella nitratireducens* CGMCC 1.15425 | PIQH01000002  MSCW01000004  LUNZ01000035/12  AP022869  LWHN01000034/30/29  BMIY01000006 | SGI1-RE6-*Ity*1  SGI1-RE6-*Mlu*2  SGI1-RE6-*I*sp3  SGI1-RE6-*Hme*4  SGI1-RE6-*Pni*5  SGI1-RE6-*Pni*6 |
| SGI1-RE7MaKG14 | *Marinobacter adhaerens* KG14  *Marinobacter* sp. SAT109 | JABEVQ010000005/1  PAYG01000030/41 | SGI1-RE7-*Mad*1  SGI1-RE7-*M*sp2 |

^a^ The SGI1-REs used as a reference in each cluster are underlined.

^b^ Clusters (n=7) are based on the homology of SGI1-REs integrase gene (nt homology >97%, corresponding to AA homology >98%).

^c^ Multiple accession numbers are shown for backbones in multiple unassembled contigs.

^d^ The names of the complete and published SGI1-REs have been kept. Only the names of the SGI1-REs based on the MDR region have been renamed. The SGI1-REs used as a reference in each cluster have been renamed for consistency. Backbones with the same modifications as published in another bacterial species have the same name to be consistent with them and allow comparison of backbones. Since the error rate in whole-genome sequencing is about 0.1%, backbones with ≥0.2% nt substitutions were named differently.
